# The Effect of 4-(Dimethylamino)phenyl-5-oxopyrrolidines on Breast and Pancreatic Cancer Cell Colony Formation, Migration, and Growth of Tumor Spheroids

**DOI:** 10.3390/ijms25031834

**Published:** 2024-02-02

**Authors:** Karolina Kairytė, Rita Vaickelionienė, Birutė Grybaitė, Kazimieras Anusevičius, Vytautas Mickevičius, Vilma Petrikaitė

**Affiliations:** 1 Department of Organic Chemistry, Kaunas University of Technology, Radvilėnų Rd. 19, LT-50254 Kaunas, Lithuania; karolina.kairyte@ktu.lt (K.K.); rita.vaickelioniene@ktu.lt (R.V.); birute.grybaite@ktu.lt (B.G.); kazimieras.anusevicius@ktu.lt (K.A.); vytautas.mickevicius@ktu.lt (V.M.); 2 Institute of Biotechnology, Life Sciences Center, Vilnius University, Saulėtekio Al. 7, LT-10257 Vilnius, Lithuania; 3 Faculty of Medicine, Lithuanian University of Health Sciences, A. Mickevičiaus 9, LT-44307 Kaunas, Lithuania; 4 Laboratory of Drug Targets Histopathology, Institute of Cardiology, Lithuanian University of Health Sciences, Sukilėlių Pr. 13, LT-50162 Kaunas, Lithuania

**Keywords:** triple-negative breast cancer, pancreatic cancer, cell viability, cell migration, clonogenic assay, tumor spheroid

## Abstract

A series of hydrazones, azoles, and azines bearing a 4-dimethylaminophenyl-5-oxopyrrolidine scaffold was synthesized. Their cytotoxic effect against human pancreatic carcinoma Panc-1 and triple-negative breast cancer MDA-MB-231 cell lines was established by MTT assay. Pyrrolidinone derivatives **3c** and **3d**, with incorporated 5-chloro and 5-methylbenzimidazole fragments; hydrazone **5k** bearing a 5-nitrothien-2-yl substitution; and hydrazone **5l** with a naphth-1-yl fragment in the structure significantly decreased the viability of both cancer cell lines. Compounds **3c** and **5k** showed the highest selectivity, especially against the MDA-MB-231 cancer cell line. The EC_50_ values of the most active compound **5k** against the MDA-MB231 cell line was 7.3 ± 0.4 μM, which were slightly higher against the Panc-1 cell line (10.2 ± 2.6 μM). Four selected pyrrolidone derivatives showed relatively high activity in a clonogenic assay. Compound **5k** was the most active in both cell cultures, and it completely disturbed MDA-MB-231 cell colony growth at 1 and 2 μM and showed a strong effect on Panc-1 cell colony formation, especially at 2 μM. The compounds did not show an inhibitory effect on cell line migration by the ‘wound-healing’ assay. Compound **3d** most efficiently inhibited the growth of Panc-1 spheroids and reduced cell viability in MDA-MB-231 spheroids. Considering these different activities in biological assays, the selected pyrrolidinone derivatives could be further tested to better understand the structure–activity relationship and their mechanism of action.

## 1. Introduction

Despite the progress in treating various types of cancer, the cancer incidence is increasing worldwide. One of the most aggressive types of cancer is pancreatic adenocarcinoma, which leads to the highest mortality [1]. Unfortunately, it is often asymptomatic, and patients are not treated until the later stages when the treatment is ineffective [2]. In this regard, the US Congress considers pancreatic ductal adenocarcinoma ‘a resistant cancer’. Therefore, the Pancreatic Cancer Research and Education Act was formulated to detect cancer most effectively in its early stages and treat it [3]. Triple-negative breast cancer is characterized by the absence of estrogen, progesterone, and HER-2 receptors and is considered the most aggressive and metastatic type of breast cancer [4]. Both of these cancer types are known to often acquire multi-drug resistance; therefore, existing chemotherapeutic agents are used in combination with novel drugs to increase treatment effectiveness. In addition, researchers are searching for new molecules that may help to overcome cancer resistance [5,6].

**Figure 1:**
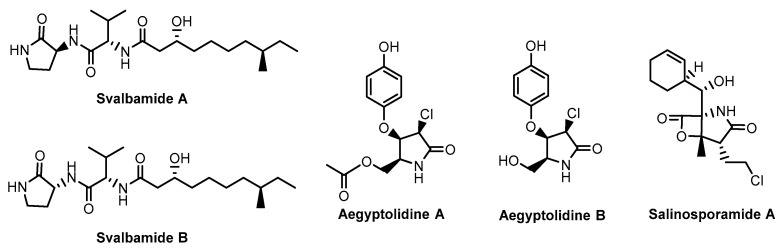
Some 2-pyrrolidinones of natural origin.

2-Pyrrolidinone, structurally constructed from a five-membered lactam, is a constituent of a large variety of natural and synthetic healing products. Natural 2-pyrrolidinone-based *Svalbamides A* and *B*, which were isolated from a culture extract of *Paenibacillus* sp. taken from the Svalbard archipelago in the Arctic Ocean, induced quinone reductase activity in Hepa1c1c7 murine hepatoma cells, indicating their potential as chemopreventive agents [7]. Alkaloids aegyptolidines A and B were isolated from the fungus *Aspergillus aegyptyacus* and have cytotoxic activity [8], and Salinosporamide A is a marine-derived proteasome inhibitor [9] (Figure 1). Furthermore, the 2-pyrrolidinone scaffold is considered an essential group of pharmacophores, which, when incorporated into complex chemical structures, give compounds a broad spectrum of pharmacological properties, such as anti-inflammatory [10], antimicrobial [11], HIV-1 integrase inhibition [12], anticonvulsant [13], and cardioprotective [14] properties, making them suitable for therapeutic purposes. The simplicity and versatility of the 2-pyrrolidinone core make it an interesting starting material for a wide range of pharmaceutical and medicinal applications. Therefore, the synthesis and evaluation of the biological activity of compounds containing this structural scaffold remain among the most attractive and rapidly developing fields of medicinal chemistry for the design and discovery of efficient therapeutic preparations [15].

Extensive research and many scientific publications have demonstrated that the 4-dimethylaminophenyl fragment is versatile for drug discovery. The studies that have been performed regarding this moiety showed that it can provide compound bioactivity or increase it. Chalcones bearing a dimethylaminophenyl fragment have been reported as potential inhibitors of nitric oxide and prostaglandin E_2_ production in the RAW 264.7 macrophage cell line [16]. The presence of 4-dimethylaminophenyl moieties as ring-B in chalcones exhibited maximum activity in various antioxidant assays [17]. Moreover, such derivatives have been proven to possess significant anti-inflammatory [18] and anticancer [19] properties, the highest HDAC inhibitory capacity, and the strongest antiproliferative activity [20].

**Figure 2:**
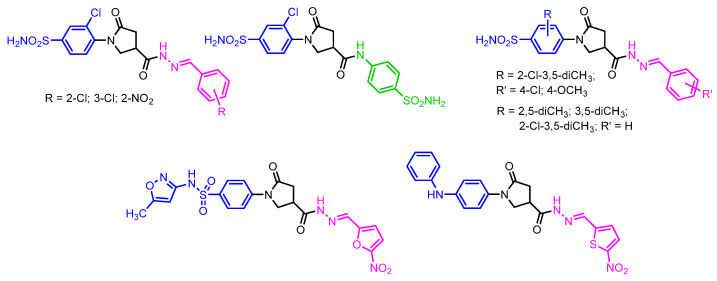
Some of the 2-pyrrolidinone-based compounds with anticancer properties that have been obtained and evaluated by the research group to which the authors belong.

Based on the aforementioned findings and our previous experience in the synthesis and anticancer evaluation of 2-pyrrolidinone derivatives [21,22,23,24,25] (Figure 2), we aimed to synthesize a new series of compounds bearing the 1-(4-(dimethylamino)phenyl)pyrrolidin-2-yl moiety and to explore their anticancer properties. This goal was achieved, and a focused library of novel substituted 2-pyrrolidinone derivatives was obtained from acid hydrazide by using standard synthesis methods adapted to the preparation of hydrazide derivatives.

The compound effect was tested both in 2D and 3D cell culture models. As the primary and most widely used technique, the effect on cell viability was chosen in order to establish the most active cytotoxic compounds. Then, their effect was evaluated using a clonogenic assay, allowing us to assess their ability to reduce the formation of the most aggressive remaining cancer cells after treatment, which could form new tumors after metastasis [26]. Moreover, to prevent metastasis formation, it is important to reduce cells’ ability to migrate; thus, we applied the ‘wound-healing’ assay [27]. Finally, the compound activity was established in 3D cell cultures—tumor spheroids, which have become a popular model, were used as a bridge between common cell monolayers and further experiments in vivo [28]. Overall, we aimed to evaluate the compound potential to be developed as anticancer agents in selected pancreatic Panc-1 and triple-negative breast cancer MDA-MB-231 cellular models.

## 2. Results and Discussion

### 2.1. Chemistry

**Scheme 1:**
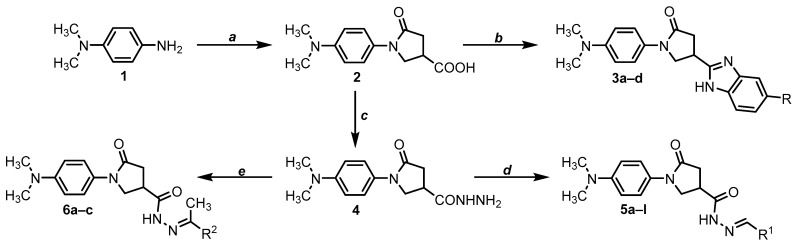
Synthesis of derivatives **2**–**6**: **3**: **a** R = H, **b** R = F, **c** R = Cl, **d** R = CH_3_; **5**: **a** R^1^ = C_6_H_5_, **b** R^1^ = 4-ClC_6_H_4_, **c** R^1^ = 4-BrC_6_H_4_, **d** R^1^ = 4-(H_3_C)_2_NC_6_H_4_, **e** R^1^ = 4-H_3_COC_6_H_4_, **f** R^1^ = 2,5-di(H_3_CO)C_6_H_3_, **g** R^1^ = 2,4,6-tri(H_3_CO)C_6_H_2_, **h** R^1^ = 3,4,5-tri(H_3_CO)C_6_H_2_, **i** R^1^ = 4-O_2_NC_6_H_4_, **j** R^1^ = 2-thienyl, **k** R^1^ = 5-nitro-2-thienyl, **l** R^1^ = 1-naphthyl; **6**: **a** R^2^ = CH_3_, **b** R^2^ = C_2_H_5_, **c** R^2^ = 4-H_2_NC_6_H_4_. Reagents and conditions: **a** itaconic acid, water, Δ, 24 h; **b** the corresponding *o*-phenylenediamine, 4M HCl, Δ, 24 h; 5% Na_2_CO_3_; **c** N_2_H_4_∙H_2_O, toluene, Δ, 16 h; **d** hydrazide + water + HCl, corresponding aldehyde + propan-2-ol, Δ, 2 h; **e** the corresponding ketone, AcOH, Δ, 18 h or 5 h.

In this study *p*-(dimethylamino)aniline (**1**) was used as an initial compound for the synthesis of target compounds **2**–**11** by the known procedures [23,24,29], which were adapted to obtain the desired compounds. First, 1-(4-(dimethylamino)phenyl)-5-oxopyrrolidine-3-carboxylic acid (**2**) was obtained. The resynthesized acid **2** [30] was applied in reactions with various *o*-phenylenediamines by the most commonly used Phillip’s procedure [31], which gave 2,5-disubstituted benzimidazoles **3a**–**d** (Supplementary Materials, Figures S1–S8). The reflux of **2** in toluene with excess hydrazine monohydrate afforded hydrazide **4** (Supplementary Materials, Figures S9 and S10), thus avoiding the additional esterification reaction and shortening the synthesis of the target hydrazide (Scheme 1). To produce anticipated hydrazones **5a**–**l** and **6a**–**c**, the prepared acid hydrazide **4** was condensed with a corresponding aldehyde, including a series of substituted phenyl, 1-naphthyl, as well as carbaldehydes, or reacted with the corresponding ketone, i.e., acetone, methylethylketone (MEK), or 4′-aminoacetophenone, respectively.

The synthesis was performed by refluxing in a mixture of water/propan-2-ol (10:1) for 2 h using hydrochloric acid as a catalyst. The obtained hydrazones **5a**–**l** were separated in 48–94% yields. As expected, the NMR spectra of these compounds revealed the presence of conformers [23] caused by the restricted rotation around the amide C(O)–NH bond. The presence of a mixture of *Z*/*E* isomers is notably reflected in the ^1^H NMR spectra where the signals of the NH proton for each isomer give rise in the characteristic field of 11.17–11.97 ppm (Supplementary Materials, Figures S11–S34) of this spectrum, and the peaks of the prevailing *Z*-rotamers are visible in the up-field part of the spectrum in comparison with the *E* one [23]. The intensity ratio of the amide conformers was quantified from ^1^H NMR spectra based on the integral values of the two separate peaks of rotamer.

At this stage of the work, we did not set the goal of separating the *Z*-isomer from the *E*-form and studying the anticancer properties of each of them, so they were not separated. This is planned to be completed in the next phases of the study. To expand the variety of hydrazones and identify the remarkable anticancer activity of the hydrazones obtained from the reactions with various ketones [32,33,34], hydrazide **4** was reacted with some of them. The reactions with acetone (**6a**) and methylethylketone (**6b**) ended in 18 h. For 4′-aminoacetophenone (**6c**), the reaction occurred in 1,4-dioxane for 5 h. The target product *N*′-(1-(4-aminophenyl)ethylidene)-1-(4-(dimethylamino)phenyl)-5-oxopyrrolidine-3-carbohydrazide (**6c**) was isolated in 78% yield. The spectral data of compounds **6a**–**c** fully confirmed their structures (Supplementary Materials, Figures S35–S39).

**Scheme 2:**
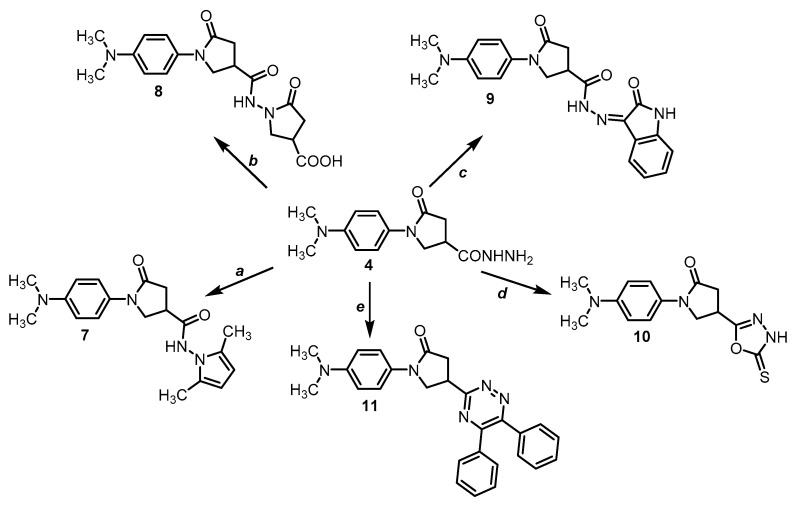
Synthesis of azoles **7**–**10** and 1,2,4-triazine **11**. Reagents and conditions: **a** hexane-2,5-dione, i-PrOH, AcOH, Δ, 18 h; **b** itaconic acid, water, Δ, 24 h; **c** isatin, MeOH, AcOH, Δ, 4 h; **d** KOH, MeOH, CS_2_, hydrazide + MeOH, Δ, 7 h, water, AcOH to pH 6; **e** 1,2-diphenylethane-1,2-dione, AcOH, AcONH_4_, Δ, 24 h, water.

To achieve azole derivatives **7**–**10** (Scheme 2), acid hydrazide **4** was applied to reactions with the appropriate agent. By our previously improved procedure [23], the treatment of **4** with hexane-2,5-dione (2.5 eqv.) in propan-2-ol using glacial acetic acid as a catalyst gave 2,5-dimethylpyrrole **7** in 64% yield. In the ^13^C NMR spectrum of compound **7**, the resonances of the carbons of the methyl groups and the CH–CH fragment of the 2,5-dimethylpyrrole ring arose at 8.77 and 10.94 ppm, and 103.06 ppm, respectively. Peaks at 170.68 and 172.05 were assigned to the carbons of two carbonyl groups of the molecule. The ^1^H NMR spectrum confirmed the existence of the 2,5-dimethylpyrrole moiety, showing singlets at 1.94, 1.98, and 1.99 ppm and peaks at 5.48 and 5.64 ppm for the protons of the aforementioned moieties (Supplementary Materials, Figures S40 and S41). The NH proton signal was observed as a set of two singlets due to the presence of an amide moiety in this structure and the restricted rotation around it.

As a compound having an amine group, hydrazide **4** was used in the aza-Michael addition reaction, which involves the interaction between itaconic acid and primary amines and subsequent cascade intramolecular cyclization to form a pyrrolidinone cycle [35]. Heating at the reflux of reactants in water for 24 h, yielded compound **8** with an additional pyrrolidinone cycle in the structure. Although the molecule can exist as a mixture of *E*/*Z* rotamers through the amide bond, the experimental NMR data demonstrated only one isomer in the DMSO-*d*_6_ solution. The ^1^H NMR spectrum of **8** showed a 2-fold increase in the number of pyrrolidinone protons, which confirms the formation of the second pyrrolidinone cycle. The same is true for the carbon resonances of the pyrrolidinone ring: the ^13^C NMR spectrum demonstrates six spectral lines at 31.27, 33.74, 34.12, 35.15, 49.65, and 50.73 ppm for the carbons of two pyrrolidinone cycles. In the ^1^H NMR spectrum, the presence of an amide and OH groups was confirmed by the singlets at 10.34 and 12.77 ppm, respectively. Four carbonyl groups of the target compound were confirmed by the carbon resonances at 170.72, 170.90, 171.54, and 174.04 ppm in the ^13^C NMR spectrum of the molecule (Supplementary Materials, Figures S42 and S43) 

Compound **9** was obtained by refluxing hydrazide **4** with isatin in methanol in the presence of acetic acid as a catalyst [24]. The ^1^H NMR spectrum revealed additional peaks in the aromatic field of the spectrum as well as a singlet at 10.82 ppm, which belong to the proton of the NH group of the newly attached oxoindoline moiety. The ^13^C NMR spectrum showed characteristic carbonyl resonances at 159.21, 164.53, and 170.81 ppm (Supplementary Materials, Figures S44 and S45).

1,3,4-Oxadiazole derivative **10** was prepared by the slightly improved method [36] in the reaction of acid hydrazide **4** with carbon disulfide in the presence of potassium hydroxide in refluxing 2-propanol with subsequent heterocyclization by acidifying the resulting aqueous dithiocarbazate solution with acetic acid to pH 6., In the ^1^H NMR spectrum, all the protons were seen according to the expected chemical structure. The aromatic protons appeared at 6.72 and 7.40 ppm as two doublets with *J* = 8.8 Hz, while the protons of the pyrrolidinone ring exhibited multiplets in the ranges of 2.74–2.95 (CH_2_CO), 3.88–3.96 (CH), and 3.96–4.16 (NCH_2_) ppm. An intense singlet integrated for six protons at 2.86 ppm was assigned to 2CH_3_, and a broad singlet at 13.89 ppm showed the presence of NH in the oxadiazole ring. The presence of the 1,3,4-oxadiazolethione cycle confirmed the characteristic resonance line at 178 ppm in the ^13^C NMR spectrum of the compound, which was attributed to the carbon of the C=S group (Supplementary Materials, Figures S46 and S47).

For 1,2,4-triazine derivative **11,** the cyclocondensation of hydrazide **4** with an α-dicarbonyl compound was used [37]. Thus, the treatment of **4** with an equimolar amount of 1,2-diphenylethane-1,2-dione in glacial acetic acid in the presence of a large excess of ammonium acetate afforded 1-(4-(dimethylamino)phenyl)-4-(5,6-diphenyl-1,2,4-triazin-3-yl)pyrrolidin-2-one (**11**). The ^1^H NMR of this compound showed a large increase in aromatic protons in the interval of 7.37–7.53 ppm, which was assigned to the protons of two additional phenyl rings. In the ^13^C NMR spectrum, the spectral lines observed at 155.91, 156.09, and 167.04 ppm are characteristic of the C-5, C-6, and C-3 atoms of the 1,2,4-triazine cycle (Supplementary Materials, Figures S48 and S49).

It is noteworthy that due to the asymmetric carbon in the pyrrolidinone ring, the molecules with this carbon are also asymmetric. The X-ray crystallographic analysis data of 5-oxo-1-(*p*-tolyl)pyrrolidine-3-carboxylic acid showed that its crystals are a racemic mixture of *R*- and *S*-enantiomers in which the ratio of enantiomers is 1:1, and they form mixed crystals [38]. The exchange of *R,S*-enantiomers in the crystal structure is chaotic, and such a structure is accepted as disordered. It is likely that the synthesized compounds containing the pyrrolidinone ring are also racemic mixtures of enantiomers in the crystalline state. Thus, it can be stated that analogous compound **2** of our study and its derivatives have the same composition.

All synthesized compounds were then applied for the evaluation of their biological properties.

### 2.2. Pharmacology

#### 2.2.1. Cytotoxicity

**Figure 3:**
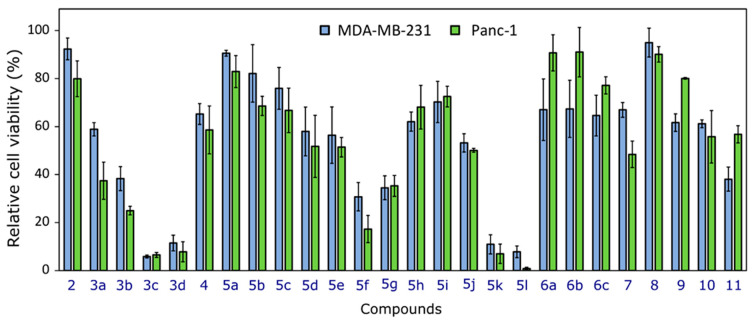
Effect of compounds on cancer cell viability at 100 µM concentration after 72 h of incubation against human triple-negative breast cancer MDA-MB-231 and human pancreatic carcinoma Panc-1 cell lines by MTT assay, *n* = 3.

The tested compounds at a 100 μM concentration showed different activity on human triple-negative breast cancer MDA-MB-231 and human pancreatic carcinoma Panc-1 cell viability (Figure 3). This concentration was chosen based on compound solubility and our experience. It has been shown that concentrations from 50 to 100 μM allow for distinguishing the most active compounds [39] from a series, and thus, they are often used for primary screening [40].

In general, there was a correlation between the activities of compounds against both cell lines. However, the obtained results were not surprising, as triple-negative and pancreatic cancer cells usually possess lower sensitivity compared with other types of cancer [41].

The structure–activity relationship study of the investigated compounds **2**–**11** showed that the anticancer activity of benzimidazoles **3a***–***d** increased upon introducing electron-withdrawing groups such as 5-fluoro and, especially, 5-chloro. The replacement of hydrogen with fluorine in compound **3b** was successful in increasing the activity; however, the high electronegativity of fluorine influenced a weaker effect than the less electronegative chlorine in compound **3c**, resulting in a significant decrease in cell viability. Surprisingly, the attached electron-donating 5-methyl group (**3d**) greatly elevated cell suppression.

Regarding hydrazide **4** and its hydrazone derivatives **5a***–***l**, the incorporation of Ph, 4-ClPh, 4-BrPh, 3,4,5-tri(H_3_CO)Ph, and 4-O_2_NPh substituents decreased their activity, while 4-(H_3_C)_2_NC_6_H_4_, 4-H_3_COC_6_H_4,_ and thien-2-yl had a slightly better anticancer effect than hydrazide **4**. The most efficient in this hydrazone series appeared to be compound **5l** with a bulky 1-naphthyl substitution. Next, in descending order of effect for both cancer cells were hydrazones **5k**, **5f**, and **5g**, with 5-nitro-2-thienyl, 2,5-di(H_3_CO)Ph, and 2,4,6-tri(H_3_CO)Ph substituents in the structure. As seen from the assay data, the activity of hydrazones **6a**–**c** is very weak, but the attached larger fragment of 4-H_2_NPh slightly reduced the viability of both cells. Among the azole derivatives **7**–**10** and azine **11**, the best anticancer properties against the pancreatic cell line Panc-1 were shown by 2,5-dimethylpyrrole derivative **7**, while 1,2,4-triazine derivative **11** had the strongest effect on human triple-negative breast cancer cells MDA- MB-231.

Four compounds (**3c**, **3d**, **5k**, and **5l**) were the most active against both cell lines. Pyrrolidinone derivatives **3c** and **3d**, incorporating 5-chloro and 5-methylbenzimidazole fragments, respectively, along with hydrazone **5k** bearing a 5-nitrothien-2-yl substitution and hydrazone **5l** with a naphth-1-yl fragment in the structure, significantly decreased the viability of triple-negative breast cancer and ductal pancreatic carcinoma cell lines.

**Figure 4:**
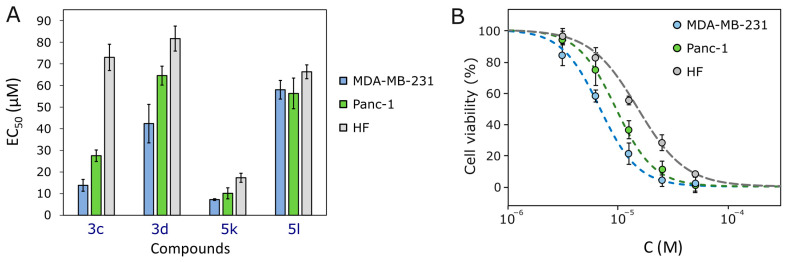
EC_50_ values of the most active compounds **3c**, **3d**, **5k**, and **5l** obtained by MTT assay (**A**) against triple-negative breast cancer MDA-MB231, pancreatic cancer Panc-1 cell lines, and human fibroblasts (HF) after 72 h of incubation. Dose–response curves for the most active compound **5k** after 72 h of incubation with MDA-MB-231, Panc-1, and HF cells (**B**). Data points are experimental values (averages of three repeats), while the lines are the fit of the standard inhibition model with the Hill coefficient of 2.5. *n* = 3.

Further studies determined their effective concentrations that reduce cell viability by 50% (EC_50_ values), as illustrated in Figure 4A.

The naphth-1-yl fragment-bearing compound **5l** demonstrated a lack of selectivity against cancer cell lines. Due to its relatively low impact on pancreatic and breast cancer cell viability, it is not considered a very promising candidate for further development. Nevertheless, we aimed to explore its effects on cell migration and activity in 3D cultures for comparison purposes with the other selected compounds. Compounds **3c** and **5k** exhibited the highest selectivity, particularly against the MDA-MB-231 cancer cell line (the selectivity ratios were 0.2 and 0.4 against the MDA-MB-231 cell line and 0.4 and 0.6 against the Panc-1 cell line, respectively), making them worthy of further development. Compound **3d** was less active and relatively less selective (selectivity towards MDA-MB-231 was 0.5 and 0.8 toward Panc-1 cells). The EC_50_ value of the most active compound **5k** against the MDA-MB-231 cell line was 7.3 ± 0.4 μM, and it was slightly higher against the Panc-1 cell line (10.2 ± 2.6 μM) (Figure 4B). Both compounds **3c** and **3d** reduced MDA-MB-231 cell viability more efficiently than Panc-1 (by 2 times and 1.5 times, respectively).

Pancreatic and triple-negative breast cancer types are characterized as relatively resistant to chemotherapy [42,43]. In our previous studies, we also observed that these two types of cancer cells are usually less responsive to treatment with chemical compounds [25]. Considering the relatively low activity of the tested oxopyrrolidine derivatives (EC_50_ values ranging from 7.3 to even 65 μM), it is crucial to assess their potential selectivity against cancer cells compared to fibroblasts to avoid possible toxicity in vivo.

Despite this desirable characteristic, the range of selectivity ratios can vary based on the compound, the type of cancer, and many other factors. However, there are divergent opinions on this issue, as not only selectivity but also other characteristics determine the overall suitability of a compound to become an effective anticancer drug. These factors include pharmacokinetic properties, bioavailability, safety profile, side effects, advantages over existing therapies, etc. [44]. These properties are studied in later developmental stages, and at the beginning, the most potent candidates are usually selected based on their potency, bearing in mind that nanotechnology could help to overcome their unfavorable properties [45].

Thus, in our study, the most active four compounds were all selected for further detailed studies in migration, clonogenic assay, and cancer cell 3D cultures.

#### 2.2.2. Effect on Cell Colony Formation

**Figure 5:**
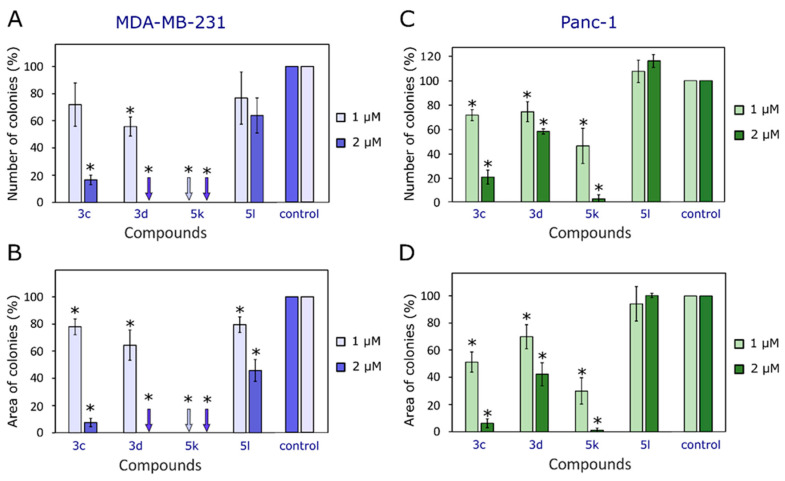
Effect of compounds **3c**, **3d**, **5k**, and **5l** on cell colony formation (**A**) and growth (**B**) of the human triple-negative breast cancer MDA-MB-231 cell line and cell colony formation (**C**) and growth (**D**) of the human pancreatic ductal carcinoma cell line, *n* = 3. Asterisks (*) indicate *p* < 0.05 compared with the control (untreated cells).

Selected pyrrolidone derivatives showed relatively high activity on cell colony formation by reducing their number and disturbing their growth (Figure 5). In general, compound **5l** did not affect Panc-1 and MDA-MB-231 cell colony formation at either tested concentration (Figure 5A,C); only the MDA-MB-231 colonies were more compact compared with the control (Figure 5B). Compound **5k** was the most active in both cell cultures, and it completely disturbed MDA-MB-231 cell colony growth at the 1 and 2 μM concentrations and showed a strong colony formation disturbing effect in Panc-1 cells, especially at 2 μM (Figure 5C). It is worth noting that compound **3d** at 2 μM possessed different activity against MDA-MB-231 and Panc-1 cell colony formation and growth: it completely prohibited MDA-MB-231 cell colony formation, while the effect on Panc-1 cell colony formation and growth was comparable to the 1 μM concentration.

These findings show that tested pyrrolidone derivatives have varying degrees of activity on cell colony formation. Considering their selective effects based on different concentrations and cell lines, it would be worthwhile to further investigate their dose–response relationship and mechanism of action. The observed effects of compounds contribute to a better understanding of their long-term effects (not seen in the 3-day MTT assay) and also provide information on how well these compounds could prevent the long-term survival and proliferation of cancer cells in the tumor microenvironment [46]. Interestingly, despite the fact that compound **3c** after 72 h incubation was more cytotoxic against MDA-MB-231 cells compared with compound **3d** (Figure 4), after 7 days, it showed similar or even slightly lower activity compared to compound **3d** (Figure 5A,B). This observation suggests that both compounds could have a different mechanism of action. In addition, both of them possessed similar activity towards prohibiting the cell colony formation and growth of both cell lines, despite their cytotoxicity differing by 1.5–2 times.

The compound effect on cell colony formation differs greatly and is dependent on various factors (cell type, compound concentration, duration of incubation, pretreatment, or constant incubation with tested compound and others) and can be challenging. Thus, the comparison of the clonogenic assay results with other compounds from other experiments should be performed very cautiously, paying much attention to the experimental setup. For example, the anticancer drug gemcitabine, which is approved for monotherapy or in combination for several types of cancers (breast cancer, non-small cell lung cancer, pancreatic cancer, etc.), at 10 μM concentration reduced Panc-1 cell colony formation up to approximately 40% but showed only a relatively small effect (<10%) at 1 μM concentration [47]. However, this experiment lasted for two weeks, and the number of seeded cells was 1000 in a 6-well plate (compared with 200 in a 12-well plate). Doxorubicin (an anthracycline class drug used for the treatment of triple-negative breast cancer) at 70 nM concentration reduces MDA-MB-231 cell colony formation up to approximately 5% after 21 days of incubation [48]. However, even higher effects on cell colony growth could be achieved by combining several compounds with different mechanisms of action [49]. We hypothesize that our tested pyrrolidone derivatives, especially compound **5k**, could be further studied in combinations with anticancer agents.

#### 2.2.3. Effect on Cell Migration

The compound effect on cell migration was established by ‘wound-healing’ assay in both cell lines after 24, 48, and 72 h of incubation. MDA-MB-231 cells are characterized as very invasive cell lines, and they migrate very fast. Thus, we established the compounds’ effect on migration up to 48 h.

**Figure 6:**
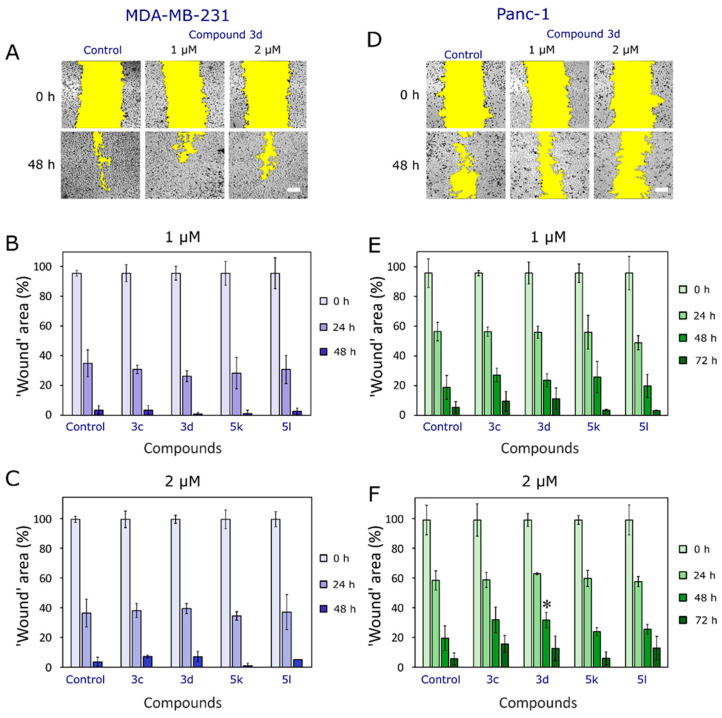
Effect of compounds **3c**, **3d**, **5k**, and **5l** on human triple-negative MDA-MB-231 and human pancreatic Panc-1 cell migration determined by ‘wound-healing’ assay. Photos of the ‘wound’ area (marked in yellow) in the MDA-MB-231 (**A**) and Panc-1 (**D**) monolayer at the beginning and the end of the experiment. The ‘wound’ area of MDA-MB-231 cells treated with 1 µM (**B**) and 2 µM (**C**) of the tested compounds at the end of the experiment, *n* = 3. The ‘wound’ area of Panc-1 cells treated with 1 µM (**E**) and 2 µM (**F**) of the tested compounds at the end of the experiment, *n* = 3. The scale bar indicates 100 µm. Asterisks (*) indicate *p* < 0.05 compared with the control (untreated cells).

The compounds did not show an inhibitory effect on MDA-MB-231 cell line migration at either concentration. Compounds **3c** and **3d** were the most active in the Panc-1 cell line, but only **3d** at 2 µM had a statistically significant effect after 48 h of incubation (Figure 6). Surprisingly, despite the fact that compound **5k** was the most cytotoxic compound and the most active one in the clonogenic assay, it did not show an effect on the migration of either cell line.

In the ‘wound-healing’ assay, we used concentrations of compounds that did not show cytotoxic effects at 72 h to avoid the misinterpretation of lower cell proliferation (and thus a wider remaining ‘wound’) as an inhibitory effect on cell migration. These relatively low concentrations of compounds could be too low to see the effect on migration. In this case, other methods like single-cell migration could be more useful [50].

#### 2.2.4. Compound Effect in 3D Cultures

Three-dimensional cell models are shown to be more representative compared to cell monolayers (the two-dimensional model) [51]. Thus, we made cancer cell spheroids to conduct the experiments in more realistic conditions. For these studies, spheroids of a diameter > 250 µm were used, which are known to have a hypoxic region inside [52]. Spheroids were made from cancer cells and fibroblasts at a ratio of 1:1 to better mimic the tumor microenvironment, considering that a tumor has stroma, which is involved in tumor development and metastasis.

**Figure 7:**
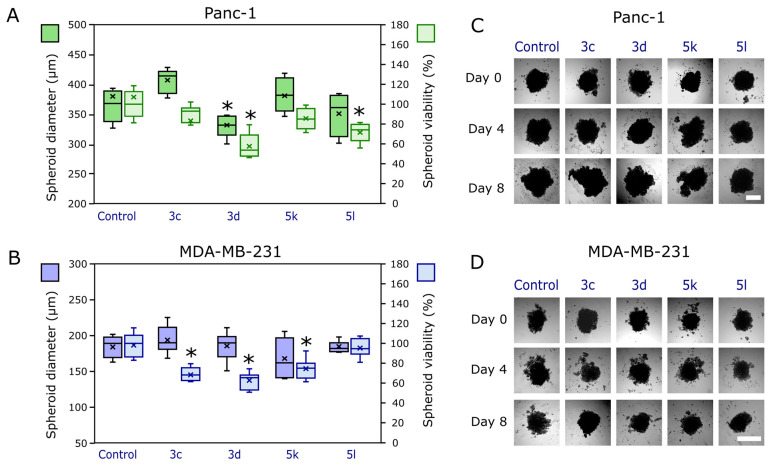
Effect of compounds **3c**, **3d**, **5k**, and **5l** on 3D cell cultures. (**A**) Human pancreatic carcinoma Panc-1 spheroid size (brighter green color) and viability of cells in spheroids (lighter green color) at the end of the experiment. (**B**) Human triple-negative breast cancer MDA-MB-231 spheroid size (brighter blue color) and viability of cells in spheroids (lighter blue color) at the end of the experiment. (**C**) Photos of Panc-1 tumor spheroids at the beginning (Day 0), in the middle (Day 4), and at the end (Day 8) of the experiment (after incubation with 10 µM of the compounds). (**D**) Photos of MDA-MB-231 tumor spheroids at the beginning (Day 0), in the middle (Day 4), and at the end (Day 8) of the experiment (after incubation with 10 µM of the compounds). Asterisks (*) indicate *p* < 0.05 compared with the control (untreated spheroids); crosses (×) indicate means; inner dashes indicate medians; and whiskers indicate maximum and minimum values. Scale bars indicate 200 µm.

The effect of 10 µM of the compounds in spheroids was evaluated over a period of eight days (Figure 7).

It is well known that evaluating the effect of a compound only by comparing the spheroid size change is not entirely correct as it does not necessarily correlate with cell viability inside the spheroids [53,54]. Thus, at the end of the experiment, spheroid cell viability was established. The viability of spheroids treated with compounds **3c**, **3d**, and **5k** was up to appr. 45% lower compared with the control despite the differences in the sizes of the spheroids not being very large. This difference could be explained by the differences in spheroid morphology and also possible variable compound concentration gradients inside due to hypoxia or different densities.

Overall, the tested pyrrolidone derivatives were more active in MDA-MB-231 3D cultures than in Panc-1 (Figure 7). Compound **5k**, which was the most cytotoxic compound, did not show a statistically significant effect on Panc-1 spheroid growth and viability, and its activity was similar to **3c** and **3d**. Compound **5l** was the least active and reduced only Panc-1 spheroid viability up to appr. 20% compared with the control. Compound **3d** most efficiently inhibited the growth of Panc-1 spheroids and also reduced cell viability in MDA-MB-231 spheroids. These findings suggest that the selected pyrrolidone derivatives could act by different mechanisms, and we hypothesize that some of them (especially compound **3d**) could act on targets expressed in hypoxia, which was at the core of the formed spheroid. Of course, this hypothesis has to be proven by more detailed studies.

In summary, pyrrolidone derivatives **3c**, **3d**, and **5k** have been identified as the most promising compounds in this series. They are relatively selective against the MDA-MB-231 cell line. In the case of triple-negative breast cancer, **3d** and **5k** were shown to reduce cell colony formation and also reduce spheroid growth and viability. In the pancreatic cell line, **3d** showed the highest activity in 3D cell culture; however, **5k** was the most cytotoxic and exhibited the highest effect on cell colony formation and growth.

## 3. Materials and Methods

### 3.1. Chemistry

Reagents and solvents were obtained from Sigma–Aldrich (St. Louis, MO, USA) and used without further purification. The reaction course and purity of the synthesized compounds were monitored by TLC using aluminum plates precoated with silica gel with F254 nm (Merck KGaA, Darmstadt, Germany). Melting points were determined with a B-540 melting point analyzer (Büchi Corporation, New Castle, DE, USA) and were uncorrected. NMR spectra were recorded on a Brucker Avance III (400, 101 MHz) spectrometer (Bruker BioSpin AG, Fällanden, Switzerland). Chemical shifts were reported in (δ) ppm relative to tetramethylsilane (TMS) with the residual solvent as internal reference (DMSO-d_6_, δ = 2.50 ppm for ^1^H and δ = 39.52 ppm for ^13^C). Data were reported as follows: chemical shift, multiplicity, coupling constant (Hz), integration, and assignment. IR spectra (ν, cm^−1^) were recorded on a Perkin–Elmer Spectrum BX FT–IR spectrometer (Perkin–Elmer Inc., Waltham, MA, USA) using KBr pellets. Elemental analyses (C, H, N) were conducted using an Elemental Analyzer CE-440 (Exeter Analytical, Inc., Chelmsford, MA, USA); their results were found to be in good agreement (±0.3%) with the calculated values.

1-(4-(Dimethylamino)phenyl)-5-oxopyrrolidine-3-carboxylic acid (**2**) was resynthesized [30], and the obtained data of the compound was in good agreement with the original one.

General procedure for the preparation of benzimidazoles **3a**–**d**

A mixture of compound **2** (0.7 g, 2.8 mmol) and corresponding o-phenylenediamine (0.6 g, 5.6 mmol) was refluxed in aqueous 18.5% HCl (6 mL) for 24 h and then cooled and neutralized with aqueous 5% sodium carbonate solution. The mixture was boiled and cooled, and then the water layer was decanted, and the residue was washed twice with boiling water. After cooling, the formed precipitate was filtered off and washed with water to give the title compounds **3a**–**d**.

4-(1*H*-benzo[d]imidazol-2-yl)-1-(4-(dimethylamino)phenyl)pyrrolidin-2-one (**3a**)

Brown solid, yield 0.54 g, 60%, m. p. 220–221 °C (from propan-2-ol:water, 1:1).

IR (KBr): ν 3422 (NH); 1694 (C=O); 1520 (C=N); 1164; 1112 (C-N) cm^–1^.

^1^H NMR (400 MHz, DMSO–d_6_) δ 2.86 (s, 6H, 2CH_3_), 2.91–2.99 (m, 2H, CH_2_CO), 3.91–4.05 (m, 1H, CH), 4.10–4.24 (m, 2H, NCH_2_), 6.73 (d, *J* = 8.6 Hz, 2H, H_Ar_), 7.11–7.19 (m, 2H, H_Ar_), 7.46 (d, *J* = 8.6 Hz, 3H, H_Ar_), 7.58 (d, *J* = 7.5 Hz, 1H, H_Ar_), 12.47 (s, 1H, NH) ppm.

^13^C NMR (101 MHz, DMSO–d_6_) δ 30.81, 37.31 (CH_2_CO, CH), 40.34 (2CH_3_), 52.57 (NCH_2_), 111.05, 112.39, 118.48, 121.15, 121.23, 121.95, 128.96, 134.57, 142.83, 147.60, 155.16 (C_Ar_), 171.05 (C=O) ppm.

Calcd. for C_19_H_20_N_4_O, %: C 71.23; H 6.29; N 17.49; found, %: C 71.19; H 6.25; N 17.46.

1-(4-(Dimethylamino)phenyl)-4-(5-fluoro-1*H*-benzo[d]imidazol-2-yl)pyrrolidin-2-one (**3b**)

Brown solid, yield 0.39 g, 41%, m. p. 108–109 °C (from propan-2-ol:water 1:1).

IR (KBr): ν 3398 (NH); 1671 (C=O); 1521 (C=N); 1136; 1111 (C-N) cm^–1^.

^1^H NMR (400 MHz, DMSO–d_6_) δ 2.86 (s, 6H, 2CH_3_), 2.90–3.05 (m, 2H, CH_2_CO), 3.89–4.04 (m, 1H, CH), 4.06–4.25 (m, 2H, NCH_2_), 6.73 (d, *J* = 8.6 Hz, 2H, H_Ar_), 6.95–7.05 (m, 1H, H_Ar_), 7.28–7.56 (m, 2H, H_Ar_ + 7.45 (d, *J* = 8.6 Hz, 2H, H_Ar_), 12.60 (s, 1H, NH) ppm.

^13^C NMR (101 MHz, DMSO–d_6_) δ 30.83, 37.23 (CH_2_CO, CH), 40.35 (2CH_3_), 52.49 (NCH_2_), 112.39, 121.24, 128.93, 147.62 (C_Ar_), 170.99 (C=O) ppm.

Calcd. for C_19_H_19_FN_4_O, %: C 67.44; H 5.66; N 16.56; found, %: C 67.39; H 5.69; N 16.52.

1-(4-(Dimethylamino)phenyl)-4-(5-chloro-1*H*-benzo[d]imidazol-2-yl)pyrrolidin-2-one (**3c**)

Dark green solid, yield 0.93 g, 94%, m. p. 90–91 °C (from propan-2-ol:water, 1:1).

IR (KBr): ν 3371 (NH); 1671 (C=O); 1520 (C=N); 1168; 1128 (C-N) cm^–1^.

^1^H NMR (400 MHz, DMSO–d_6_) δ 2.86 (s, 6H, 2CH_3_), 2.91–3.01 (m, 2H, CH_2_CO), 3.91–4.05 (m, 1H, CH), 4.08-4.28 (m, 2H, NCH_2_), 6.72 (d, *J* = 8.6 Hz, 2H, H_Ar_), 7.15–7.20 (m, 1H, H_Ar_), 7.44 (d, *J* = 8.6 Hz, 2H, H_Ar_), 7.48–7.62 (m, 2H, H_Ar_), 12.69 (s, 1H, NH) ppm.

^13^C NMR (101 MHz, DMSO–d_6_) δ 30.80, 37.20 (CH_2_CO, CH), 40.34 (2CH_3_), 52.45 (NCH_2_), 112.38, 121.25, 128.90, 147.62 (C_Ar_), 170.95 (C=O) ppm.

Calcd. for C_19_H_19_ClN_4_O, %: C 64.31; H 5.40; N 15.79; found, %: C 64.38; H 5.45; N 15.82.

1-(4-(Dimethylamino)phenyl)-4-(5-methyl-1*H*-benzo[d]imidazol-2-yl)pyrrolidin-2-one (**3d**) 

Brown solid, yield 0.56 g, 60%, m. p. 118–119 °C (from propan-2-ol/water 1:1).

IR (KBr): ν 3351 (NH); 1674 (C=O); 1520 (C=N); 1167; 1115 (C-N) cm^–1^.

^1^H NMR (400 MHz, DMSO–d_6_) δ 2.38, 2.40 (2s, 3H, CH_3_), 2.86 (s, 6H, 2CH_3_), 2.89–2.99 (m, 2H, CH_2_CO), 3.90–3.99 (m, 1H, CH), 4.09–4.22 (m, 2H, NCH_2_), 6.73 (d, *J* = 8.6 Hz, 2H, H_Ar_), 6.93–7.00 (m, 1H, H_Ar_), 7.20–7.42 (m, 2H, H_Ar_), 7.45 (d, *J* = 8.6 Hz, 2H, H_Ar_), 12.28, 12.31 (2s, 1H, NH) ppm.

^13^C NMR (101 MHz, DMSO–d_6_) δ 21.29 (CH_3_), 30.81, 37.32 (CH_2_CO, CH), 40.35 (2CH_3_), 52.59 (NCH_2_), 112.39, 121.22, 128.97, 147.59 (C_Ar_), 171.07 (C=O) ppm.

Calcd. for C_20_H_22_N_4_O, %: C 71.83; H 6.63; N 16.75; found, %: C 71.79; H 6.65; N 16.75.

1-(4-(Dimethylamino)phenyl)-5-oxopyrrolidine-3-carbohydrazide (**4**) 

A mixture of compound **2** (2.48 g, 0.01 mol), hydrazine monohydrate (1.5 g, 0.03 mol), and toluene (50 mL) was refluxed for 16 h and then cooled. The obtained crystalline solid was filtered off and washed with propan-2-ol to give the title compound **4**.

Light violet solid, yield 1.52 g, 58%, m. p. 225–226 °C (from propan-2-ol).

IR (KBr): ν 3398-3229 (NH+ NH_2_); 1671; 1657 (C=O); 1226; 1198 (C-N) cm^–1^.

^1^H NMR (400 MHz, DMSO–d_6_) δ 2.55–2.68 (m, 2H, CH_2_CO), 2.86 (s, 6H, 2CH_3_), 3.15 (p, *J* = 8.2 Hz, 1H, CH), 3.74–3.91 (m, 2H, NCH_2_), 4.27 (br. s, 2H, NH_2_), 6.71 (d, *J* = 8.7 Hz, 2H, H_Ar_), 7.40 (d, *J* = 8.7 Hz, 2H, H_Ar_), 9.36 (s, 1H, NH) ppm.

^13^C NMR (101 MHz, DMSO–d_6_) δ 34.16, 35.48, 40.35, 51.07 (CH, CH_2_CO, N(CH_3_)_2_, NCH_2_), 112.39, 121.17, 128.89, 147.59 (C_Ar_), 171.10, 171.62 (2C=O) ppm.

Calcd. for C_13_H_18_N_4_O_2_, %: C 59.53; H 6.92; N 21.36; found, %: C 59.55; H 6.89; N 21.32.

General procedure for the preparation of hydrazones **5a**–**l**

To a solution of hydrazide **4** (0.52 g 2 mmol) in water (50 mL) with the addition of concentrated hydrochloric acid (5 drops), the solution of the corresponding aromatic aldehyde (2.7 mmol) in propan-2-ol (5 mL) was added, and the mixture was heated at reflux for 2 h. After completion of the reaction, the mixture was cooled, and the formed precipitate was filtered off and washed with water to give the corresponding compounds **5a**–**l**.

*N*′-benzylidene-1-(4-(dimethylamino)phenyl)-5-oxopyrrolidine-3-carbohydrazide (**5a**) 

White solid, yield 0.34 g, 48%, m. p. 228–229 °C (from 1,4-dioxane).

IR (KBr): ν 3251 (NH); 1643; 1611 (C=O); 1510 (C=N); 1178; 1100 (C-N) cm^–1^.

^1^H NMR (400 MHz, DMSO–d_6_) δ (*Z*/*E* 65/35): 2.68–2.79 (m, 2H, CH_2_CO), 2.86 (s, 6H, 2CH_3_), 3.65–4.35 (m, 3H, NCH_2_+CH), 6.72 (dd, *J* = 9.4, 3.7 Hz, 2H, H_Ar_), 7.38–7.46 (m, 5H, H_Ar_), 7.70 (dd, *J* = 7.1, 2.1 Hz, 2H, H_Ar_), 8.04, 8.22 (2s, 1H, N=CH), 11.56, 11.62 (2s, 1H, NH) ppm.

^13^C NMR (101 MHz, DMSO–d_6_) δ 32.94, 34.68, 34.93, 35.41 (CH_2_CO, CH), 40.34 (2CH_3_), 20.47, 50.89 (NCH_2_), 112.39, 121.22, 121.28, 126.87, 127.09, 128.86, 128.92, 129.90, 130.11, 134.15, 143.58, 146.98, 147.61, 168,81, 170.90, 171.09, 173.71 (C_Ar_, 2C=O) ppm.

Calcd. for C_20_H_22_N_4_O_2_, %: C 68.55; H 6.33; N 15.99; found, %: C 68.59; H 6.35; N 15.96.

*N*′-(4-chlorobenzylidene)-1-(4-(dimethylamino)phenyl)-5-oxopyrrolidine-3-carbohydrazide (**5b**) 

White solid, yield 0.37 g, 48%, m. p. 230–231 °C (from 1,4-dioxane).

IR (KBr): ν 3274 (NH); 1655; 1627 (C=O); 1527 (C=N); 1175; 1113 (C-N) cm^–1^.

^1^H NMR (400 MHz, DMSO–d_6_) δ (*Z*/*E* 65/35): 2.59–2.75 (m, 2H, CH_2_CO), 2.86 (s, 6H, 2CH_3_), 3.28–3.34 (M, 0.35H, CH), 3.92–4.09 (m, 2.65H, NCH_2_+CH), 6.72 (dd, *J* = 9.4, 3.7 Hz, 2H, H_Ar_), 7.37–7.55 (m, 4H, H_Ar_), 7.73 (dd, *J* = 7.1, 2.1 Hz, 2H, H_Ar_), 8.01, 8.21 (2s, 1H, N=CH), 11.62 11.68 (2s, 1H, NH) ppm.

^13^C NMR (101 MHz, DMSO–d_6_) δ 32.90, 34.67, 34.92, 35.29 (CH_2_CO, CH), 40.34 (2CH_3_), 50.41, 50.85 (NCH_2_), 112.38, 121.21, 121.27, 128.54, 128.72, 128.92, 133.10, 134.30, 142.30, 145.65, 147.61, 168.90, 170.86, 171.04, 173.77 (C_Ar_, 2C=O) ppm.

Calcd. for C_20_H_21_ClN_4_O_2_, %: C 62.42; H 5.50; N 14.56; found, %: C 62.47; H 5.47; N 14.57.

*N*′-(4-bromobenzylidene)-1-(4-(dimethylamino)phenyl)-5-oxopyrrolidine-3-carbohydrazide (**5c**) 

White solid, yield 0.81 g, 94%, m. p. 275–276 °C (from 1,4-dioxane).

IR (KBr) ν 3274 (NH); 1710; 1656 (C=O); 1527 (C=N); 1175; 1143 (C-N) cm^–1^.

^1^H NMR (400 MHz, DMSO–d_6_) δ (*Z*/*E* 65/35): 2.62–2.77 (m, 2H, CH_2_CO), 2.86 (s, 6H, 2CH_3_), 3.28–3.31 (m, 0.4H, CH), 3.81–4.14 (m, 2.6H, NCH_2_+CH), 6.71 (d, *J* = 8.8 Hz, 2H, H_Ar_), 7.43 (d, *J* = 8.7 Hz, 2H, H_Ar_), 7.42–7.82 (m, 4H, H_Ar_), 8.00, 8.19 (2s, 1H, N=CH), 11.62, 11.69 (2s, 1H, NH) ppm.

^13^C NMR (101 MHz, DMSO–d_6_) δ 34.66, 34.92, 35.38 (CH_2_CO, CH), 40.34 (2CH_3_), 50.41, 50.84 (NCH_2_), 112.38, 121.21, 121.26, 123.07, 123.33, 128.76, 128.94, 131.82, 133.44, 142.40, 145.73, 147.60, 168.90, 170.86, 171.03, 173.77 (C_Ar_, 2C=O) ppm.

Calcd. for C_20_H_21_BrN_4_O_2_, %: C 55.95; H 4.93; N 13.05; found, %: C 55.99; H 4.94; N 13.08.

*N*′-((4-dimethylamino)benzylidene)-1-(4-(dimethylamino)phenyl)-5-oxopyrrolidine-3-carbohydrazide (**5d**) 

White solid, yield 0.46 g, 58%, m. p. 241–242 °C (from 1,4-dioxane).

IR (KBr) ν 3202 (NH); 1659; 1644 (C=O); 1518 (C=N); 1181; 1168, 1146 (C-N) cm^–1^.

^1^H NMR (400 MHz, DMSO–d_6_) δ (*Z*/*E* 65/35): 2.66–2.75 (m, 2H, CH_2_CO), 2.94 (s, 6H, 2CH_3_), 3.03, 3.04 (2s, 6H, 2CH_3_), 3.32–3.38 (m, 0.35H, CH), 3.92–4.16 (m, 2.65H, NCH_2_+CH), 6.75–6.87 (m, 4H, H_Ar_), 7.51 (d, *J* = 8.9 Hz, 2H, H_Ar_), 7.54–7.64 (m, 2H, H_Ar_), 7.97, 8.14 (2s, 1H, N=CH), 11.34, 11.39 (2s, 1H, NH) ppm.

^13^C NMR (101 MHz, DMSO–d_6_) δ 32.95, 34.66, 34.87, 35.49 (CH_2_CO, CH), 40.34 (2CH_3_), 50.57, 51.01 (NCH_2_), 111.78, 111.83, 112.39, 121.19, 121.26, 128.10, 128.41, 128.87, 128.96, 144.34, 147.59, 147.75, 151.36, 151.52, 168.14, 170.98, 171.18, 173.05 (C_Ar_, 2C=O) ppm.

Calcd. for C_22_H_27_N_5_O_2_, %: C 67.15; H 6.92; N 17.80; found, %: C 67.19; H 6.95; N 17.78.

*N*′-(4-methoxybenzylidene)-1-(4-(dimethylamino)phenyl)-5-oxopyrrolidine-3-carbohydrazide (**5e**) 

White solid, yield 0.37 g, 48%, m. p. 187–188 °C (from 1,4-dioxane).

IR (KBr) ν 3251 (NH); 1653; 1622 (C=O); 1512 (C=N); 1115; 1110 (C-N) cm^–1^.

^1^H NMR (400 MHz, DMSO–d_6_) δ (*Z*/*E* 65/35): 2.65–2.78 (m, 2H, CH_2_CO), 2.86 (s, 6H, 2CH_3_), 3.26–3.32 (m, 0.35H, CH), 3.79, 3.80 (2s, 3H, OCH_3_), 3.80–4.16 (m, 2.65H, NCH_2_+CH), 6.72 (dd, *J* = 9.3, 2.8 Hz, 2H, H_Ar_), 7.00 (t, *J* = 8.0 Hz, 2H, H_Ar_), 7.43 (d, *J* = 9.0 Hz, 2H, H_Ar_), 7.64 (d, *J* = 6.6 Hz, 2H, H_Ar_), 7.97, 8.15 (2s, 1H, N=CH), 11.43, 11.48 (2s, 1H, NH) ppm.

^13^C NMR (101 MHz, DMSO–d_6_) δ 32.92, 34.67, 34.89, 35.45 (CH_2_CO, CH), 40.34 (2CH_3_), 50.50, 50.93 (NCH_2_), 55.29 (OCH_3_), 112.39, 114.33, 121.20, 121.27, 126.68, 126.75, 128.44, 128.69, 128.85, 128.94, 143.44, 146.85, 147.60, 160.66, 160.85 168.53, 170.93, 171.12, 173.43 (C_Ar_, 2C=O) ppm

Calcd. for C_21_H_24_N_4_O_3_, %: C 66.30; H 6.36; N 14.73; found, %: C 66.25; H 6.39; N 14.69.

*N*′-(2,5-dimethoxybenzylidene)-1-(4-(dimethylamino)phenyl)-5-oxopyrrolidine-3-carbohydrazide (**5f**) 

White solid, yield 0.52 g, 64%, m. p. 165–166 °C (from 1,4-dioxane).

IR (KBr) ν 3242 (NH); 1678; 1659 (C=O); 1523 (C=N); 1111; 1078 (C-N) cm^–1^.

^1^H NMR (400 MHz, DMSO–d_6_) δ (*Z*/*E* 60/40): 2.65–2.78 (m, 2H, CH_2_CO), 2.86, 2.87 (2s, 6H, 2CH_3_), 3.17–3.32 (m, 0.4H, CH), 3.72, 3.73, 3.79, 3.80 (4s, 3H, 2OCH_3_), 3.84–4.11 (m, 2.6H, NCH_2_+CH), 6.59–6.80 (m, 2H, H_Ar_), 6.91–7.10 (m, 2H, H_Ar_), 7.28–7.37 (m, 1H, H_Ar_), 7.43 (d, *J* = 8.8 Hz, 2H, H_Ar_), 8.33, 8.53 (2s, 1H, N=CH), 11.52, 11.63 (2s, 1H, NH) ppm.

^13^C NMR (101 MHz, DMSO–d_6_) δ 32.90, 34.71, 34.97, 35.37 (CH_2_CO, CH), 40.33 (2CH_3_), 50.53, 50.86 (NCH_2_), 55.42, 56.19, 56.26 (2OCH_3_), 109.08, 109.73, 112.38, 113.25, 113.43, 116.83, 117.68, 121.23, 122.63, 122.81, 128.83, 128.94, 139.00, 142.29, 147.58, 152.10, 152.25, 153.24, 168.59, 170.89, 171.12, 173.68 (C_Ar_, 2C=O) ppm.

Calcd. for C_22_H_26_N_4_O_4_, %: C 64.37; H 6.38; N 13.65; found, %: C 64.32; H 6.36; N 13.62.

*N*′-(2,4,6-trimethoxybenzylidene)-1-(4-(dimethylamino)phenyl)-5-oxopyrrolidine-3-carbohydrazide (**5g**) 

White solid, yield 0.51 g, 57.5%, m. p. 174–175 °C (from 1,4-dioxane).

IR (KBr) ν 2962 (NH), 1667; 1647 (C=O); 1519 (C=N); 1136; 1118 (C-N) cm^–1^.

^1^H NMR (400 MHz, DMSO–d_6_) δ (*Z*/*E* 75/25): 2.65–2.76 (m, 2H, CH_2_CO), 2.86 (s, 6H, 2CH_3_), 3.23–3.31 (m, 0.25H, CH), 3.78, 3.82 (2s, 6H, OCH_3_), 3.85–4.14 (m, 2.75H, NCH_2_+CH), 4.01–4.17 (m, 1H, CH), 6.27 (s, 2H, H_Ar_), 6.72 (d, *J* = 9.0 Hz, 2H, H_Ar_), 7.38–7.46 (m, 2H, H_Ar_), 8.19, 8.34 (2s, 1H, N=CH), 11.17, 11.25 (2s, 1H, NH) ppm.

^13^C NMR (101 MHz, DMSO–d_6_) δ 34.38, 34.91, 35.40, 35.91 (CH_2_CO, CH), 40.78 (2CH_3_), 51.01, 51.44 50.95 (NCH_2_), 55.88, 56.41 (3OCH_3_), 91.58, 104.19, 104.32, 112.83, 121.63, 121.74, 129.36, 129.45, 139.24, 143.20, 148.03, 160.28, 160.35, 162.53, 162.74, 168.38, 171.48, 171.83, 173.42 (C_Ar_, 2C=O) ppm.

Calcd. for C_23_H_28_N_4_O_5_, %: C 62.71; H 6.41; N 12.72; found, %: C 62.76; H 6.38; N 12.69.

*N*′-(3,4,5-trimethoxybenzylidene)-1-(4-(dimethylamino)phenyl)-5-oxopyrrolidine-3-carbohydrazide (**5h**) 

White solid, yield 0.78 g, 89%, m. p. 160–161 °C (from 1,4-dioxane).

IR (KBr) ν 3280 (NH), 1673; 1663 (C=O); 1521 (C=N); 1182; 1129 (C-N) cm^–1^.

^1^H NMR (400 MHz, DMSO–d_6_) δ (*Z*/*E* 60/40): 2.66–2.81 (m, 2H, CH_2_CO), 2.86, 2.87 (2s, 6H, 2CH_3_), 3.69, 3.70 (2s, 3H, OCH_3_), 3.80, 3.82 (2s, 6H, 2OCH_3_), 3.84–4.16 (m, 3H, NCH_2_+CH), 6.67–6.77 (m, 2H, H_Ar_), 7.00 (s, 2H, H_Ar_), 7.39–7.48 (m, 2H, H_Ar_), 7.94, 8.14 (2s, 1H, N=CH), 11.58, 11.60 (2s, 1H, NH) ppm.

^13^C NMR (101 MHz, DMSO–d_6_) δ 32.86, 34.72, 34.90, 35.46 (CH_2_CO, CH), 40.33 (2CH_3_), 50.57, 50.95 (NCH_2_), 55.94, 60.11 (3OCH_3_), 104.10, 104.29, 112.37, 121.23, 128.82, 128.91, 129.66, 139.03, 139.20, 143.37, 146.94, 147.58, 147.61, 153.18, 168.78, 170.89, 171.12, 173.76 (C_Ar_, 2C=O) ppm.

Calcd. for C_23_H_28_N_4_O_5_, %: C 62.71; H 6.41; N 12.72; found, %: C 62.77; H 6.39; N 12.74.

1-(4-(Dimethylamino)phenyl)-*N*′-(4-nitrobenzylidene)-5-oxopyrrolidine-3-carbohydrazide (**5i**) 

White solid, yield 0.50 g, 63%, m. p. 276–277 °C (from 1,4-dioxane).

IR (KBr) ν 3267 (NH), 1672; 1645 (C=O); 1528 (C=N); 1171; 1149 (C-N) cm^–1^.

^1^H NMR (400 MHz, DMSO–d_6_) δ (*Z*/*E* 65/35): 2.62–2.82 (m, 2H, CH_2_CO), 2.86, 2.87 (2s, 6H, 2CH_3_), 3.34–3.41 (m, 0.35H, CH), 3.83–4.18 (m, 2.65H, NCH_2_+CH), 6.72 (d, *J* = 8.9 Hz, 2H, H_Ar_), 7.43 (d, *J* = 8.9 Hz, 2H, H_Ar_), 7.91–8.02 (m, 2H, H_Ar_), 8.13 (s, 0.65H, N=CH), 8.15–8.42 (m, 2H, H_Ar_+0.35H, N=CH), 11.85, 11.92 (2s, 1H, NH) ppm.

^13^C NMR (101 MHz, DMSO–d_6_) δ 32.92, 34.65, 34.98, 35.34 (CH_2_CO, CH), 40.33 (2CH_3_), 50.33, 50.77 (NCH_2_), 55.94, 60.11 (3OCH_3_), 112.38, 121.23, 121.29, 124.04, 127.87, 128.02, 128.80, 128.87, 140.44, 140.50, 141.28, 144.52, 147.62, 147.71, 147.88, 169.29, 170.80, 170.97, 174.11 (C_Ar_, 2C=O) ppm.

Calcd. for C_20_H_21_N_5_O_4_, %: C 60.75; H 5.35; N 17.71; found, %: C 60.79; H 5.32; N 17.72.

1-(4-(Dimethylamino)phenyl)-5-oxo-*N*′-(thiophen-2-ylmethylene)pyrrolidine-3-carbohydrazide (**5j**) 

Yellowish solid, yield 0.26 g, 36%, m. p. 174–175 °C (from 1,4-dioxane).

IR (KBr) ν 3279 (NH), 1660; 1597 (C=O); 1520 (C=N); 1177; 1111 (C-N) cm^–1^.

^1^H NMR (400 MHz, DMSO–d_6_) δ (*Z*/*E* 60/40): 2.64–2.76 (m, 2H, CH_2_CO), 2.86 (s, 6H, 2CH_3_), 3.26–3.32 (m, 0.5H, CH), 3.87–4.05 (m, 2.5H, NCH_2_+CH), 6.72 (d, *J* = 8.4 Hz, 2H, H_Ar_), 7.08–7.15 (m, 2H, H_Ar_), 7.38–7.47 (m, 3H, H_Ar_), 7.64 (dd, *J* = 14.1, 5.1 Hz, 1H, H_Ar_), 8.20, 8.43 (2s, 1H, N=CH), 11.54, 11.57 (2s, 1H, NH) ppm.

^13^C NMR (101 MHz, DMSO–d_6_) δ 33.12, 34.56, 34.91, 35.40 (CH_2_CO, CH), 40.34 (2CH_3_), 50.43, 50.88 (NCH_2_), 112.40, 121.22, 121,27, 127.96, 128.30, 128.42, 128.75, 128.91, 129.29, 129.35, 129.44, 131,47, 134.28, 138.41, 138.54, 138.98, 142.14, 155.83, 168.64, 170.87, 171.01 (C_Ar_, 2C=O) ppm.

Calcd. for C_18_H_20_N_4_O_2_S, %: C 60.65; H 5.66; N 15.72; found, %: C 60.62; H 5.64; N 15.74.

1-(4-(Dimethylamino)phenyl)-*N*′-((5-nitrothiophen-2-yl)methylene)-5-oxopyrrolidine-3-carbohydrazide (**5k**)

Brown solid, yield 0.61 g, 76%, m. p. 266–267 °C (from 1,4-dioxane).

IR (KBr) ν 3214 (NH), 1681; 1646 (C=O); 1528 (C=N); 1173; 1111 (C-N) cm^–1^.

^1^H NMR (400 MHz, DMSO–d_6_) δ 2.64–2.82 (m, 2H, CH_2_CO), 2.86 (s, 6H, 2CH_3_), 3.34–3.38 (m, 0.4H, CH), 3.87–4.05 (m, 2.6H, NCH_2_+CH), 6.72 (d, *J* = 8.4 Hz, 2H, H_Ar_), 7.42 (d, *J* = 8.4 Hz, 2H, H_Ar_), 7.53, 7.56 (2d, *J* = 4.3 Hz, 1H, H_Ar_), 8.06–8.14 (m, 1H, H_Ar_), 8.20, 8.48 (2s, 1H, N=CH), 11.97 (s, 1H, NH) ppm.

^13^C NMR (101 MHz, DMSO–d_6_) δ 32.96, 34.61, 35.01, 35.27 (CH, CH_2_CO), 40.33 (2CH_3_), 50.31, 50.70 (NCH_2_), 112.38, 121.30, 128.84, 129.20, 129.76, 130.64, 136.84, 140.54, 146.60, 147.64, 150.54, 150.86, 169.34, 170.74, 170.89, 173.96 (C_Ar_, 2C=O) ppm.

Calcd. for C_18_H_19_N_5_O_4_S, %: C 53.86; H 4.77; N 17.45; found, %: C 53.82; H 4.73; N 17.43.

1-(4-(Dimethylamino)phenyl)-*N*′-(naphthalen-1-ylmethylene)-5-oxopyrrolidine-3-carbohydrazide (**5l**)

Yellowish solid, yield 0.40 g, 50%, m. p. 180–181 °C (from 1,4-dioxane).

IR (KBr) ν 3203 (NH), 1674; 1660 (C=O); 1521 (C=N); 1166; 1115 (C-N) cm^–1^.

^1^H NMR (400 MHz, DMSO–d_6_) δ (*Z*/*E* 60/40): 2.72–2.84 (m, 2H, CH_2_CO), 2.85, 2.87 (2s, 6H, 2CH_3_), 3.36–3.42 (m, 0.4H, CH), 3.93–4.16 (m, 2.6H, NCH_2_+CH), 6.73 (t, *J* = 8.5 Hz, 2H, H_Ar_), 7.45 (dd, *J* = 9.1, 4.1 Hz, 1H, H_Ar_), 7.57–7.68 (m, 3H, H_Ar_), 7.88–8.04 (m, 3H, H_Ar_), 8.57 (d, *J* = 8.5 Hz, 0.6H, H_Ar_), 8.75, 8.83 (2s, 1H, N=CH), 8.87 (d, *J* = 8.5 Hz, 0.4H, H_Ar_), 11.61, 11.74 (2s, 1H, NH) ppm.

^13^C NMR (101 MHz, DMSO–d_6_) δ 33.01,34.72, 35.00, 36.45 (CH_2_CO, CH), 40.33 (2CH_3_), 50.48, 50.92 (NCH_2_), 123.40, 128.33, 125.63, 126.28, 126.31, 126.87, 127.35, 127.37, 128.21, 128.88, 128.91, 129.34, 129.37, 130.12, 130.35, 130.63, 133.49, 133.54 143.18, 147.05, 147.59, 168.82, 170.91, 171.08, 173.65 (C_Ar_, 2C=O) ppm.

Calcd. for C_24_H_24_N_4_O_2_, %: C 71.98; H 6.04; N 13.99; found, %: C 71.91; H 6.07; N 13.96.

General method of the preparation of hydrazones **6a**,**b**

A mixture of hydrazide **4** (0.52 g 2 mmol), acetone or methylethylketone (10 mL), and 5 drops of acetic acid was heated at reflux for 18 h and then cooled, half of the volatile fraction was evaporated under reduced pressure, and the formed crystalline solid was filtered off and washed with acetone to give the title compound **6a** or **6b**.

1-(4-(Dimethylamino)phenyl)-5-oxo-*N*′-(propan-2-ylidene)pyrrolidine-3-carbohydrazide (**6a**) 

White solid, yield 0.45 g, 74%, m. p. 206–208 °C (from acetone).

IR (KBr): ν 3300 (NH), 1671; 1651 (C=O); 1522 (C=N); 1128; 1116 (C-N) cm^–1^.

^1^H NMR (400 MHz, DMSO–d_6_) δ (*Z*/*E* 55/45): 1.86, 1.88, 1.93 (3s, 6H, 2CH_3_), 2.59–2.71 (m, 2H, CH_2_CO), 2.87 (s, 6H, 2CH_3_), 3.37–3.44 (m, 0.6H, CH), 3.77–4.00 (m, 2.4H, NCH_2_+CH), 6.75 (d, *J* = 8.5 Hz, 2H, H_Ar_), 7.43 (dd, *J* = 9.1, 3.9 Hz, 2H, H_Ar_), 10.21, 10.29 (2s, 1H, NH) ppm.

Calcd. for C_16_H_22_N_4_O_2_, %: C 63.55; H 7.33; N 18.53; found, %: C 63.52; H 7.35; N 18.51.

*N*′-(butan-2-ylidene)-1-(4-(dimethylamino)phenyl)-5-oxopyrrolidine-3-carbohydrazide (**6b**)

White solid, yield 0.20 g, 32%, m. p. 124–125 °C (from acetone).

IR (KBr): ν 3315 (NH), 1691; 1672 (C=O); 1522 (C=N); 1125; 1112 (C-N) cm^–1^.

^1^H NMR (400 MHz, DMSO–d_6_) δ (mixture of *Z*, *E*, s-*Z*, and s-*E* isomers): 0.98, 1.03 (2t, *J* = 7.3 Hz, 3H, CH_3_), 1.85, 1.87, 1.91 (3s, 6H, 2CH_3_), 2.20–2.34 (m, 2H, CH_2_), 2.58–2.72 (m, 2H, CH_2_CO), 2.86 (s, 6H, 2CH_3_), 3.37–3.45 (m, 0.4H, CH), 3.78–4.01 (m, 2.6H, NCH_2_+CH), 6.72 (d, *J* = 8.5 Hz, 2H, H_Ar_), 7.42 (dd, *J* = 9.0, 4.3 Hz, 2H, H_Ar_), 10.17, 10.24, 10.32, 10.39 (4s, 1H, NH) ppm.

^13^C NMR (101 MHz, DMSO–d_6_) δ 10.36, 10.79, 16.06, 16.10, 22.17, 22.58, 22.98, 31.44, 31.54, 33.26, 34.48, 34.68, 35.60 (CH_3_CH_2_, CH_3_, CH_2_CO, CH), 40.34 (2CH_3_), 50.51, 51.22 (NCH_2_), 112.39, 121.17, 121.21, 128.95, 147.58, 154.44, 159.64 (C_Ar_), 168.77, 171.09, 171.20, 173.86 (2C=O) ppm.

Calcd. for C_17_H_24_N_4_O_2_, %: C 64.53; H 7.65; N 17.71; found, %: C 64.56; H 7.62; N 17.72.

*N*′-(1-(4-aminophenyl)ethylidene)-1-(4-(dimethylamino)phenyl)-5-oxopyrrolidine-3-carbohydrazide (**6c**)

A mixture of hydrazide **4** (0.35 g, 1.3 mmol), 4′-aminoacetophenone (0.36 g, 2.7 mmol), and acetic acid (3 mL) in 1,4-dioxane (55 mL) was heated at reflux for 5 h, and then half of the dioxane was evaporated under reduced pressure, the residue was diluted with propan-2-ol (8 mL), and the mixture was left in the refrigerator overnight. The formed solid was filtered off and washed with 1,4-dioxane and diethyl ether to give the title compound **6c**.

White solid, yield 0.38 g, 78%, m. p. 146–147 °C (from propan-2-ol).

IR (KBr): ν 3339 (NH), 1671; 1632 (C=O); 1520 (C=N); 1183; 1118 (C-N) cm^–1^.

^1^H NMR (400 MHz, DMSO–d_6_) δ (*Z*/*E* 65/35): 2.16, 2.18 (2s, 3H, CH_3_), 2.66–2.79 (m, 2H, CH_2_CO), 2.86 (s, 6H, 2CH_3_), 3.83–4.13 (m, 3H, NCH_2_+CH), 5.43, 5.46 (2s, 2H, NH_2_), 6.56 (d, *J* = 8.2 Hz, 2H, H_Ar_), 6.67–6.77 (m, 2H, H_Ar_), 7.43 (d, *J* = 9.2 Hz, 2H, H_Ar_), 7.51 (d, *J* = 8.2 Hz, 2H, H_Ar_), 10.34, 10.47 (2s, 1H, NH) ppm.

^13^C NMR (101 MHz, DMSO–d_6_) δ 13.24, 13.81 (CH_3_), 30.70, 33.19, 34.65, 35.69 (CH_2_CO, CH), 40.34 (2CH_3_), 50.65, 51.31 (NCH_2_), 112.39, 113.12, 113.26, 121.19, 125.06, 125.23, 127.23, 127.59, 128.987, 130.56, 147.56, 147.58, 128.93, 150.01, 150.25, 153.60, 168.80 (C_Ar_), 171.13, 171,24, 174.01 (2C=O) ppm.

Calcd. for C_21_H_25_N_5_O_2_, %: C 66.47; H 6.64; N 18.46; found, %: C 66.44; H 6.66; N 18.43.

*N*-(2,5-dimethyl-1*H*-pyrrol-1-yl)-1-(4-(dimethylamino)phenyl)-5-oxopyrrolidine-3-carboxamide (**7**) 

A mixture of hydrazide **4** (0.52 g, 2 mmol), hexane-2,5-dione (0.57 mL, 5 mmol) propan-2-ol (50 mL), and glacial acetic acid (5 drops) was refluxed for 18 h and then cooled. The formed precipitate was filtered off and washed with propan-2-ol, diethyl ether to give the title compound **7**.

White solid, yield 0.44 g, 64%, m. p. 163–164 °C (from propan-2-ol).

IR (KBr) ν 3231 (NH), 1688; 1658 (C=O); 1520 (C=N); 1221; 1198; 1136; 1112 (C-N) cm^–1^.

^1^H NMR (400 MHz, DMSO–d_6_) δ 1.94, 1.98, 1.99 (3s, 6H, 2CH_3_), 2.64–2.69 (m, 1H, CH_2_CO), 2.77–2.82 (m, 1H, CH_2_CO), 2.87 (s, 6H, 2CH_3_), 3.39–3.46 (m, 1H, CH), 3.85–3.92 (m, 1H, NCH_2_), 4.01–4.09 (m, 1H, NCH_2_), 5.48, 5.64 (2s, 2H, 2CH_Pyrr_), 6.73 (d, *J* = 8.8 Hz, 2H, H_Ar_), 7.42 (d, *J* = 9.1 Hz, 2H, H_Ar_), 10.78, 10.88 (2s, 1H, NH) ppm.

^13^C NMR (101 MHz, DMSO–d_6_) δ 8.77, 10.94 (2CH_3_), 34.13, 35.35, 40.32, 50.84 (CH, CH_2_CO, N(CH_3_)_2_, NCH_2_), 103.06 (C_Pyrr_), 112.38, 121.37, 128.71, 147.68 C_Ar_, C_Pyrr_), 170.68, 172.05 (2C=O) ppm.

Calcd. for C_19_H_24_N_4_O_2_, %: C 67.04; H 7.11; N 16.46; found, %: C 67.08; H 7.13; N 16.48.

1-(1-(4-(Dimethylamino)phenyl)-5-oxopyrrolidine-3-carboxamido)-5-oxopyrrolidine-3-carboxylic acid (**8**)

A mixture of hydrazide **4** (0.49 g, 1.9 mmol), itaconic acid (0.38 g, 2.9 mmol), and water (10 mL) was heated at reflux for 24 h and then cooled down, and a part of the water was evaporated under reduced pressure. The resin residue was dissolved in acetone, and the solution was diluted with hexane to isolate a crystalline product **8**.

Light brown solid, yield 0.15 g, 21%, m. p. 186–187 °C (from propan-2-ol).

IR (KBr) ν 3277 (NH), 1729; 1670 (C=O); 1520 (C=N) cm^–1^.

^1^H NMR (400 MHz, DMSO–d_6_) δ 2.51–2.61 (m, 3H, CH_2_CO), 2.68–2.77 (m, 1H, CH_2_CO), 2.86 (s, 6H, 2CH_3_), 3.24–3.33 (m, 2H, 2CH), 3.57–3.63 (m, 1H, NCH_2_), 3.66–3.72 (m, 1H, NCH_2_), 3.77–3.83 (m, 1H, NCH_2_), 3.91–4.02 (m, 1H, NCH_2_), 6.72 (d, *J* = 8.6 Hz, 2H, H_Ar_), 7.40 (d, *J* = 8.6 Hz, 2H, H_Ar_), 10.34 (s, 1H, NH), 12.77 (br. s, 1H, OH) ppm.

^13^C NMR (101 MHz, DMSO–d_6_) δ 31.27, 33.74, 34.12, 35.15 (CH, CH_2_CO), 40.35 (N(CH_3_)_2_), 49.65, 50.73 (NCH_2_), 112.39, 121.31, 128.74, 147.67 (C_Ar_), 170.72, 170.90, 171.54, 174.04 (4C=O) ppm.

Calcd. for C_18_H_22_N_4_O_5_, %: C 57.75; H 5.92; N 14.96; found, %: C 57.78; H 5.93; N 14.99.

1-(4-(Dimethylamino)phenyl)-5-oxo-*N*′-(2-oxoindolin-3-ylidene)pyrrolidine-3-carbohydrazide (**9**)

To a mixture of hydrazide **4** (0.29 g, 1.1 mmol), isatin (0.25 g, 1.7 mmol), and methanol (80 mL), glacial acetic acid (3 drops) was added, and the mixture was refluxed for 4 h. After completion of the reaction, the formed crystalline solid was filtered off, washed with methanol, dried to give the title compound **9**.

Yellow solid, yield 0.18 g, 42%, m. p. 208–209 °C (from propan-2-ol:DMF, 10:1).

IR (KBr) ν 3386; 3238 (NH), 1732; 1716; 1695 (C=O); 1520 (C-N) cm^–1^.

^1^H NMR (400 MHz, DMSO–d_6_) δ 2.72–2.83 (m, 2H, CH_2_CO), 2.87 (s, 6H, 2CH_3_), 3.74–4.19 (m, 3H, NCH_2_+CH), 6.72 (d, *J* = 8.6 Hz, 2H, H_Ar_), 6.90 (d, *J* = 7.8 Hz, 1H, H_Ar_), 7.05 (br. s, 1H, H_Ar_), 7.32–7.41 (m, 1H, H_Ar_), 7.42 (d, *J* = 8.6 Hz, 2H, H_Ar_), 10.82 (s, 1H, NH_Isat_), 11.35 (s, 1H, NH) ppm.

^13^C NMR (101 MHz, DMSO–d_6_) δ 33.54, 35.54 (CH, CH_2_CO), 40.34 (N(CH_3_)_2_), 50.58 (NCH_2_), 110.62, 112.38, 115.20, 121.36, 121,68, 126.17, 128.78, 132.58, 136.48, 143.85, 147.66 (C_Ar_), 159.28, 164.58, 170.81 (C=O) ppm.

Calcd. for C_21_H_21_N_5_O_3_, %: C 64.44; H 5.41; N 17.89; found, %: C 64.41; H 5.42; N 17.87.

1-(4-(Dimethylamino)phenyl)-4-(5-thioxo-4,5-dihydro-1,3,4-oxadiazol-2-yl)pyrrolidin-2-one (**10**) 

To a solution of potassium hydroxide (0.34 g, 6.05 mmol) in methanol, carbon disulfide (0.42 g, 5.5 mmol) was added dropwise, and the mixture was stirred for 15 min at room temperature. Then, the solution of acid hydrazide **4** (0.29 g, 1.1 mmol) in methanol was added, and the mixture was heated at reflux for 7 h. After completion of the reaction, the volatile fraction was evaporated under reduced pressure, and the residue was diluted with water and acidified with acetic acid to pH 6 to give the title compound **10**.

White solid, yield 0.19 g, 56%, m. p. 206–207 °C (from methanol).

IR (KBr) ν 3420 (NH), 1655 (C=O); 1522 (C=N); 1230 (C=S) cm^–1^.

^1^H NMR (400 MHz, DMSO–d_6_) δ 2.74–2.81 (m, 1H, CH_2_CO), 2.86 (s, 6H, 2CH_3_), 2.89–2.95 (m, 1H, CH_2_CO), 3.88–3.96 (m, 1H, CH), 3.96–4.16 (m, 2H, NCH_2_), 6.72 (d, *J* = 8.8 Hz, 2H, H_Ar_), 7.40 (d, *J* = 8.8 Hz, 2H, H_Ar_), 13.89 (br. s, 1H, NH) ppm.

^13^C NMR (101 MHz, DMSO–d_6_) δ 27.98, 34.92 (CH, CH_2_CO), 40.33 (N(CH_3_)_2_), 50.31 (NCH_2_), 112.35, 121.50, 128.45, 147.78, 164.06 (C_Ar_), 170.05 (C=O), 178.0 (C=S) ppm.

Calcd. for C_14_H_16_N_4_O_2_S, %: C 55.25; H 5.30; N 18.41; found, %: C 55.22; H 5.31; N 18.39.

1-(4-(Dimethylamino)phenyl)-4-(5,6-diphenyl-1,2,4-triazin-3-yl)pyrrolidin-2-one (**11**)

A mixture of hydrazide **4** (0.5 g, 1.9 mmol), benzyl (0.4 g, 1.9 mmol), ammonium acetate (1.46 g, 19 mmol), and glacial acetic acid (8 mL) was heated at reflux for 24 h and then cooled down, diluted with water, and left in the refrigerator overnight. Afterward, the aqueous solution was decanted, and then the resin residue was washed 3-fold with water, poured with water, and acidified with HCl to pH 1. The mixture was refluxed for 5 min, cooled, and neutralized with sodium acetate. The crystalline solid was filtered off, washed with water, and dried to give the title compound **11**.

Yellowish solid, yield 0.21 g, 43%, m. p. 64–65 °C (from propan-2-ol).

IR (KBr) ν 1691 (C=O); 1520 (C=N) cm^–1^.

^1^H NMR (400 MHz, DMSO–d_6_) δ 2.86 (s, 6H, 2CH_3_), 3.05–3.14 (m, 2H, CH_2_CO), 4.19–4.36 (m, 3H, NCH_2_+CH), 6.73 (d, *J* = 8.6 Hz, 2H, H_Ar_), 7.37–7.53 (m, 12H, H_Ar_) ppm.

^13^C NMR (101 MHz, DMSO–d_6_) δ 37.00, 37.14 (CH, CH_2_CO), 40.35 (N(CH_3_)_2_), 52.60 (NCH_2_), 112.39, 121.34, 127.52, 128.40, 128.51, 128.64, 128.70, 128.87, 128.92, 128.97, 129.14, 129.46, 129.78, 129.92, 130.03, 130.18, 130.65, 135.36, 135.44, 147.63, 155.91, 156.09, 167.03, 171.16 (C_Ar_, C=O) ppm.

Calcd. for C_27_H_25_N_5_O, %: C 74.46; H 5.79; N 16.08; found, %: C 74.50; H 5.77; N 16.05.

### 3.2. Pharmacology

#### 3.2.1. Cell Culturing

The human triple-negative breast cancer MDA-MB-231 cell line and human pancreatic carcinoma cell line Panc-1 were obtained from the American Type Culture Collection (ATCC, Manassas, VA, USA). Human foreskin fibroblasts (HF) CRL-4001 were originally obtained from ATCC and kindly provided by Prof. Helder Santos (University of Helsinki, Helsinki, Finland). The cells were grown in Dulbecco’s Modified Eagle’s medium supplemented with GlutaMAX (Gibco, Carlsbad, CA, USA) and 10,000 U/mL penicillin, 10 mg/mL streptomycin (Gibco), and 10% fetal bovine serum (Gibco) (further referred as to cell culture medium). Cells were cultured in a humidified atmosphere with 5% CO_2_ at 37 °C.

#### 3.2.2. Cell Viability Assay

The effect of compounds on cell viability was tested by MTT assay, as described elsewhere [53]. Briefly, MDA-MB-231 and Panc-1 cells were plated into 96-well F-bottom plates (Corning, Corning, NY, USA) in triplicates at a concentration of 4 × 10^3^ cells/well in 100 μL of cell culture medium. After 24 h of incubation, 100 μL of 200 μM compound solution in a cell culture medium was added, thus making the final concentration of 100 μM. The medium without compounds with only 0.5% DMSO was used as a negative control, and the wells without cells were used as a positive control. After 72 h of incubation, the medium was removed, fresh cell culture medium containing 0.5 mg/mL of 3-(4,5-dimethylthiazol-2-yl)-2,5-diphenyltetrazolium bromide (MTT; Sigma-Aldrich Co., St. Louis, MO, USA) was added, and the plates were incubated for 4 h in a humidified atmosphere with 5% CO_2_ at 37 °C. Then, the medium was aspirated, and the formed formazan crystals were dissolved in 100 μL of dimethylsulfoxide (DMSO; Sigma-Aldrich Co., St. Louis, MO, USA). The absorbance was measured at 570 and 630 nm using a multi-detection microplate reader. The effect on cell viability was calculated using the formula:

$$Relative\;cell\;viability\;\left(\%\right)=\frac{A-A_{0}}{A_{NC}-A_{0}}$$

where

A—mean absorbance of the tested compound;

A_0_—mean absorbance of the blank (no cells, positive control);

A_NC_—mean of absorbance of negative control (only cells, no treatment).

To establish the half-maximum effective concentrations (EC_50_) that indicated the concentrations of compounds at which the cell viability is reduced by 50%, the same MTT assay was used. Serial dilutions of the most active compounds from 100 µM to 3.125 µM (100 µM, 50 µM, 25 µM, 12.5 µM, 6.25 µM, and 3.125 µM) were made in a cell culture medium and added to the cells in triplicates. EC_50_ values were calculated using the Hill equation.

#### 3.2.3. Clonogenic Assay

The compound effect on cell colony formation and growth was evaluated by the clonogenic assay as described elsewhere [55]. Briefly, the human triple-negative breast cancer MDA-MB-231 and pancreatic carcinoma Panc-1 cells were seeded into 12-well plates in triplicates at a density of 2 × 10^2^ cells/well and incubated overnight in a humidified atmosphere containing 5% CO_2_ at 37 °C. Then, compounds **3c**, **3d**, **5k**, and **5l** were added to a final concentration of 1 µM and 2 µM. The medium containing 0.5% DMSO served as a negative control. Then, the plates were incubated in a humidified atmosphere with 5% CO_2_ at 37 °C for the next 7 days. After incubation, the media from the cells were removed and the cells were washed with PBS. Then, the cells were fixed in a 4% formaldehyde solution (Thermo Scientific, Waltham, MA, USA), washed with PBS twice, and stained with 0.1% Crystal Violet (Sigma-Aldrich Co.) solution for 20 min. After the stain was removed, the stained colonies were carefully washed three times with sterile water and dried overnight. Then, the colonies were imaged with the SYNGENE G:BOX gel doc system, using Gen Sys software version 1.5.5.0, followed by quantification with Gene tools software version 4.3.8.

#### 3.2.4. Wound Healing Assay

The cells’ ability to migrate was evaluated using a ‘wound-healing’ assay, as described elsewhere [53]. Briefly, MDA-MB-231 and Panc-1 cells were seeded in 24-well plates at a density of 5 × 10^4^ cells/well and incubated for 48 h in a cell culture medium in a humidified atmosphere with 5% CO_2_ at 37 °C until they reached appr. 90% of confluency. Then, a scratch was made in the center of each well using a 100 μL pipette tip. The medium was removed and washed carefully with PBS. Then, 500 μL of fresh medium containing 1 µM or 2 µM of tested compounds **3c**, **3d**, **5k**, and **5l** was added. The cell culture medium containing 0.1% DMSO was used as a negative control. The plates were incubated in a humidified atmosphere containing 5% CO_2_ at 37 °C for up to 72 h.

Photos of ‘wounds’ were taken at intervals of 0 h, 24 h, 48 h, and 72 h under phase contrast microscopy at a 4× magnification. The ‘wound’ area was analyzed using the ImageJ program, version 1.53o (National Institute of Health, Bethesda, MD, USA).

#### 3.2.5. Testing in 3D Cultures

Cell spheroids were formed using magnetic 3D Bioprinting methods, as described elsewhere [53]. Briefly, the human triple-negative breast cancer MDA-MB-231 cells, the human pancreatic Panc-1 cells, and human fibroblasts were grown in a 6-well plate until they reached 70% of confluence. Then, 25 µL of Nanoshuttle (n3D Biosciences, Inc., Allentown, PA, USA) was added to the cells, and the incubation was continued for 8 h more. Then, the cells were trypsinized and cell suspensions were prepared. Cells were seeded into an ultra-low attachment 96-well plate (Corning) at a volume of 100 µL containing (1.5 × 10^3^ cancer cells and 1.5 × 10^3^ human fibroblasts/well). The plate was put on the special magnetic drive (n3D Biosciences, Inc., Allentown, PA, USA) and kept in the incubator for 48 h while the spheroids were formed. Then, the medium was replaced with fresh medium containing 10 µM of tested compounds.

The photos of spheroids were taken at the beginning of the experiment (Day 0) and every two days using an Olympus IX73 inverted microscope (OLYMPUS Corporation, Tokyo, Japan). The size of spheroids was evaluated using ImageJ (National Institute of Health, Bethesda, MD, USA) and Microsoft Office Excel software, v. 2303 (Redmond, Washington, DC, USA).

At the end of the experiment (on Day 8), 10 µL of WST-1 reagent (Sigma-Aldrich Co, St. Louis, MO, USA) was added to each well. After 12 h of incubation, 70 µL of solution from each well was transferred to the new 96-well plate, and the absorbance was measured at 460 and 530 nm using a multi-detection microplate reader. Spheroid cell viability was calculated using a formula provided in Section 3.2.2.

### 3.3. Statistical Analysis

All biological experiments were repeated three times, and the means and standard deviations were calculated. The data were processed using Microsoft Office Excel 2016 software (Microsoft Corporation, Redmond, WA, USA). Statistical analysis was performed using a Student’s *t*-test. For the comparison of three or more groups, one-way ANOVA was used. The level of significance was set as *p* < 0.05.

## 4. Conclusions

In summary, a series of 4-((dimethylamino)phenyl)pyrrolidin-2-ones bearing hydrazide, hydrazone, and heterocyclic moieties were synthesized and tested for the antitumor activity against triple-negative breast cancer and pancreatic cancer cell lines for their effect on cell viability, colony formation, cell migration, and activity in cell spheroids.

Pyrrolidinone derivatives **3c** and **3d**, with the incorporated 5-chloro and 5-methylbenzimidazole fragments, hydrazone **5k** bearing a 5-nitrothien-2-yl substitution, and hydrazone **5l** with a naphth-1-yl fragment in the structure significantly decreased the viability of triple-negative breast cancer and ductal pancreatic carcinoma cell lines. In our study, the triple-negative breast cancer cell line MDA-MB-231 was more sensitive to treatment with the synthesized compounds, among which compounds **3d** and **5k** were the most active. In the pancreatic cell line, **3d** showed the highest activity in 3D cell culture; however, **5k** was the most cytotoxic and exhibited the highest effect on cell colony formation and growth. Four compounds (**3c**, **3d**, **5k**, and **5l**) were the most active against both cell lines.

Considering the different activities in the biological assays, the selected pyrrolidinone derivatives could be further tested to better understand the structure–activity relationship and their mechanism of action.

## Data Availability

The data are contained within this article and the Supplementary Materials.

## Supplementary Materials

The following supporting information can be downloaded at: https://www.mdpi.com/article/10.3390/ijms25031834/s1.
