# Supplementary material for: The Effect of 4-(Dimethylamino)phenyl-5-oxopyrrolidines on Breast and Pancreatic Cancer Cell Colony Formation, Migration, and Growth of Tumor Spheroids
Source: Int J Mol Sci. 2024 Feb 2;25(3):1834. doi: 10.3390/ijms25031834 (PMC10855844; doi:10.3390/ijms25031834)
Supplement: Supplementary file 1 [file ijms-25-01834-s001.zip › ijms-2834672-supplementary.pdf]

Supplementary Material

The Effect of 4-(Dimethylamino)phenyl-5-oxopyrrolidines on Breast and Pancreatic Cancer Cell Colony Formation, Migration, and Growth of Tumor Spheroids

Karolina Kairytė [1], Rita Vaickelionienė [1], Birutė Grybaitė [1], Kazimieras Anusevičius [1], Vytautas Mickevičius [1] and Vilma Petrikaitė [2,3,4,*]

[1] Department of Organic Chemistry, Kaunas University of Technology, Radvilėnų Rd. 19, LT-50254 Kaunas, Lithuania; karolina.kairyte@ktu.lt (K.K.); rita.vaickelioniene@ktu.lt (R.V.); birute.grybaite@ktu.lt (B.G.); kazimieras.anusevicius@ktu.lt (K.A.); vytautas.mickevicius@ktu.lt (V.M.)

[2] Institute of Biotechnology, Life Sciences Center, Vilnius University, Saulėtekio Al. 7, LT-10257 Vilnius, Lithuania

[3] Faculty of Medicine, Lithuanian University of Health Sciences, A. Mickevičiaus 9, LT-44307 Kaunas, Lithuania

[4] Laboratory of Drug Targets Histopathology, Institute of Cardiology, Lithuanian University of Health Sciences, Sukilėlių Pr. 13, LT-50162 Kaunas, Lithuania

* Correspondence: vilma.petrikaite@lsmuni.lt or vilma.petrikaite@bti.vu.lt; Tel.: +370-68629383

4-(1*H*-benzo[d]imidazol-2-yl)-1-(4-(dimethylamino)phenyl)pyrrolidin-2-one (3a)

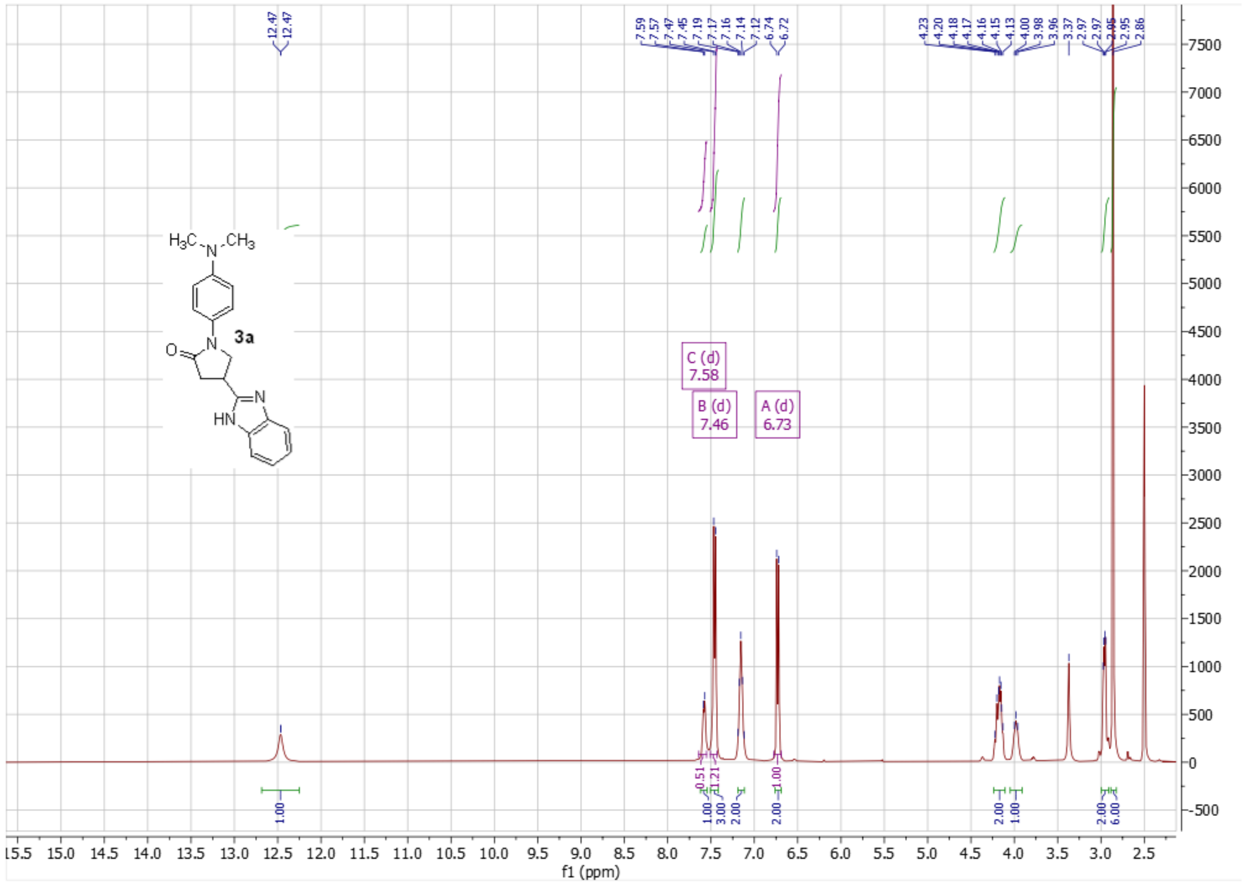

Figure S1. ^{1}H NMR spectrum of compound **3a.**

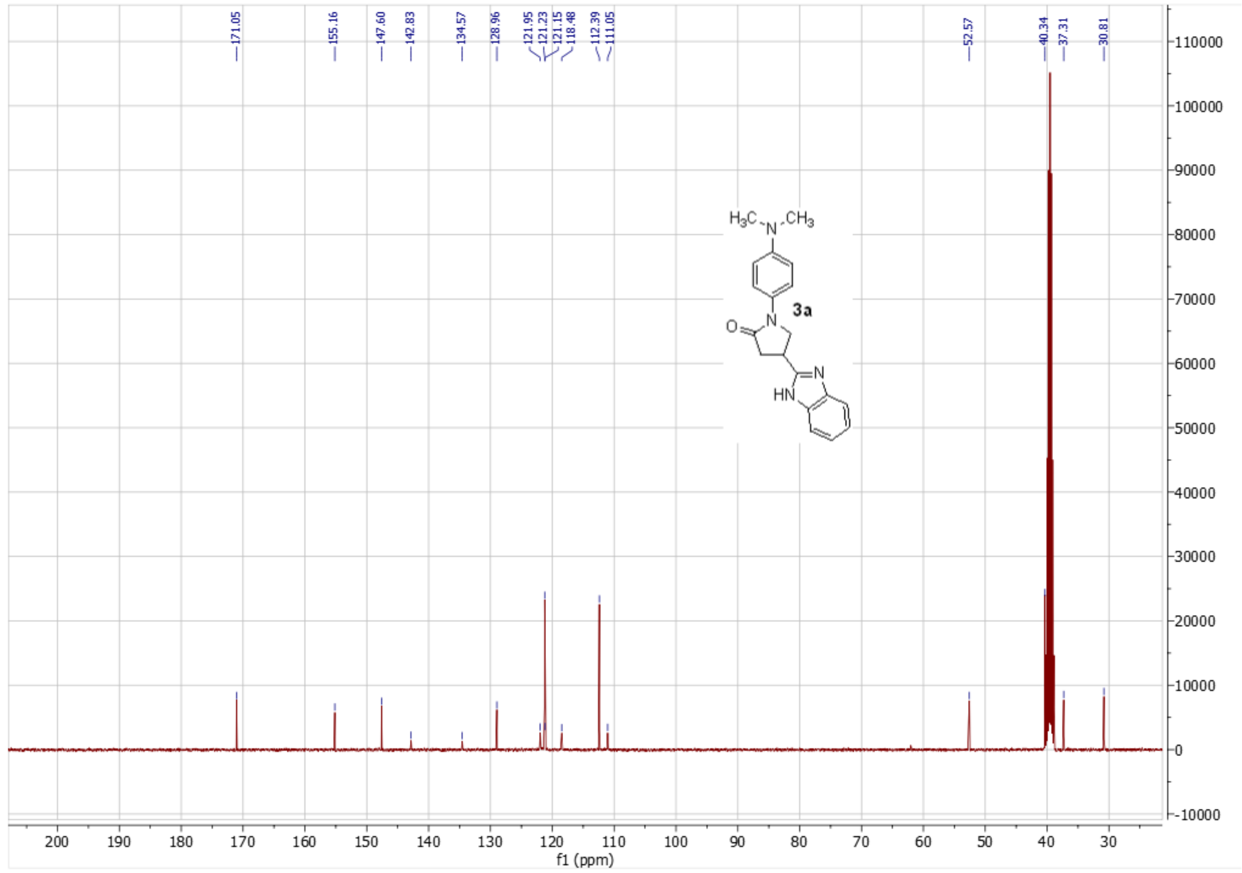

Figure S2. ^{13}C NMR spectrum of compound **3a.**

1-(4-(Dimethylamino)phenyl)-4-(5-fluoro-1*H*-benzo[d]imidazol-2-yl)pyrrolidin-2-one (3b)

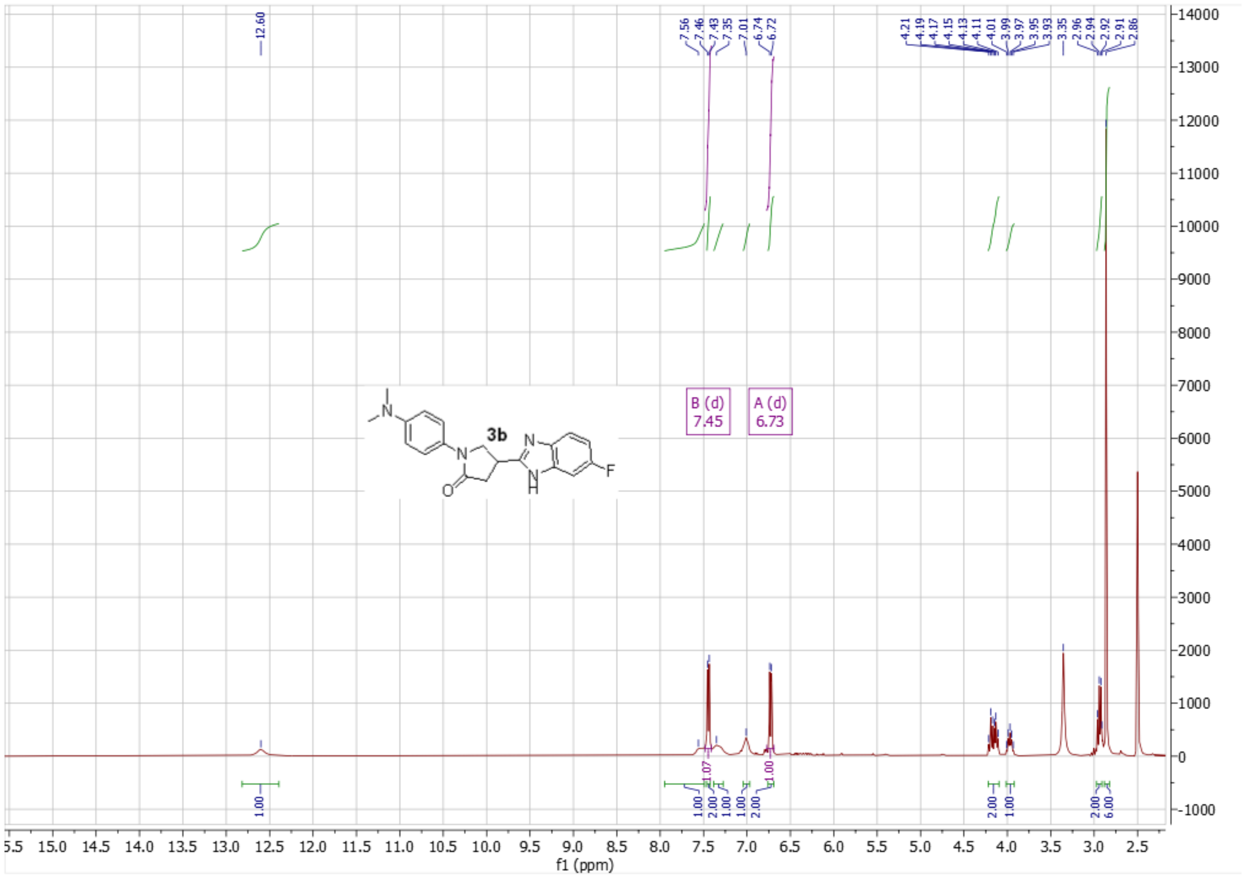

Figure S3. ^{1}H NMR spectrum of compound **3b.**

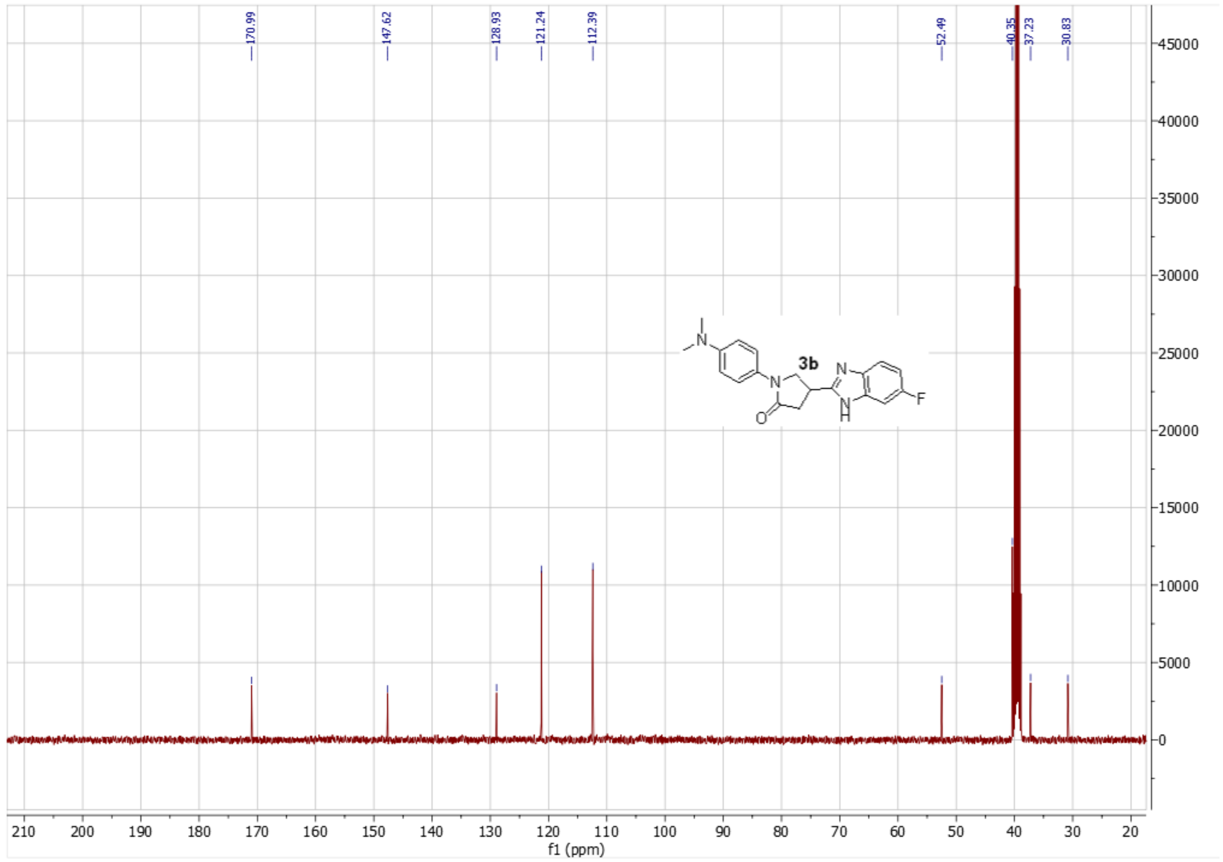

Figure S4. ^{13}C NMR spectrum of compound **3b.**

1-(4-(Dimethylamino)phenyl)-4-(5-chloro-1H-benzo[d]imidazol-2-yl)pyrrolidin-2-one (3c)

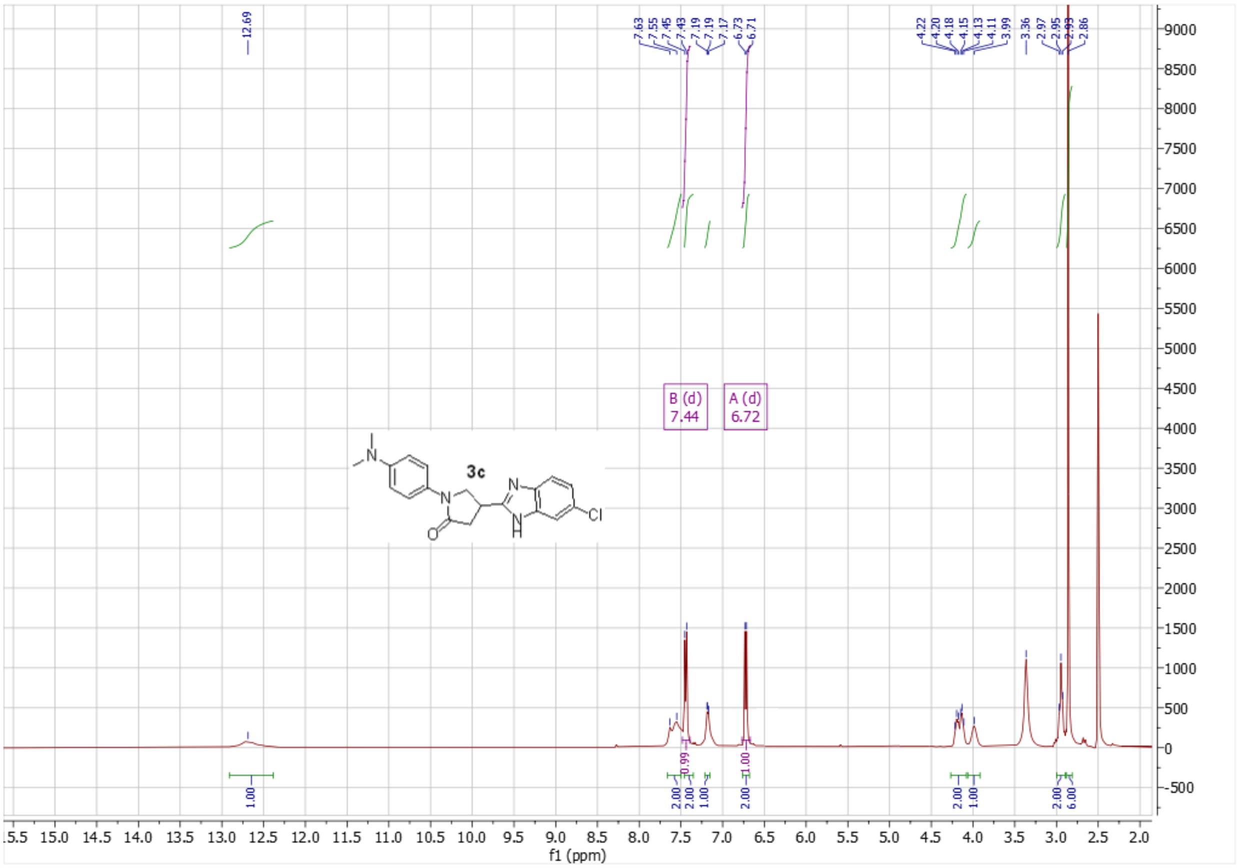

Figure S5. ^{1}H NMR spectrum of compound **3c.**

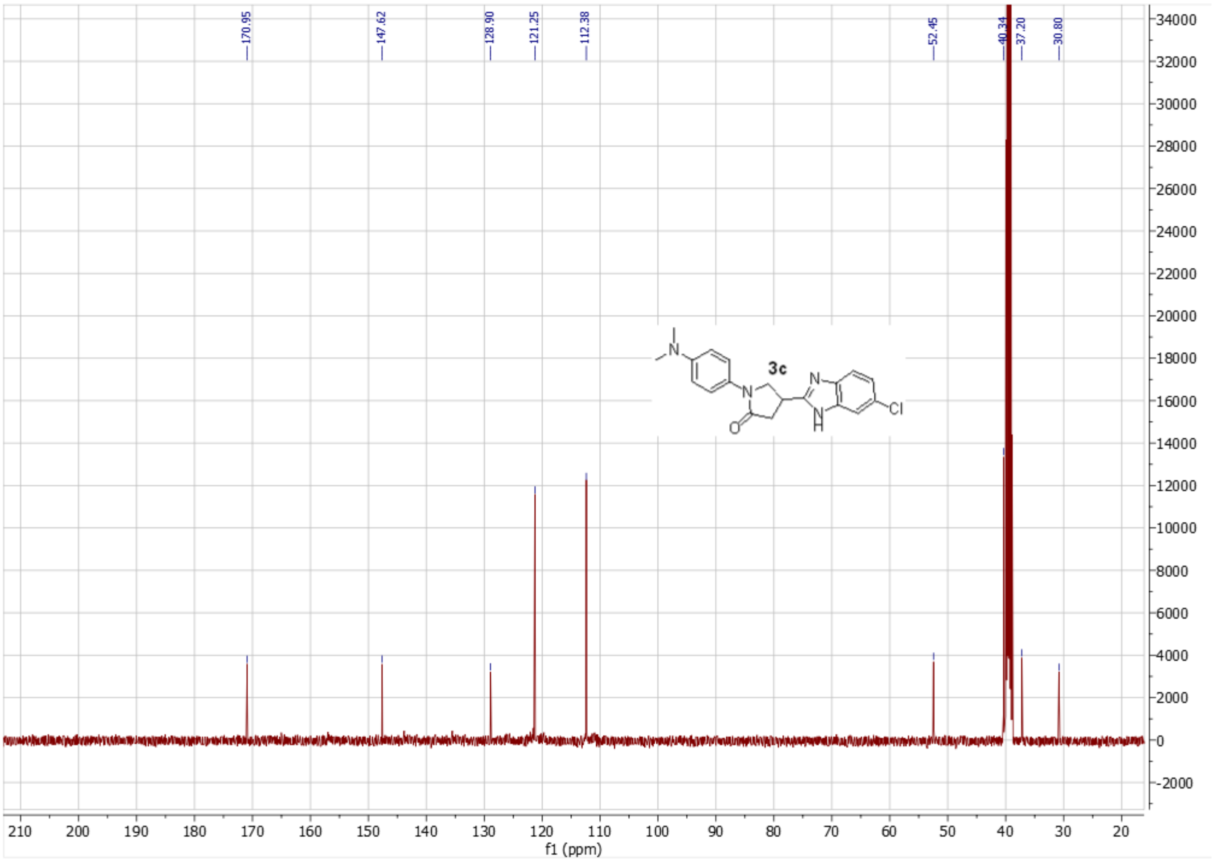

Figure S6. ^{13}C NMR spectrum of compound **3c.**

1-(4-(Dimethylamino)phenyl)-4-(5-methyl-1*H*-benzo[d]imidazol-2-yl)pyrrolidin-2-one (3d)

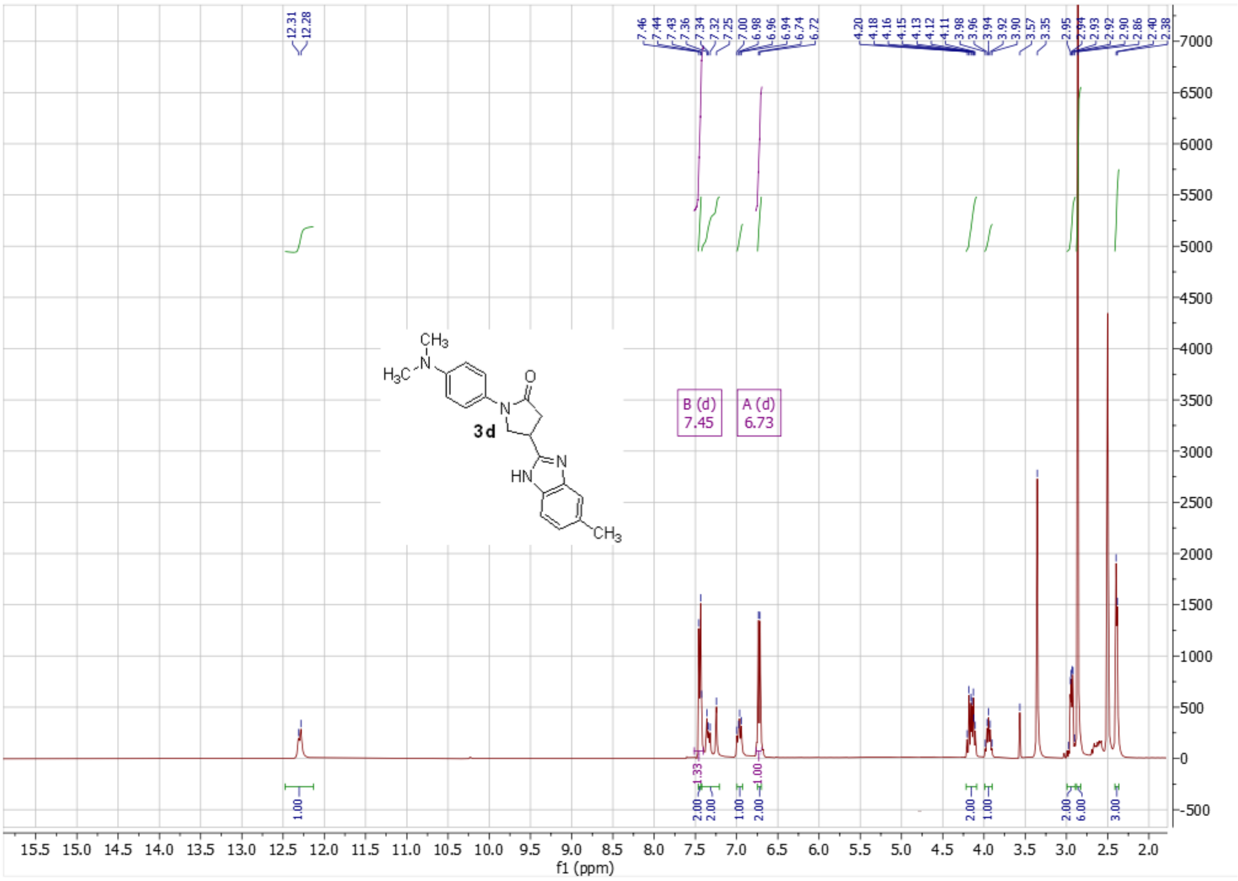

Figure S7. ^{1}H NMR spectrum of compound **3d.**

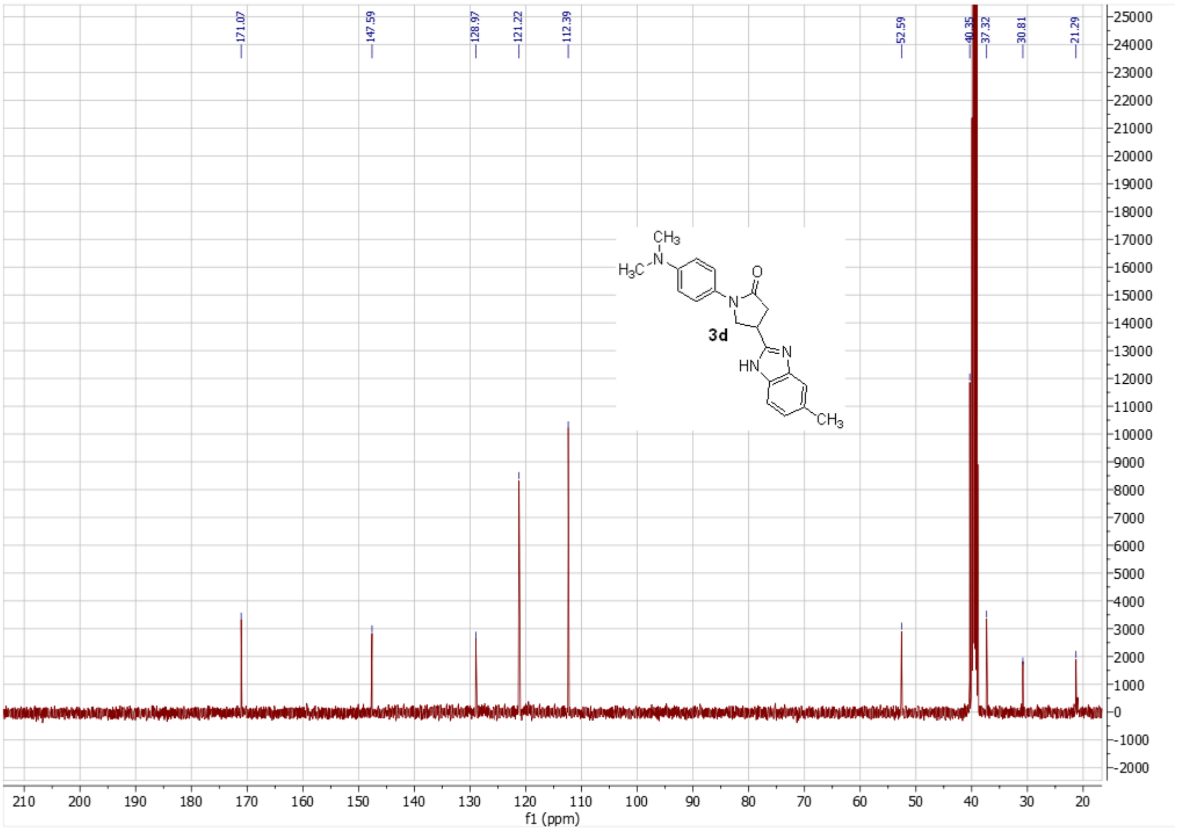

Figure S8. ^{13}C NMR spectrum of compound **3d.**

1-(4-(Dimethylamino)phenyl)-5-oxopyrrolidine-3-carbohydrazide (4)

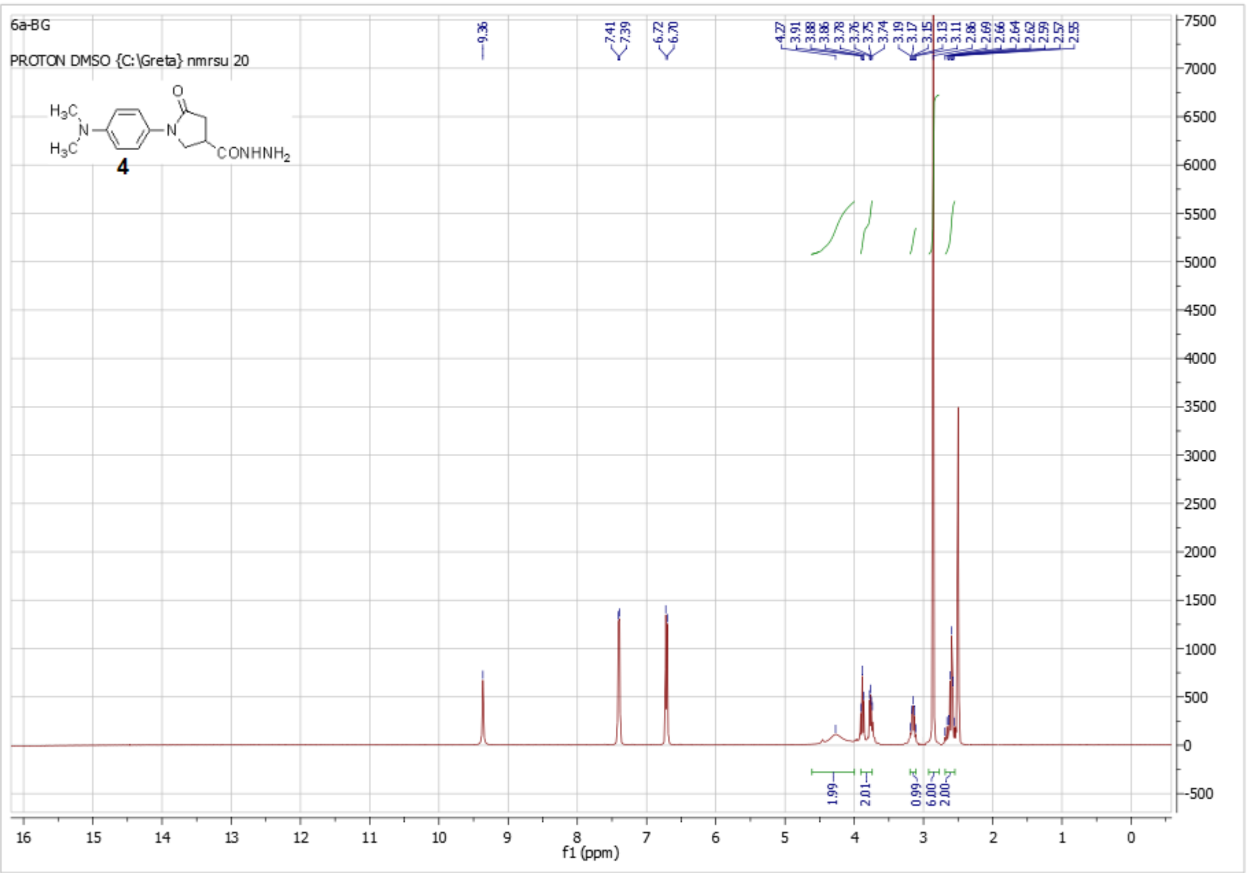

Figure S9. ^{1}H NMR spectrum of compound **4.**

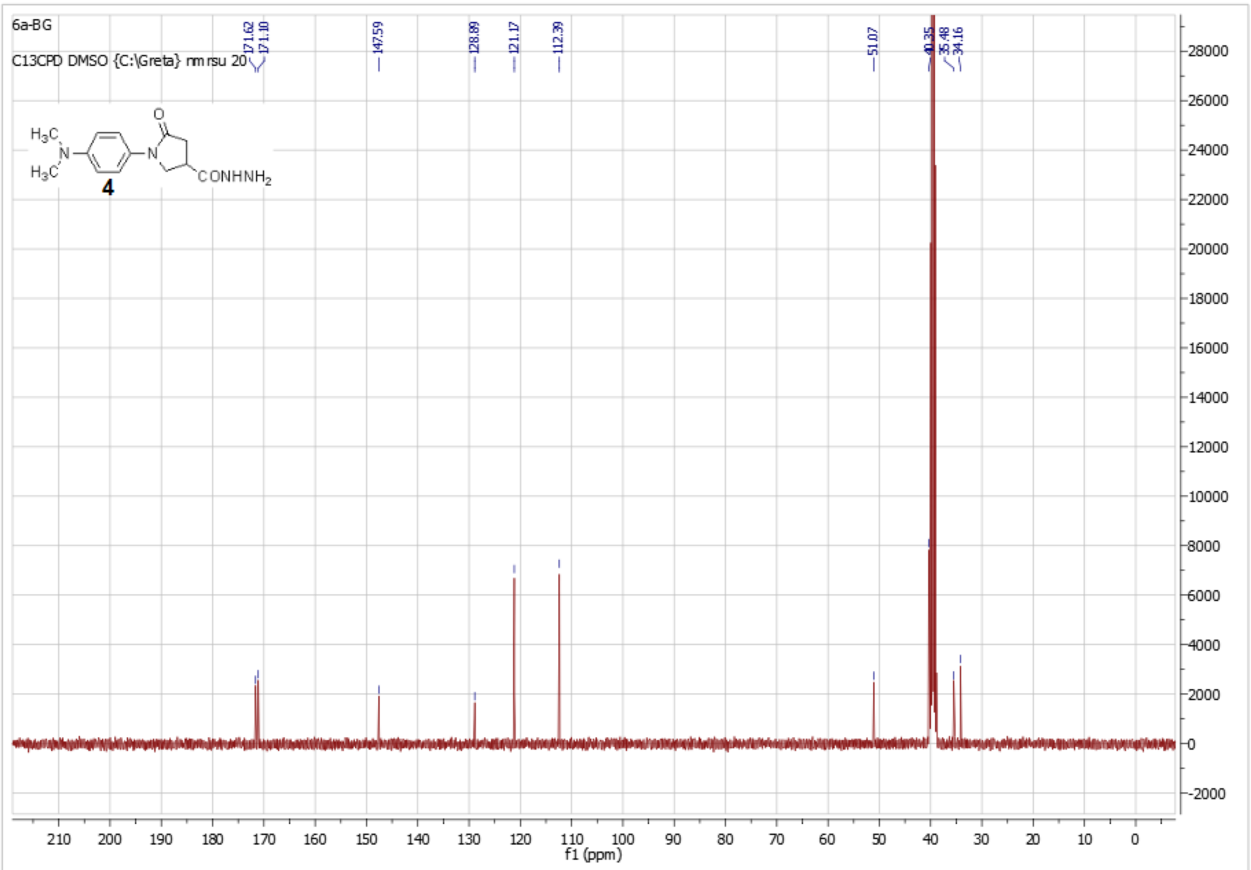

Figure S10. ^{13}C NMR spectrum of compound **4.**

***N′*-benzylidene-1-(4-(dimethylamino)phenyl)-5-oxopyrrolidine-3-carbohydrazide (5a)**

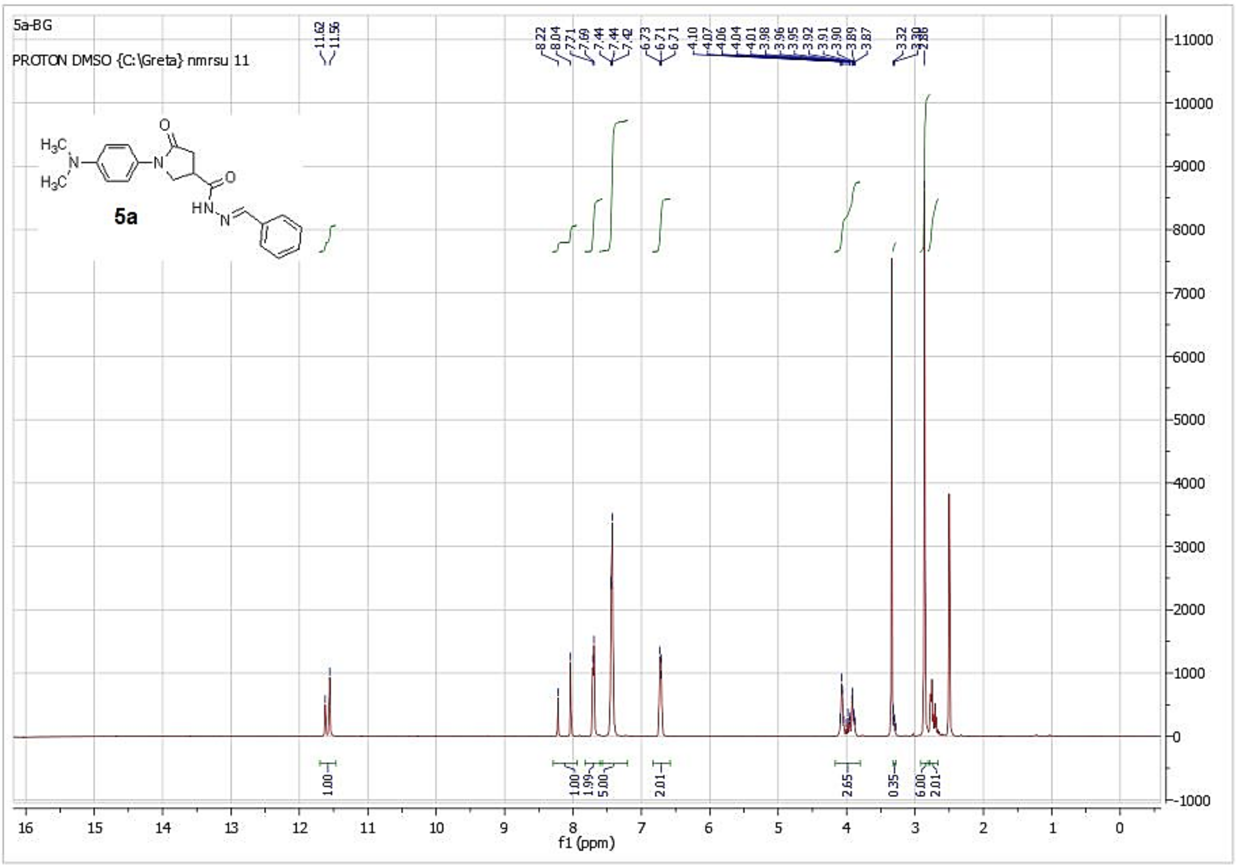

Figure S11. ^{1}H NMR spectrum of compound **5a.**

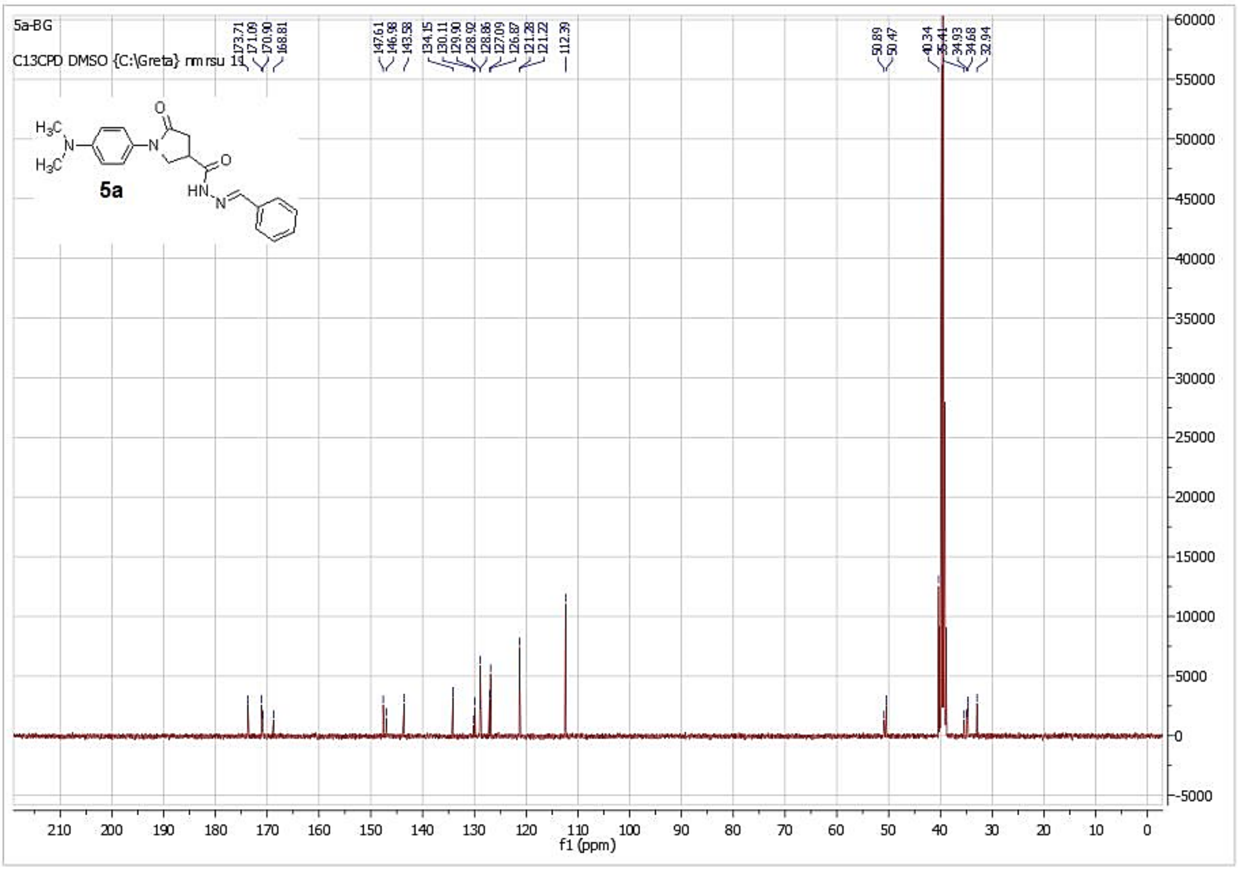

Figure S12. ^{13}C NMR spectrum of compound **5a.**

N'-(4-chlorobenzylidene)-1-(4-(dimethylamino)phenyl)-5-oxopyrrolidine-3-carbohydrazide (5b)

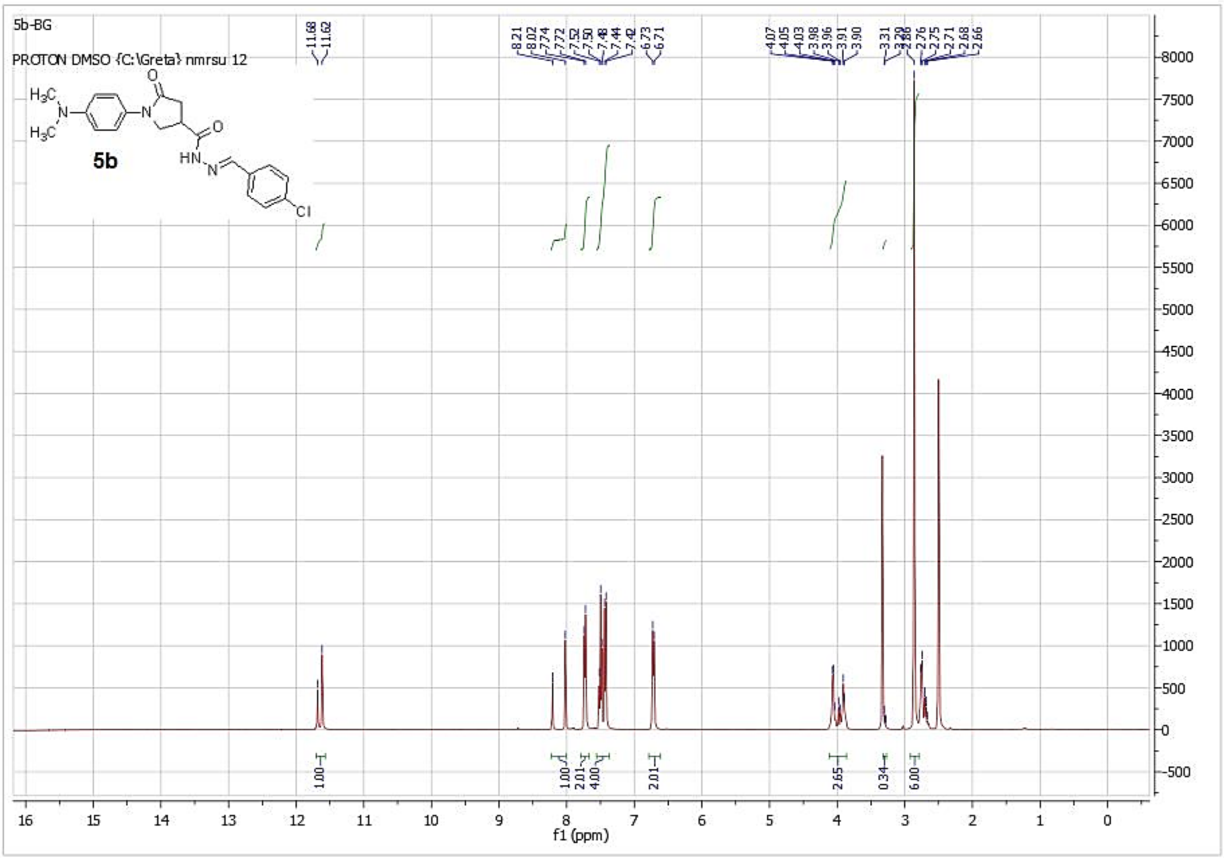

Figure S13. ^{1}H NMR spectrum of compound **5b.**

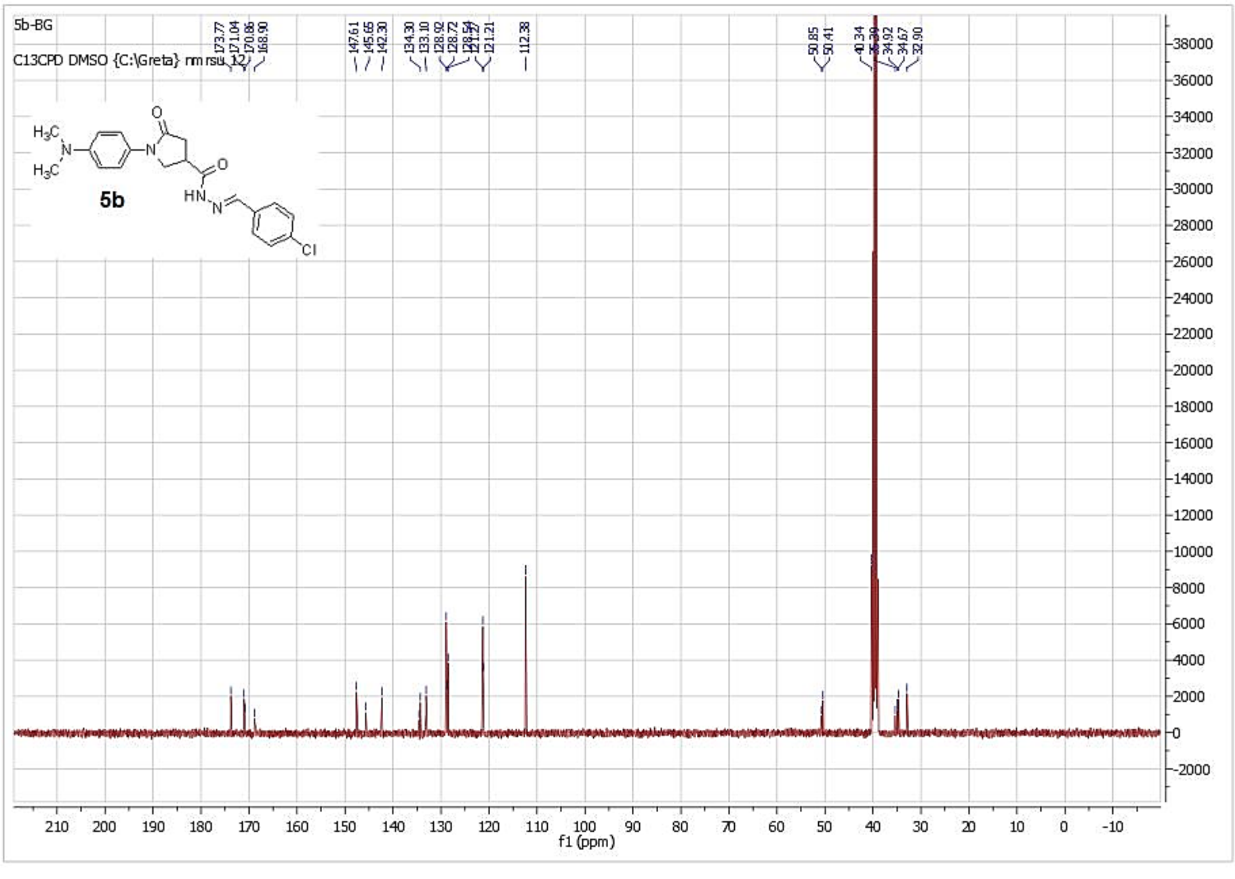

Figure S14. ^{13}C NMR spectrum of compound **5b.**

***N′*-(4-bromobenzylidene)-1-(4-(dimethylamino)phenyl)-5-oxopyrrolidine-3-carbohydrazide (5c)**

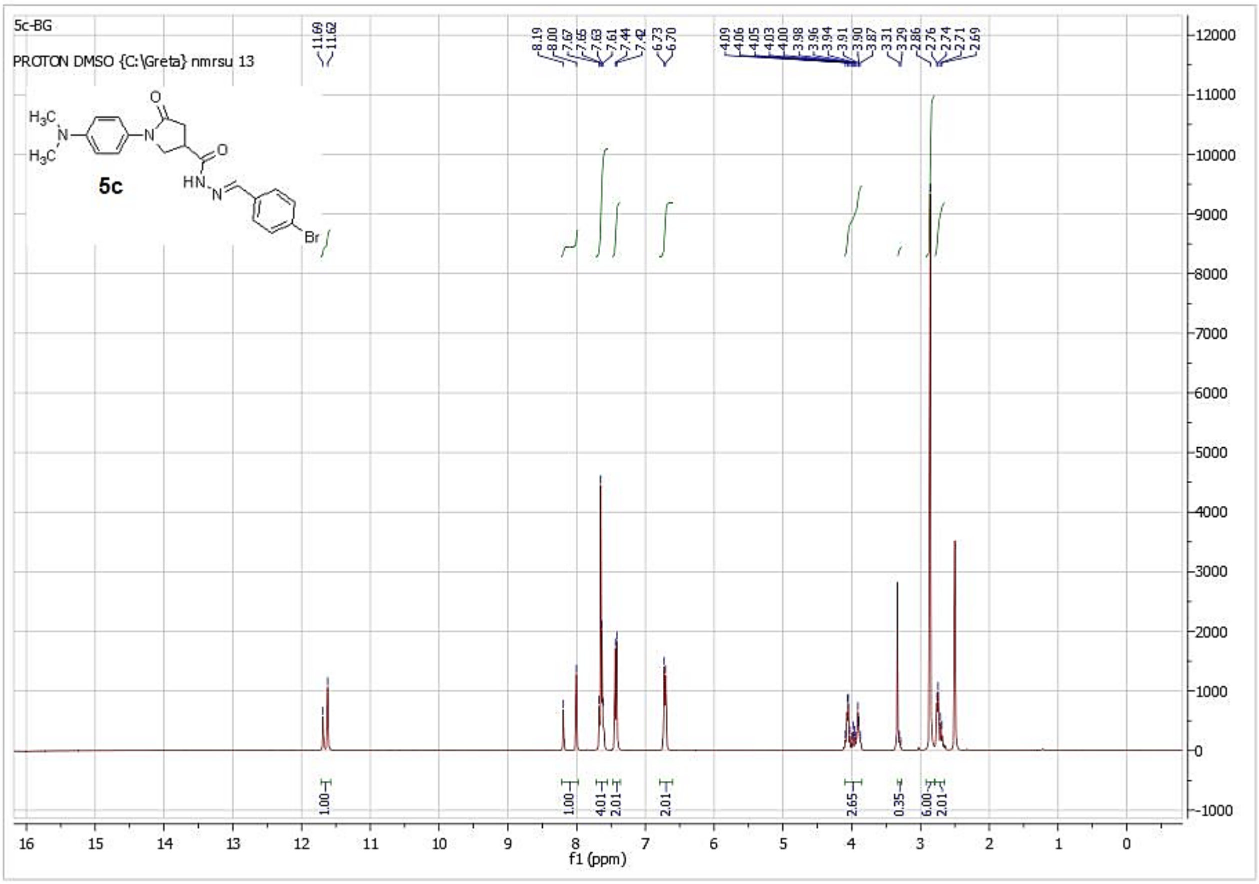

Figure S15. ^{1}H NMR spectrum of compound **5c.**

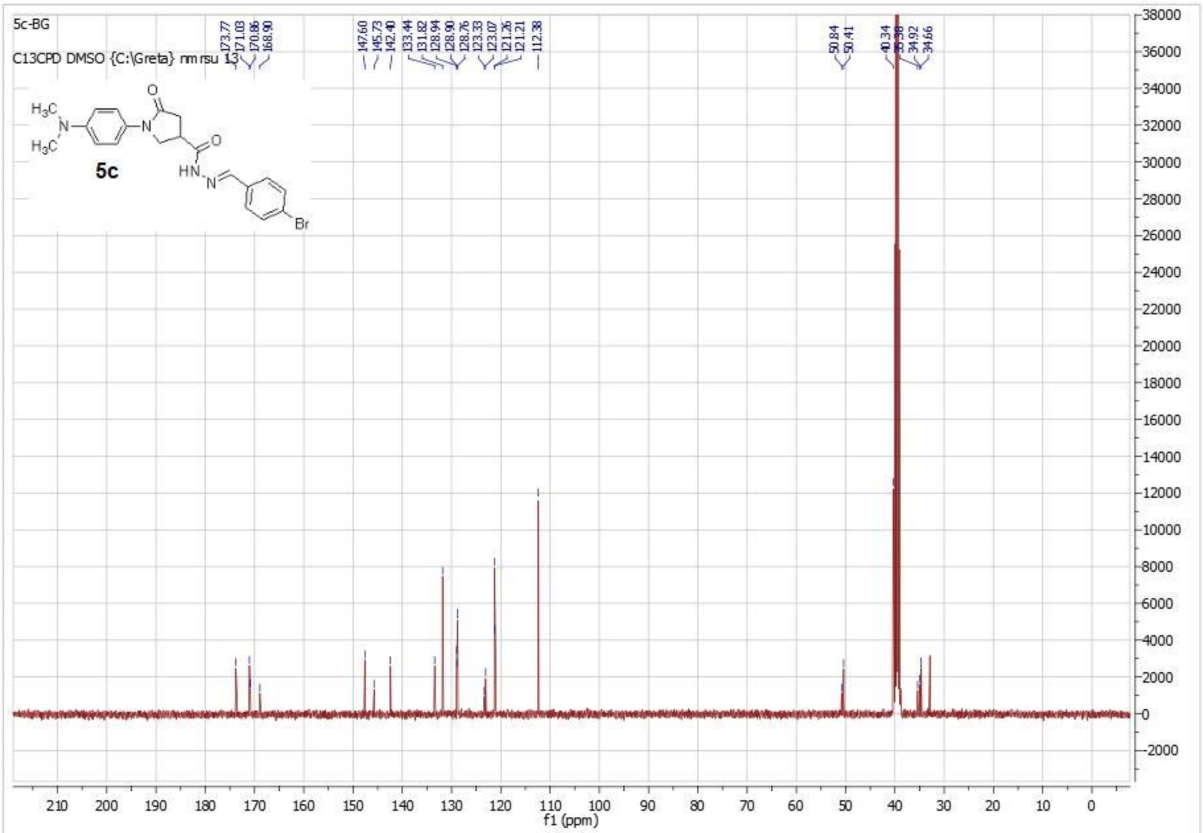

Figure S16. ^{13}C NMR spectrum of compound **5c.**

***N′*-((4-dimethylamino)benzylidene)-1-(4-(dimethylamino)phenyl)-5-oxopyrrolidine-3-carbohydrazide (5d)**

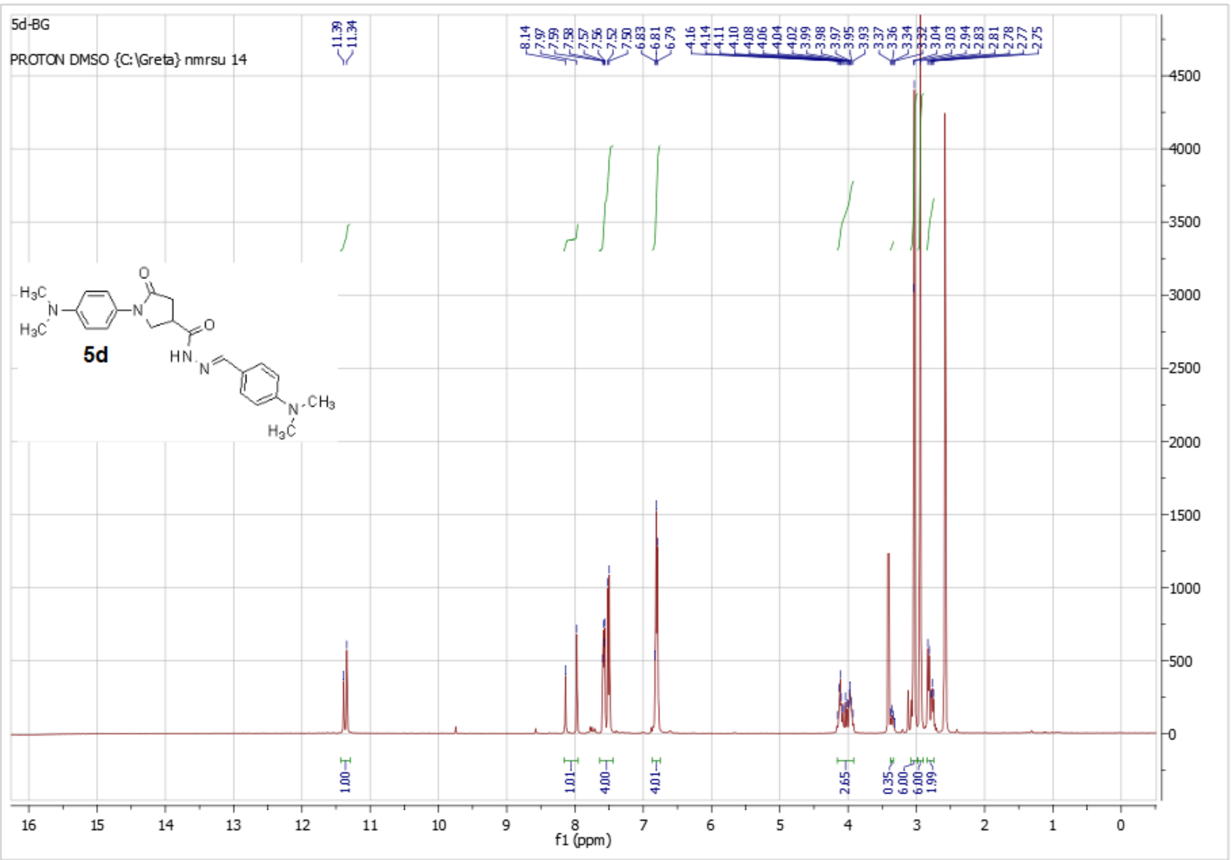

Figure S17. ^{1}H NMR spectrum of compound **5d.**

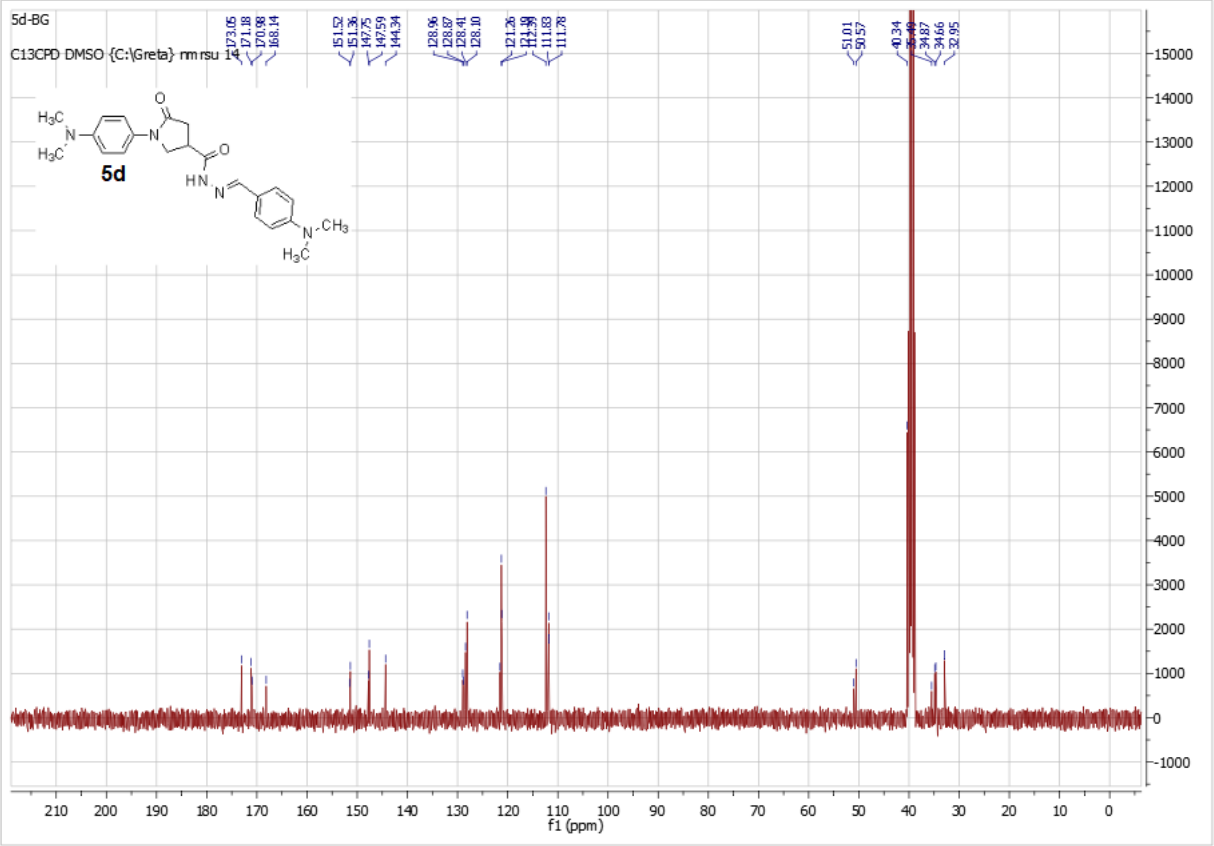

Figure S18. ^{13}C NMR spectrum of compound **5d.**

***N′*-(4-methoxybenzylidene)-1-(4-(dimethylamino)phenyl)-5-oxopyrrolidine-3-carbohydrazide (5e)**

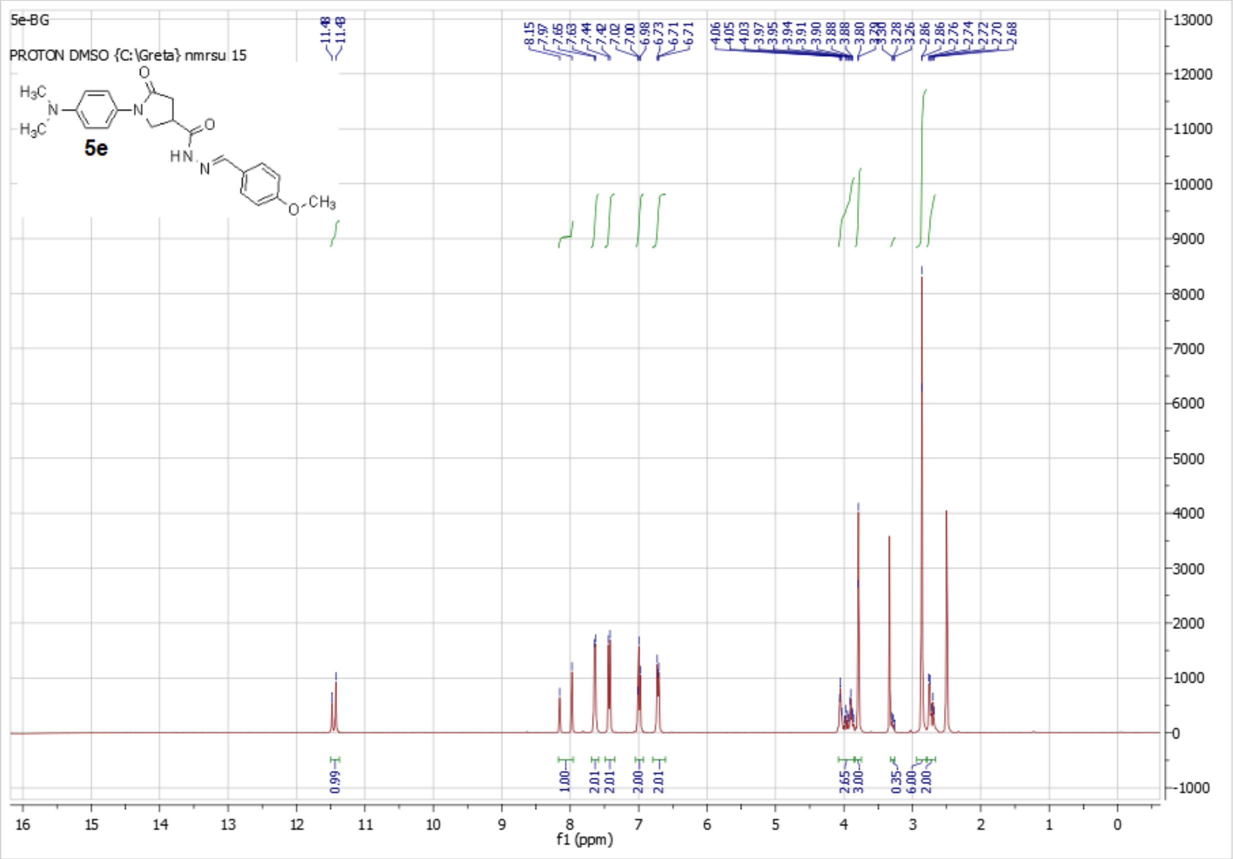

Figure S19. ^{1}H NMR spectrum of compound **5e.**

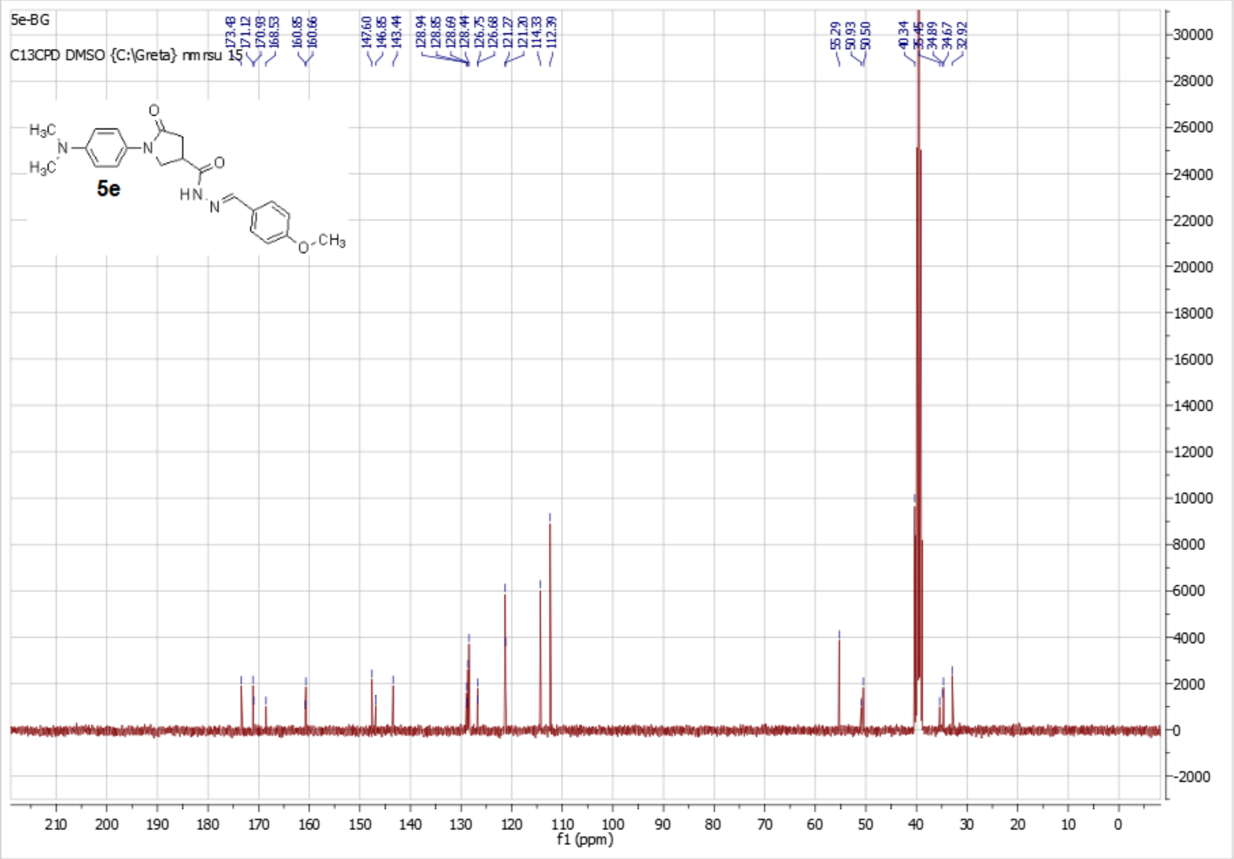

Figure S20. ^{13}C NMR spectrum of compound **5e.**

N′-(2,5-dimethoxybenzylidene)-1-(4-(dimethylamino)phenyl)-5-oxopyrrolidine-3-carbohydrazide (5f)

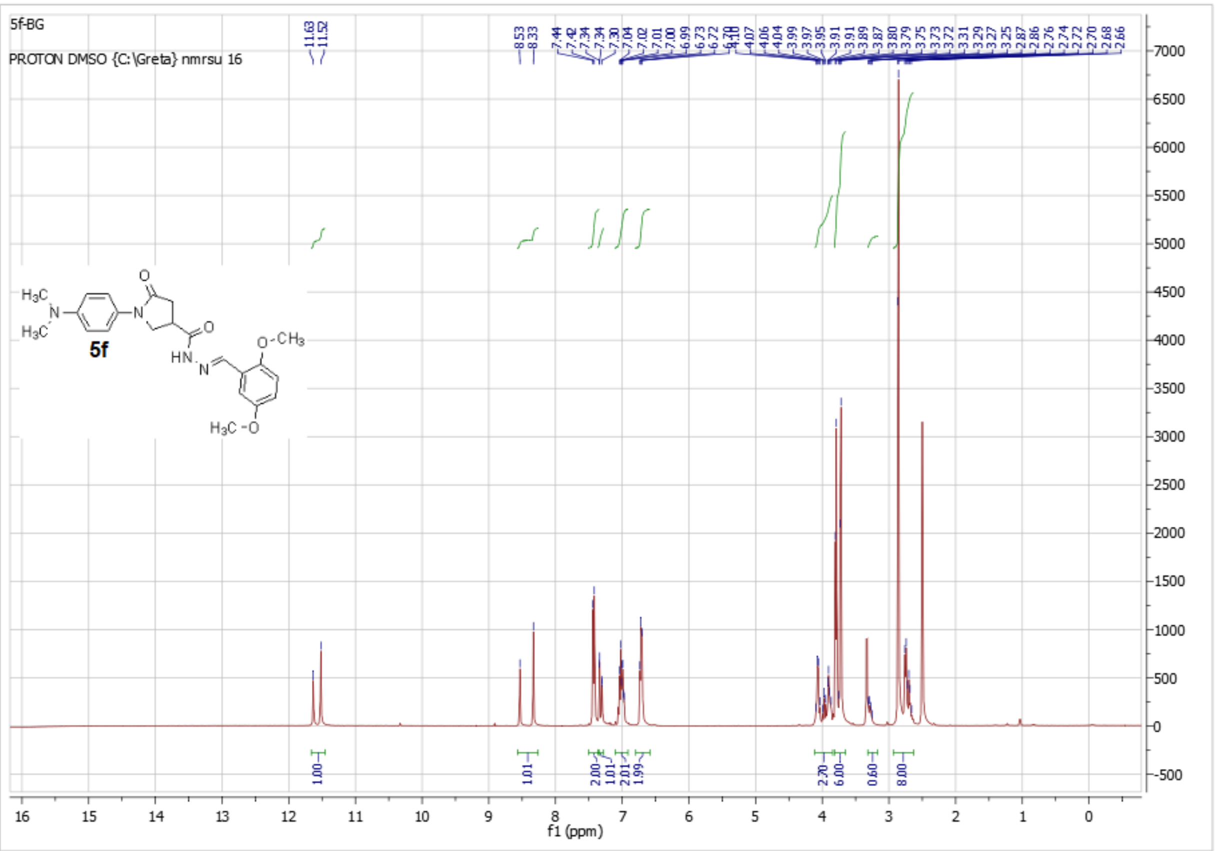

Figure S21. ^{1}H NMR spectrum of compound **5f.**

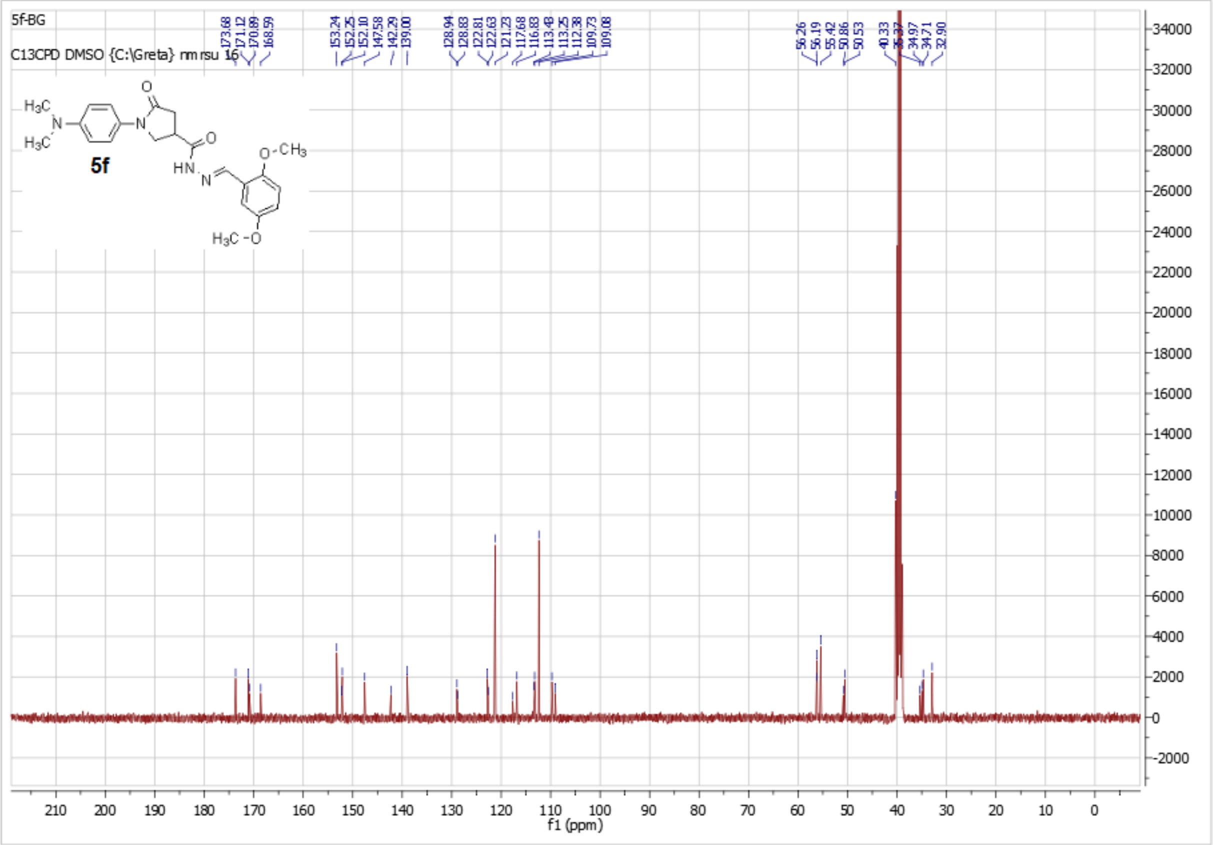

Figure S22. ^{13}C NMR spectrum of compound **5f.**

***N′*-(2,4,6-trimethoxybenzylidene)-1-(4-(dimethylamino)phenyl)-5-oxopyrrolidine-3-carbohydrazide (5g)**

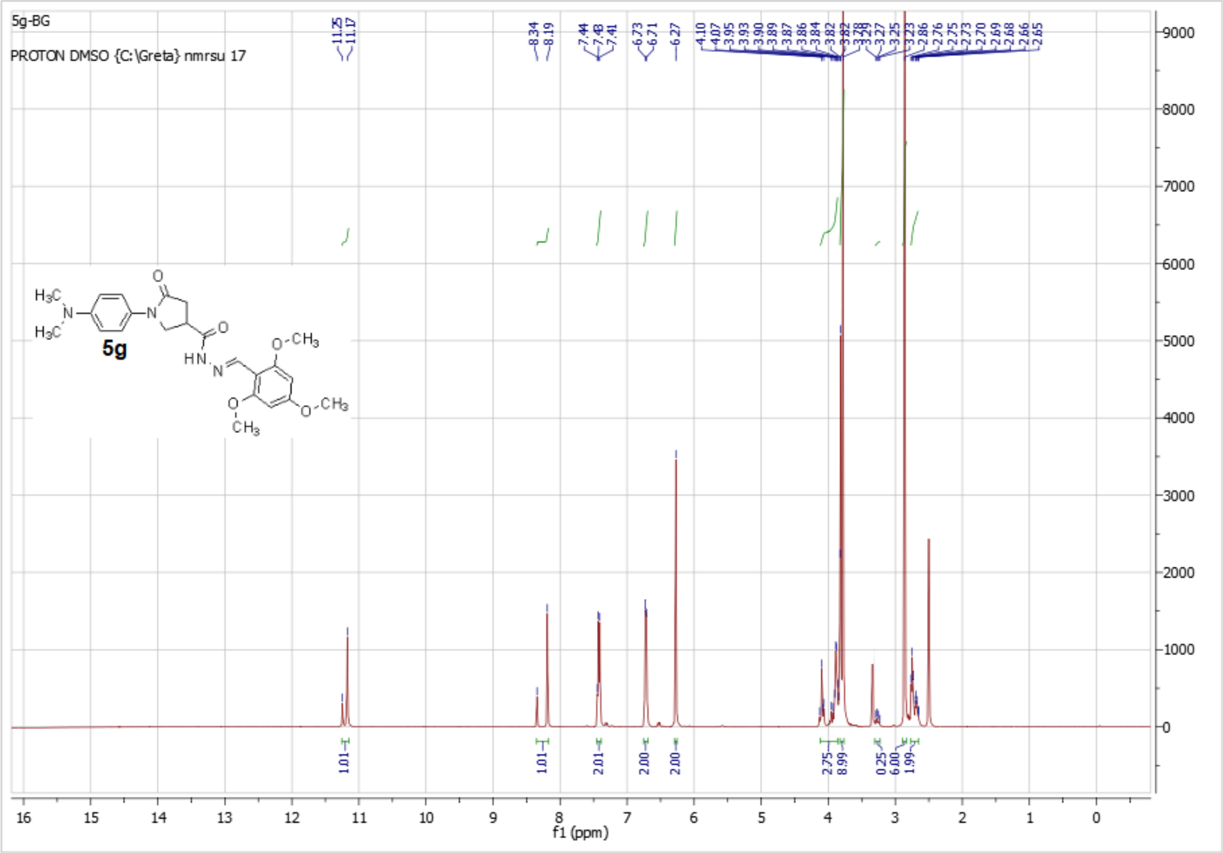

Figure S23. ^{1}H NMR spectrum of compound **5g.**

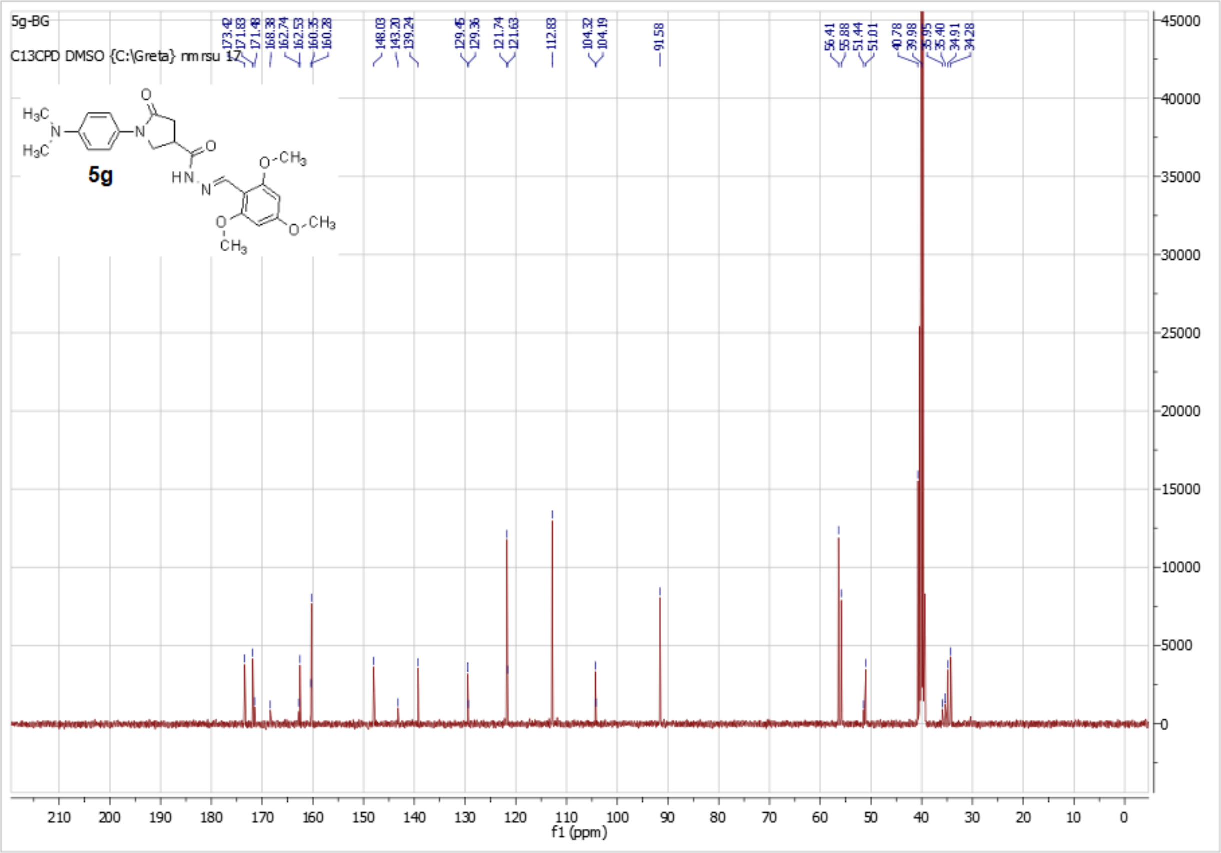

Figure S24. ^{13}C NMR spectrum of compound **5g.**

N′-(3,4,5-trimethoxybenzylidene)-1-(4-(dimethylamino)phenyl)-5-oxopyrrolidine-3-carbohydrazide (5h)

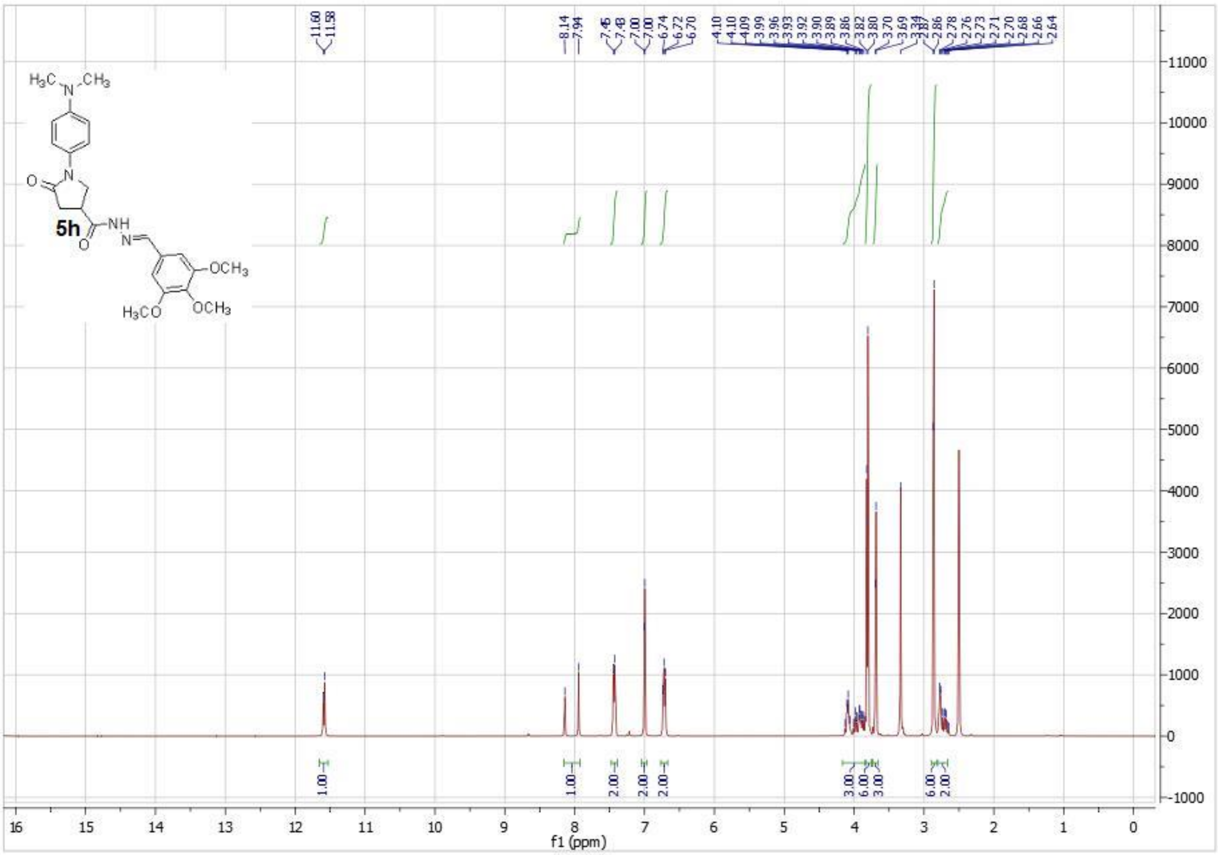

Figure S25. ^{1}H NMR spectrum of compound **5h.**

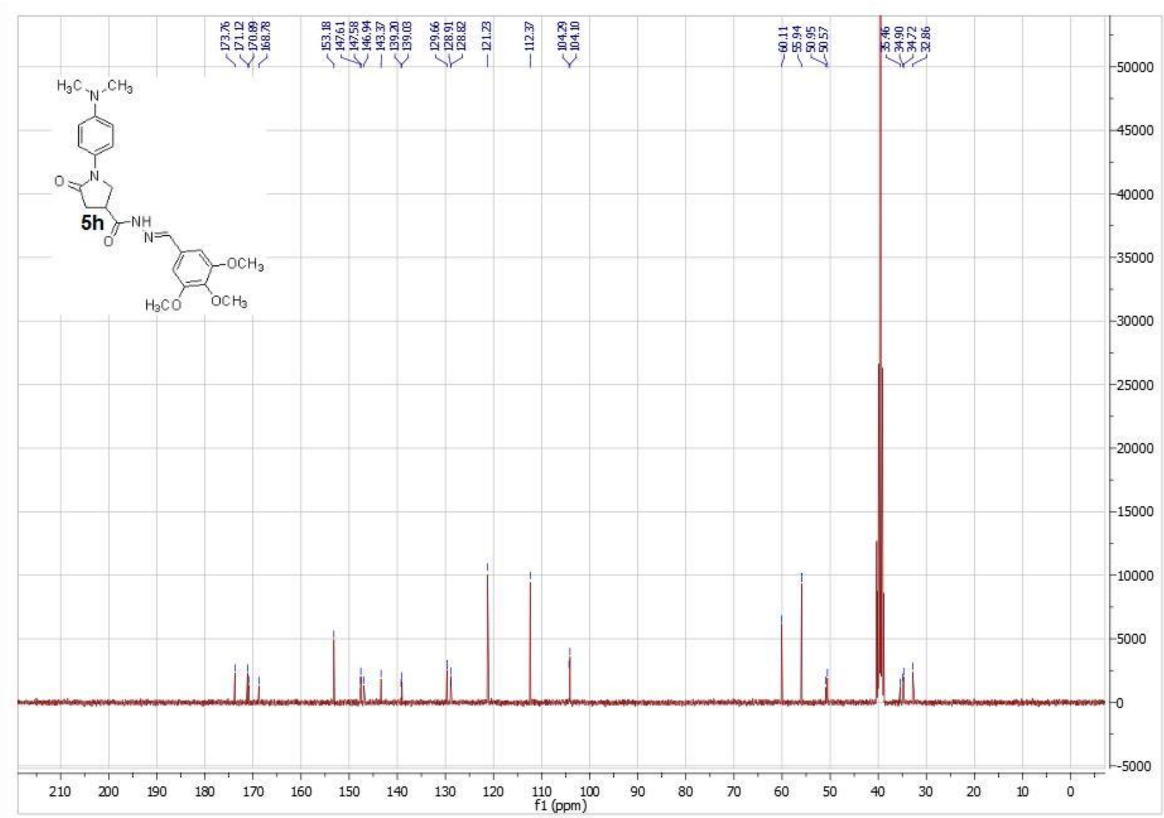

Figure S26. ^{13}C NMR spectrum of compound **5h.**

1-(4-(Dimethylamino)phenyl)-*N'*-(4-nitrobenzylidene)-5-oxopyrrolidine-3-carbohydrazide (5i)

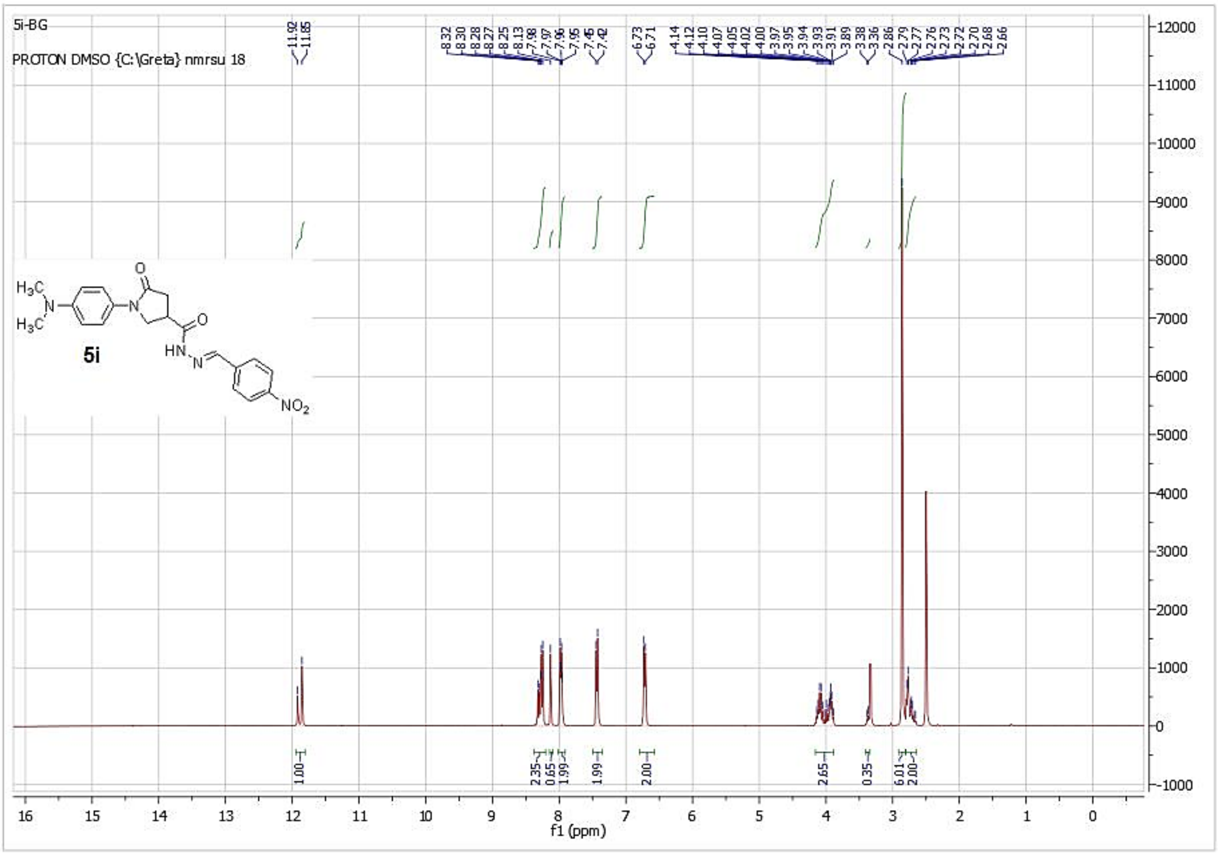

Figure S27. ^{1}H NMR spectrum of compound **5i.**

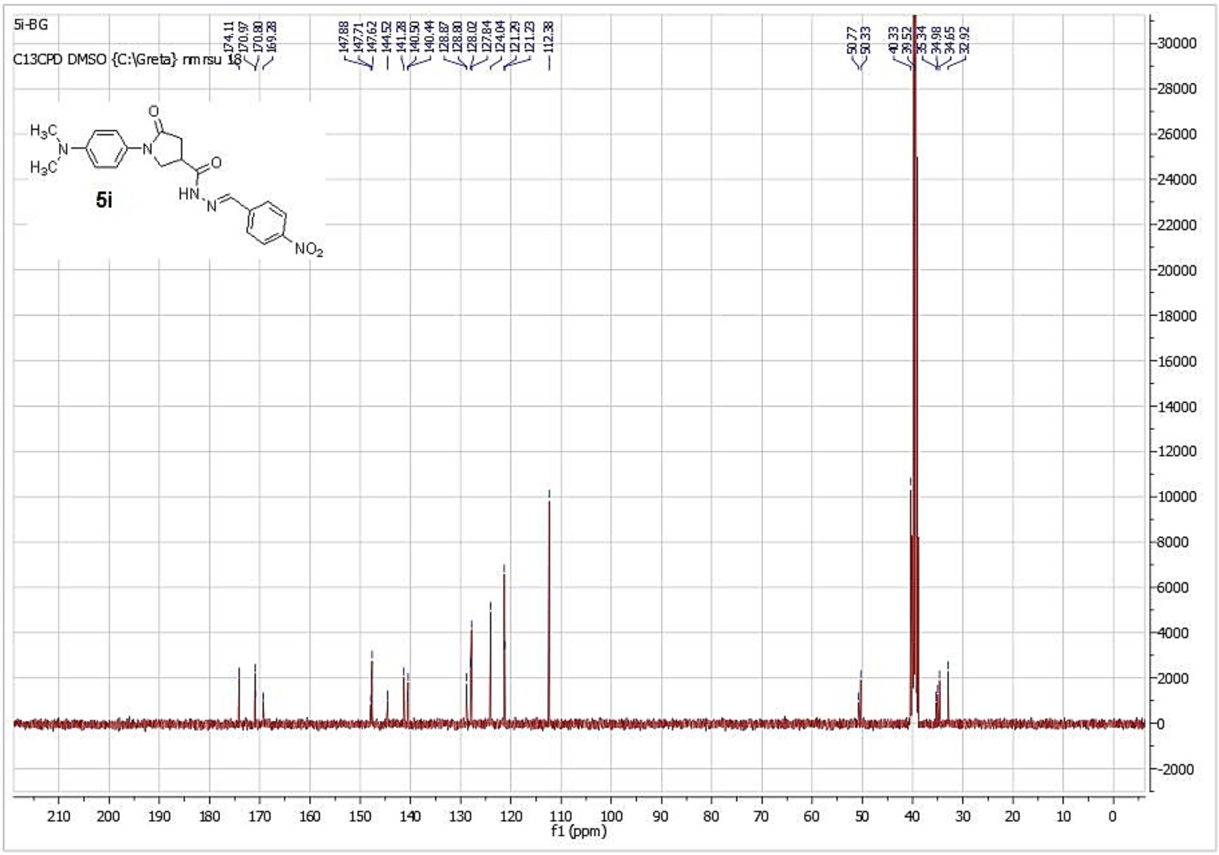

Figure S28. ^{13}C NMR spectrum of compound **5i.**

1-(4-(Dimethylamino)phenyl)-5-oxo-N'-(thiophen-2-ylmethylene)pyrrolidine-3-carbohydrazide (5j)

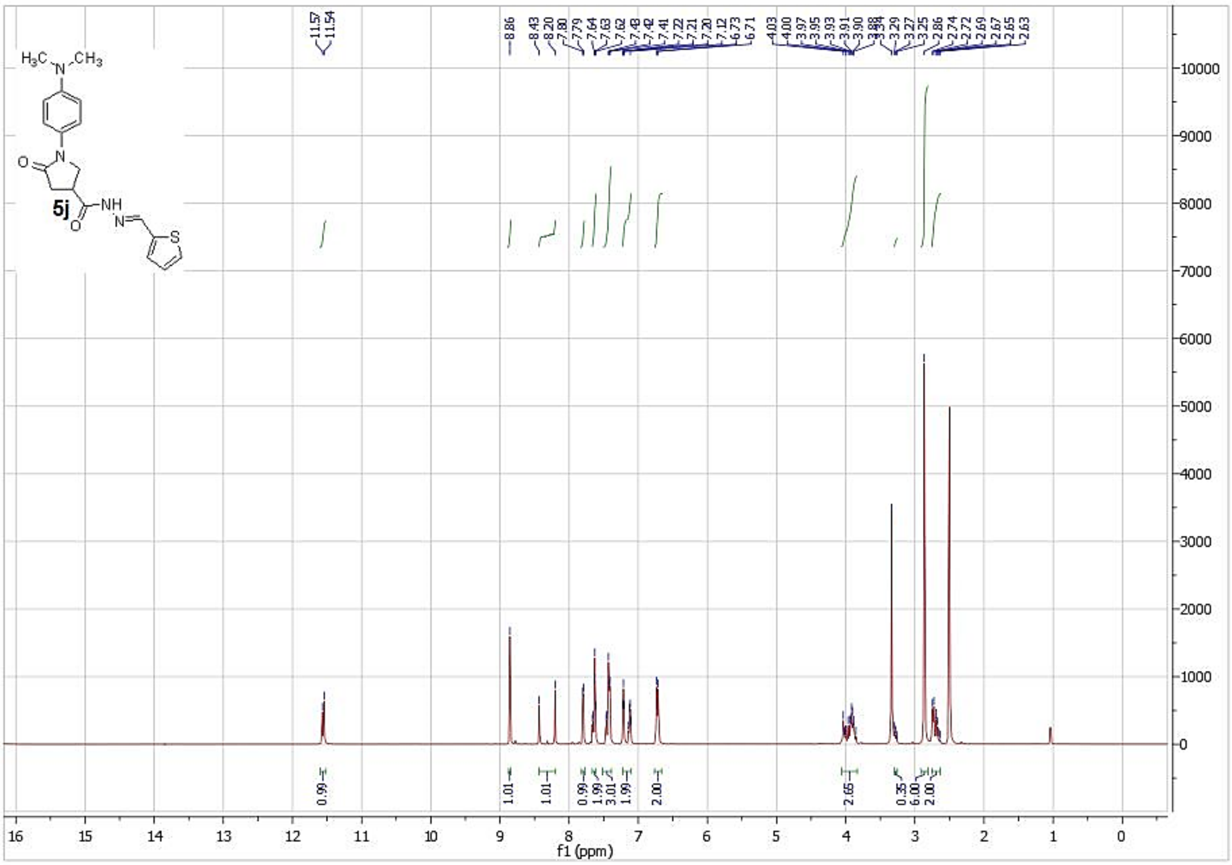

Figure S29. ^{1}H NMR spectrum of compound **5j.**

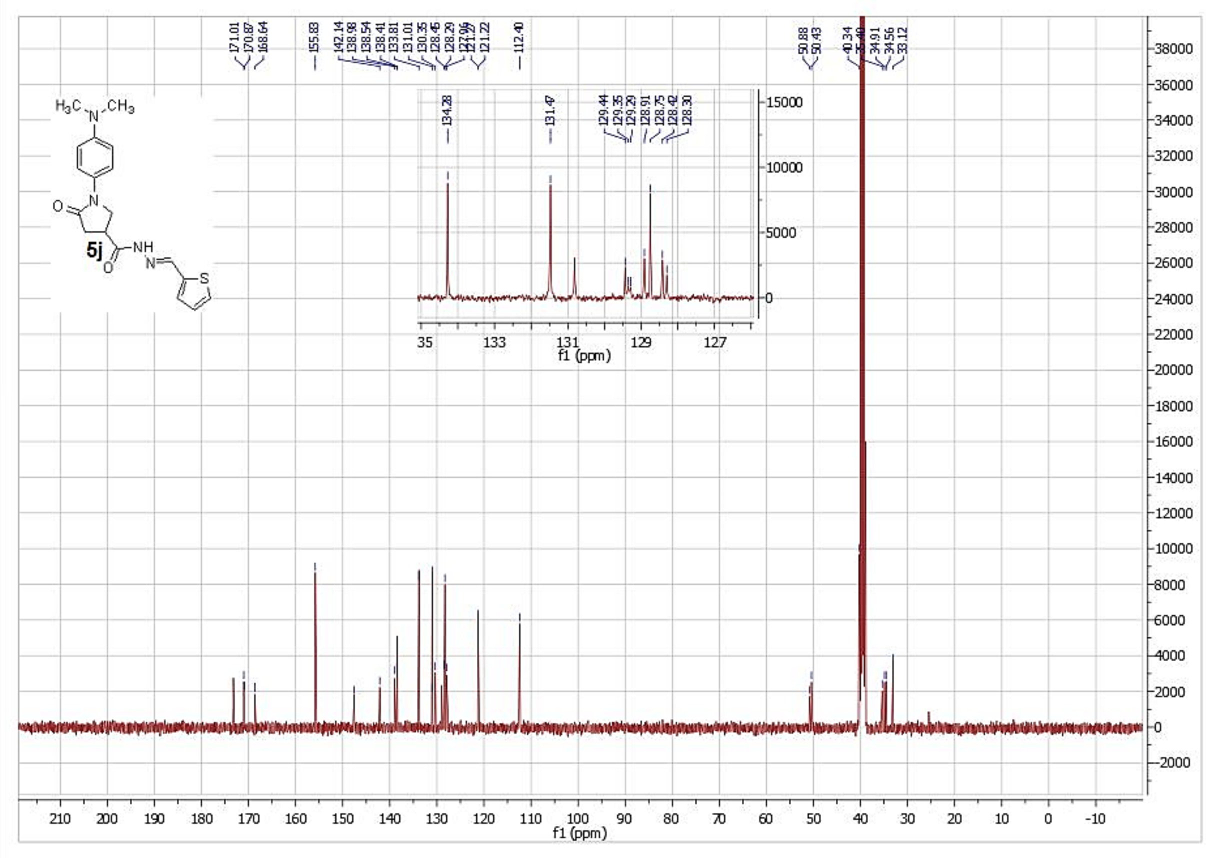

Figure S30. ^{13}C NMR spectrum of compound **5j.**

1-(4-(Dimethylamino)phenyl)-*N′*-((5-nitrothiophen-2-yl)methylene)-5-oxopyrrolidine-3-carbohydrazide (5k)

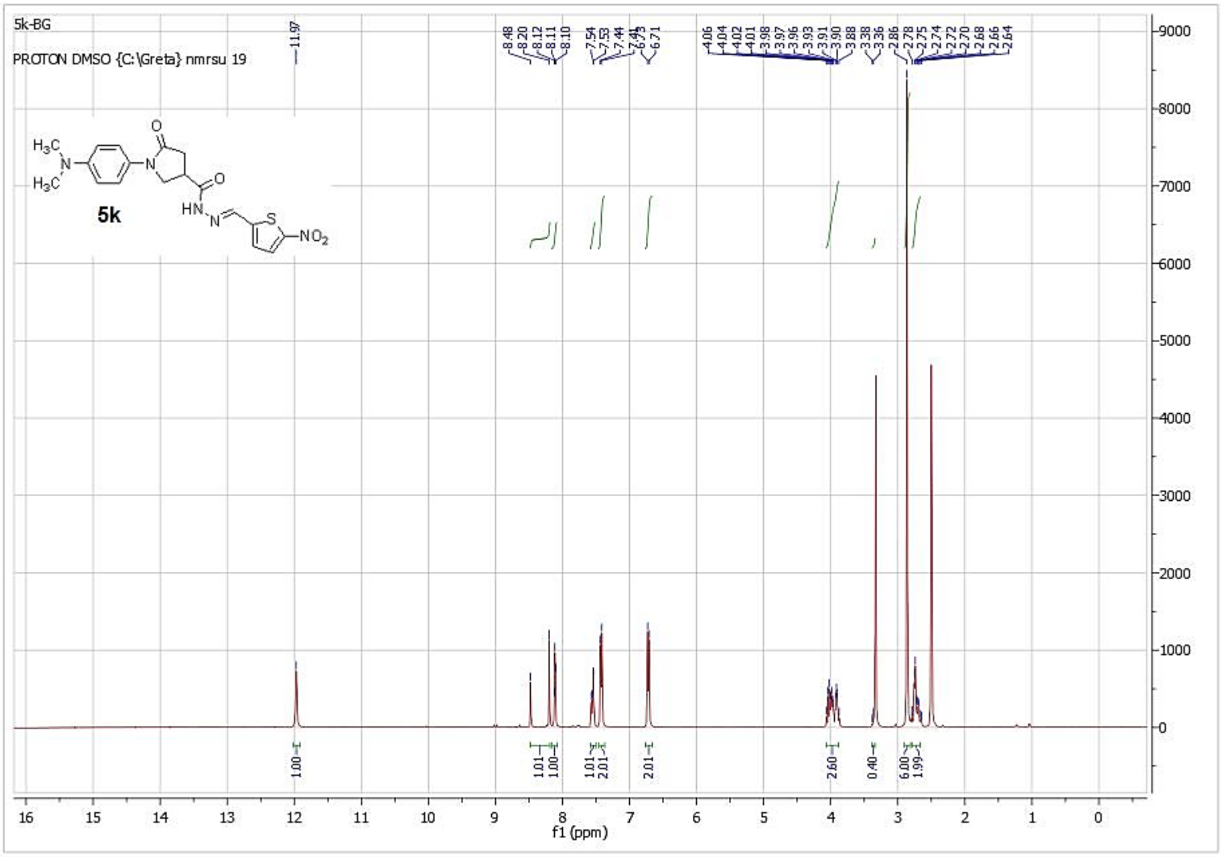

Figure S31. ^{1}H NMR spectrum of compound **5k.**

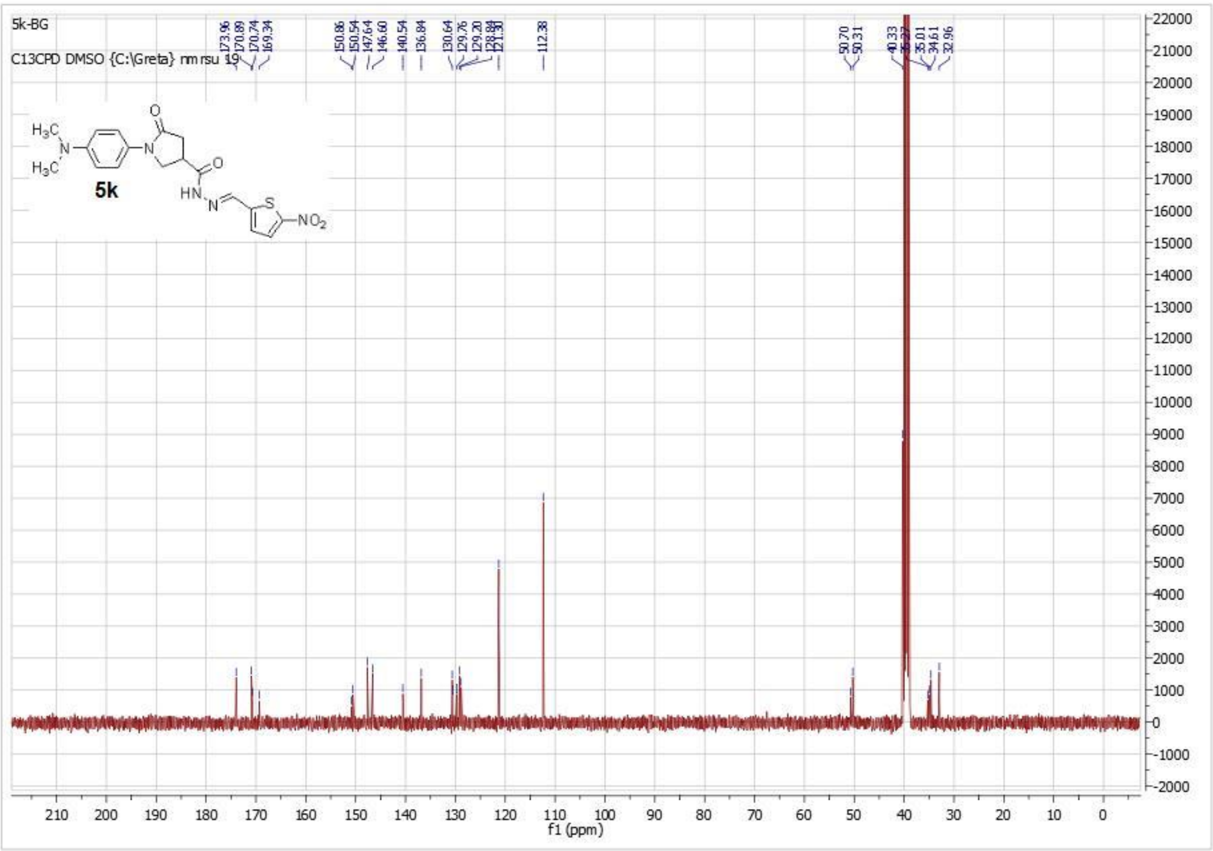

Figure S32. ^{13}C NMR spectrum of compound **5l**.

1-(4-(Dimethylamino)phenyl)-N'-(naphthalen-1-ylmethylene)-5-oxopyrrolidine-3-carbohydrazide (5l)

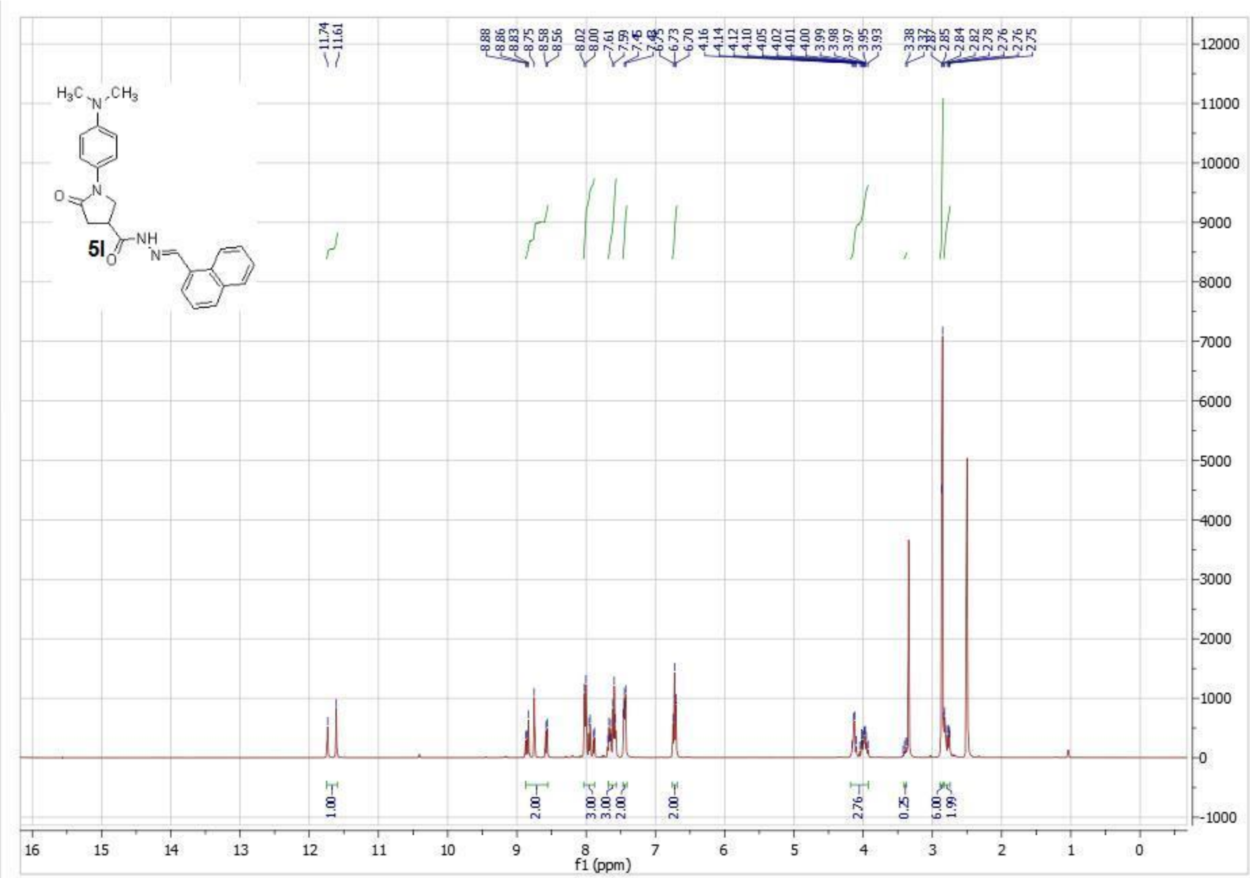

Figure S33. ^{1}H NMR spectrum of compound **5l.**

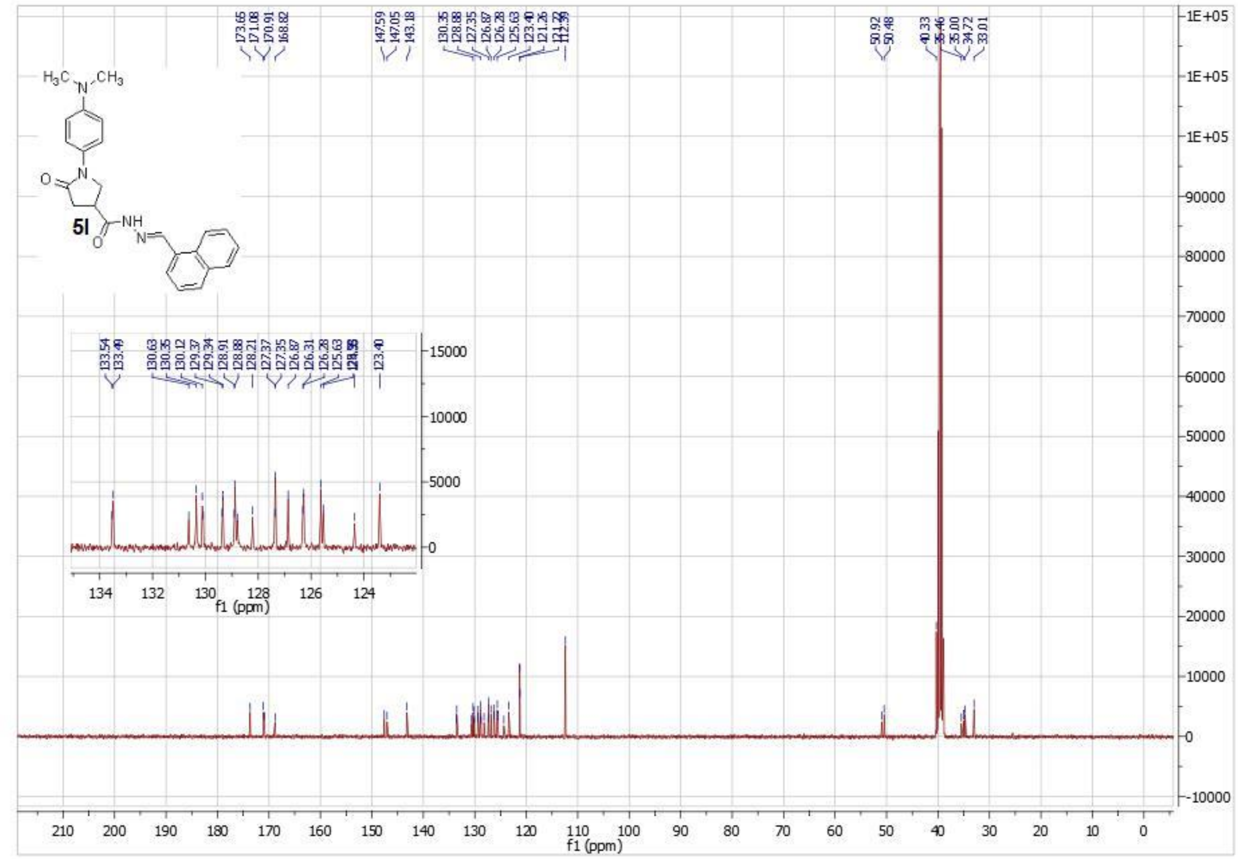

Figure S34. ^{13}C NMR spectrum of compound **5l**.

1-(4-(Dimethylamino)phenyl)-5-oxo-*N′*-(propan-2-ylidene)pyrrolidine-3-carbohydrazide (6a)

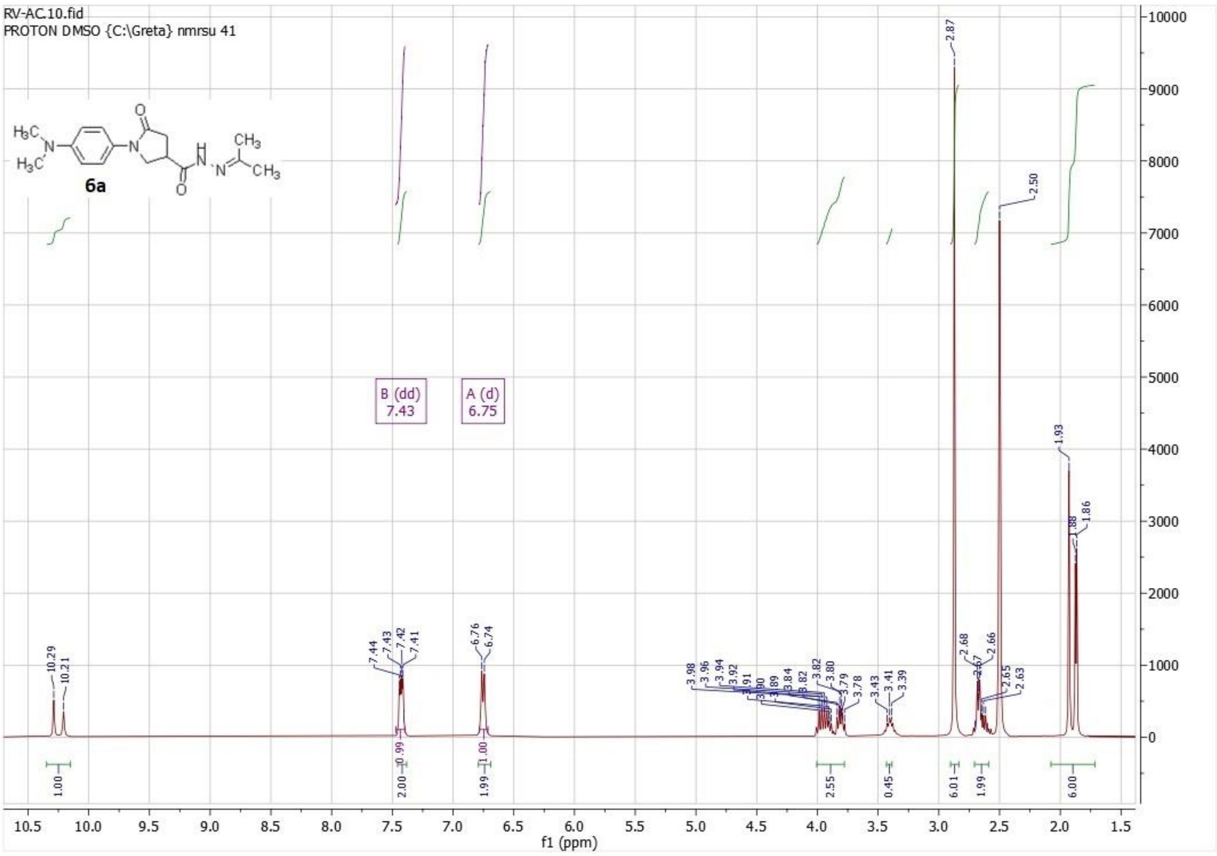

Figure S35. ^{1}H NMR spectrum of compound **6a.**

***N′*-(butan-2-ylidene)-1-(4-(dimethylamino)phenyl)-5-oxopyrrolidine-3-carbohydrazide (6b)**

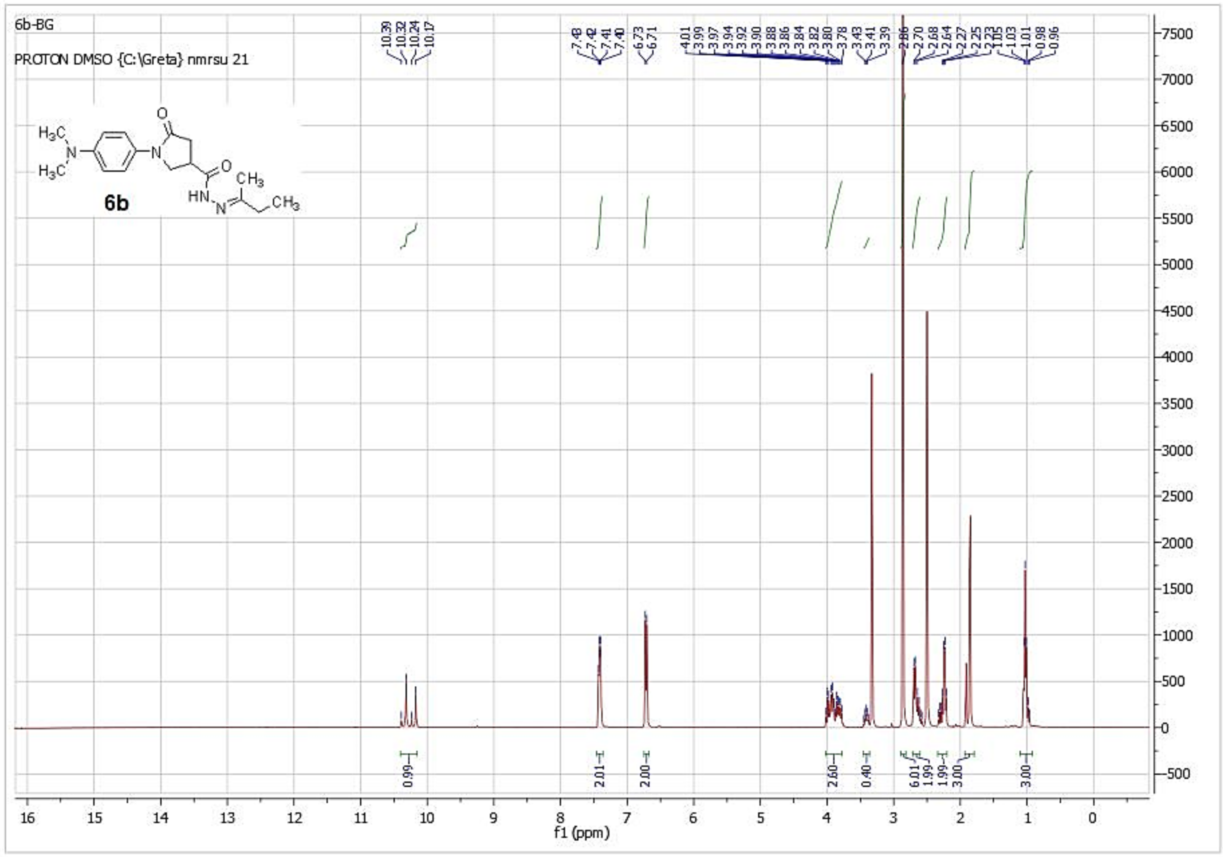

Figure S36. ^{1}H NMR spectrum of compound **6b.**

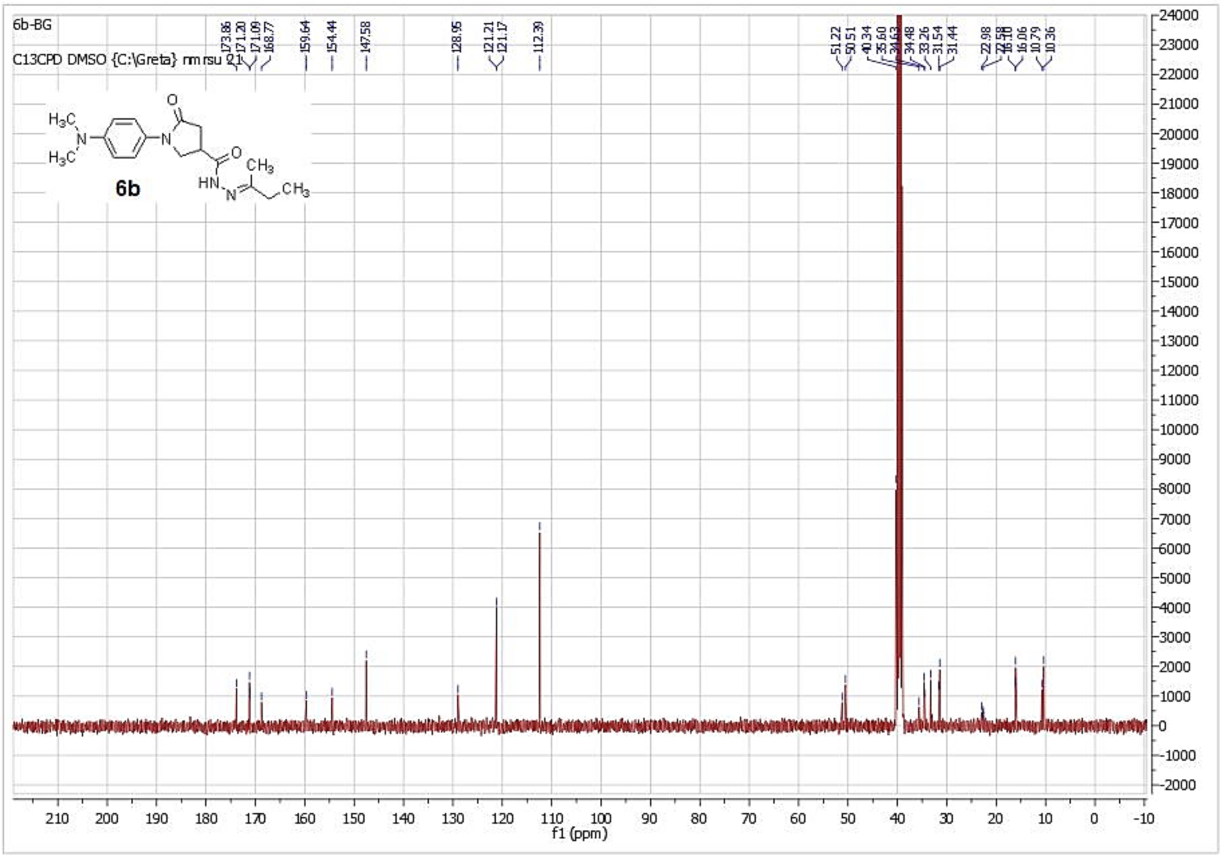

Figure S37. ^{13}C NMR spectrum of compound **6b.**

***N′*-(1-(4-aminophenyl)ethylidene)-1-(4-(dimethylamino)phenyl)-5-oxopyrrolidine-3-carbohydrazide (6c)**

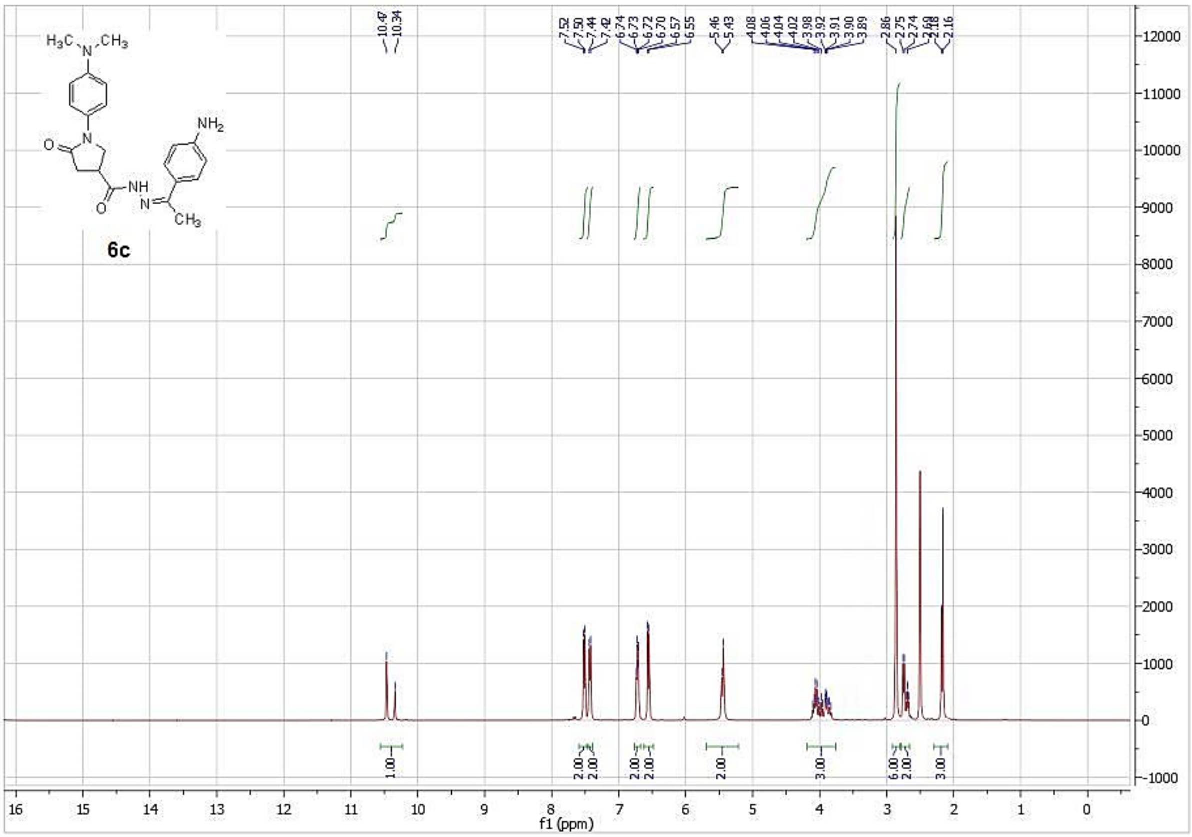

Figure S38. ^{1}H NMR spectrum of compound **6c.**

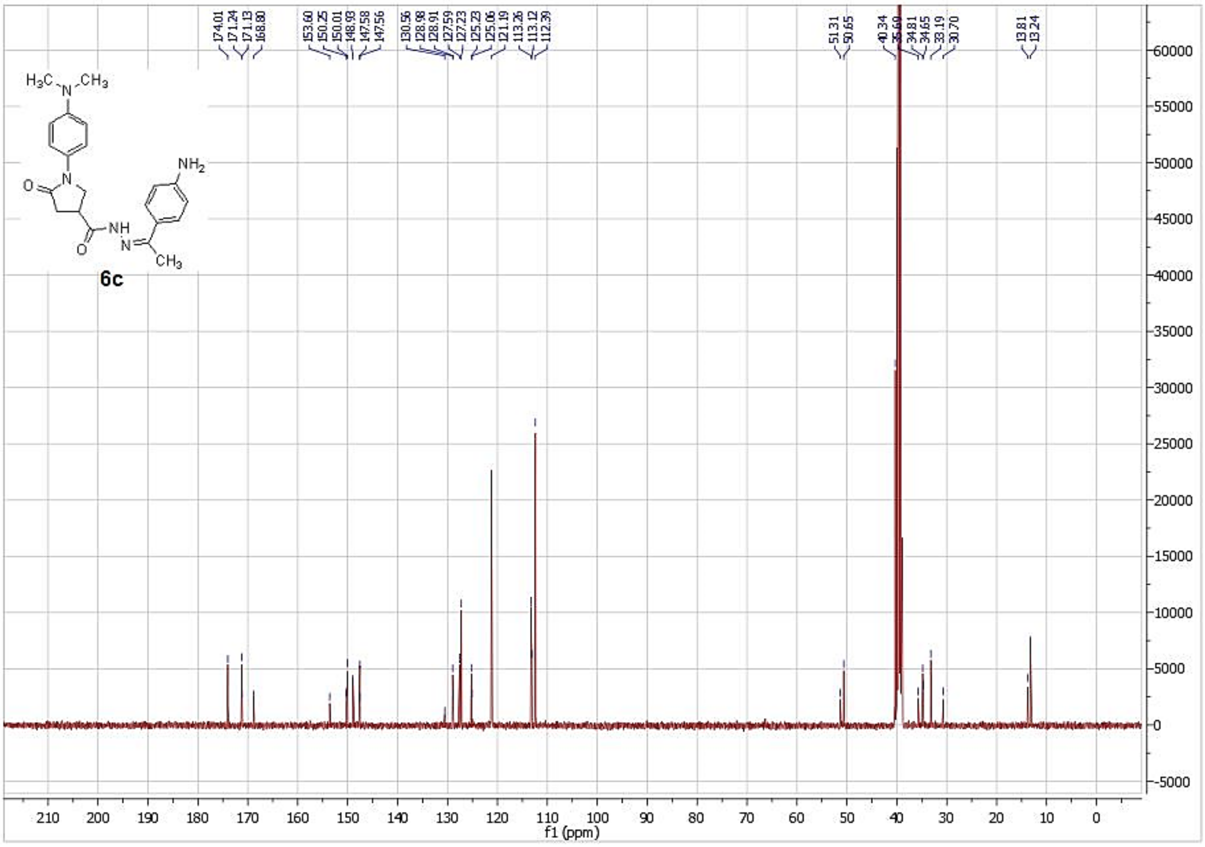

Figure S39. ^{13}C NMR spectrum of compound **6c.**

***N*-(2,5-dimethyl-*1H*-pyrrol-1-yl)-1-(4-(dimethylamino)phenyl)-5-oxopyrrolidine-3-carboxamide (7)**

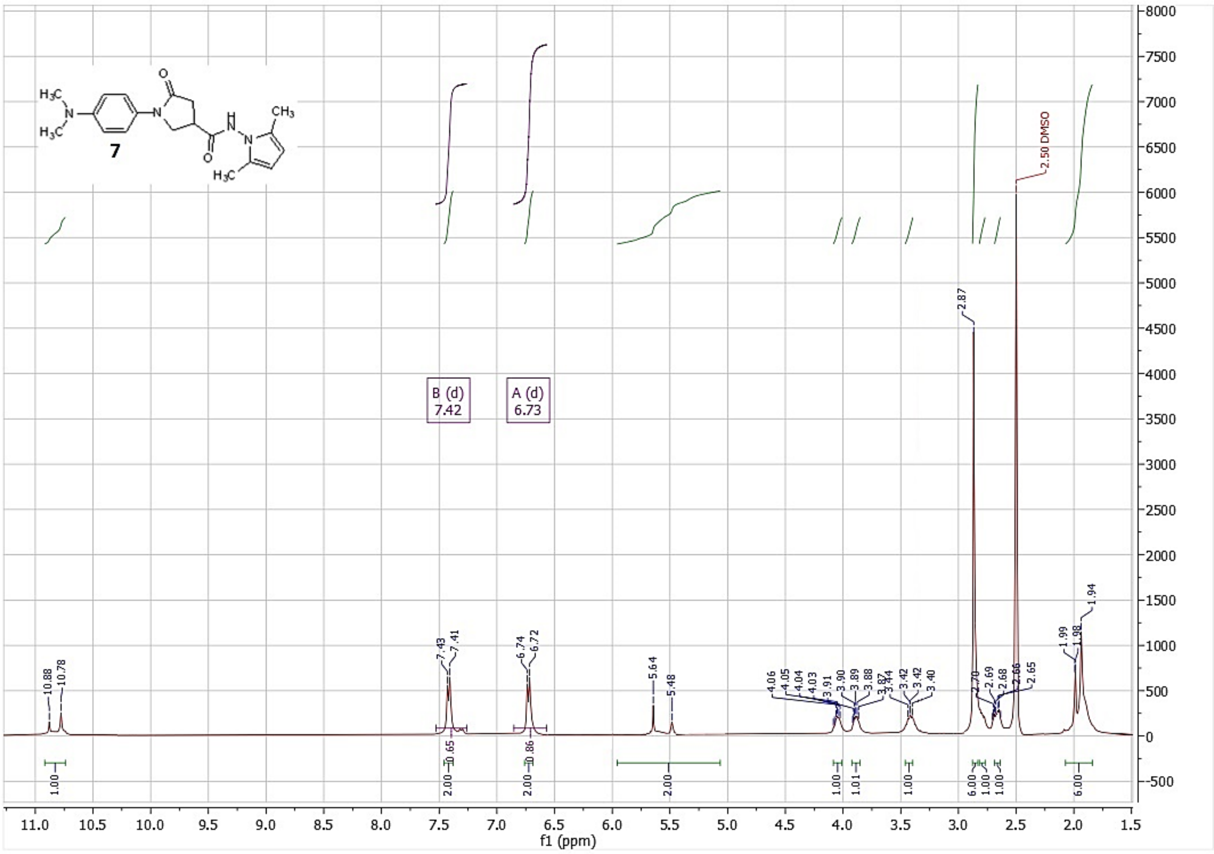

Figure S40. ^{1}H NMR spectrum of compound **7.**

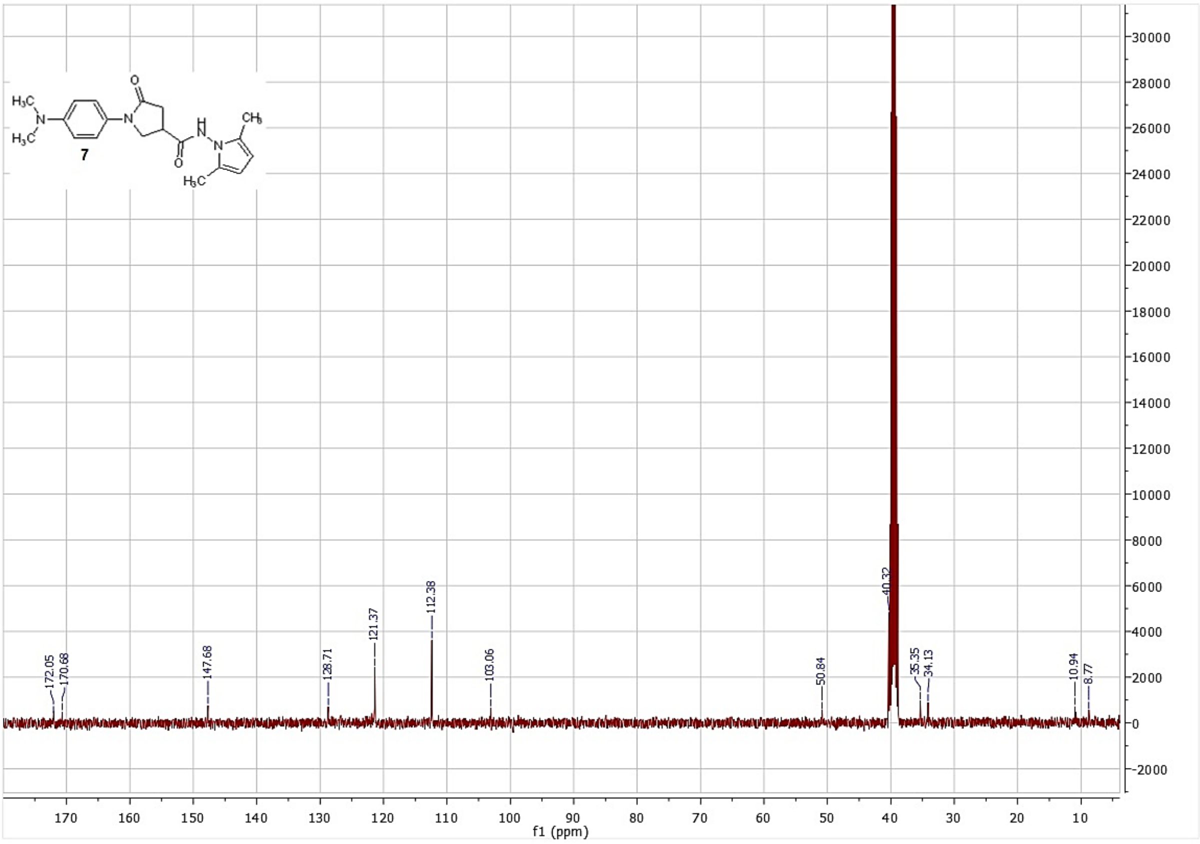

Figure S41. ^{13}C NMR spectrum of compound **7.**

1-(1-(4-(Dimethylamino)phenyl)-5-oxopyrrolidine-3-carboxamido)-5-oxopyrrolidine-3-carboxylic acid (8)

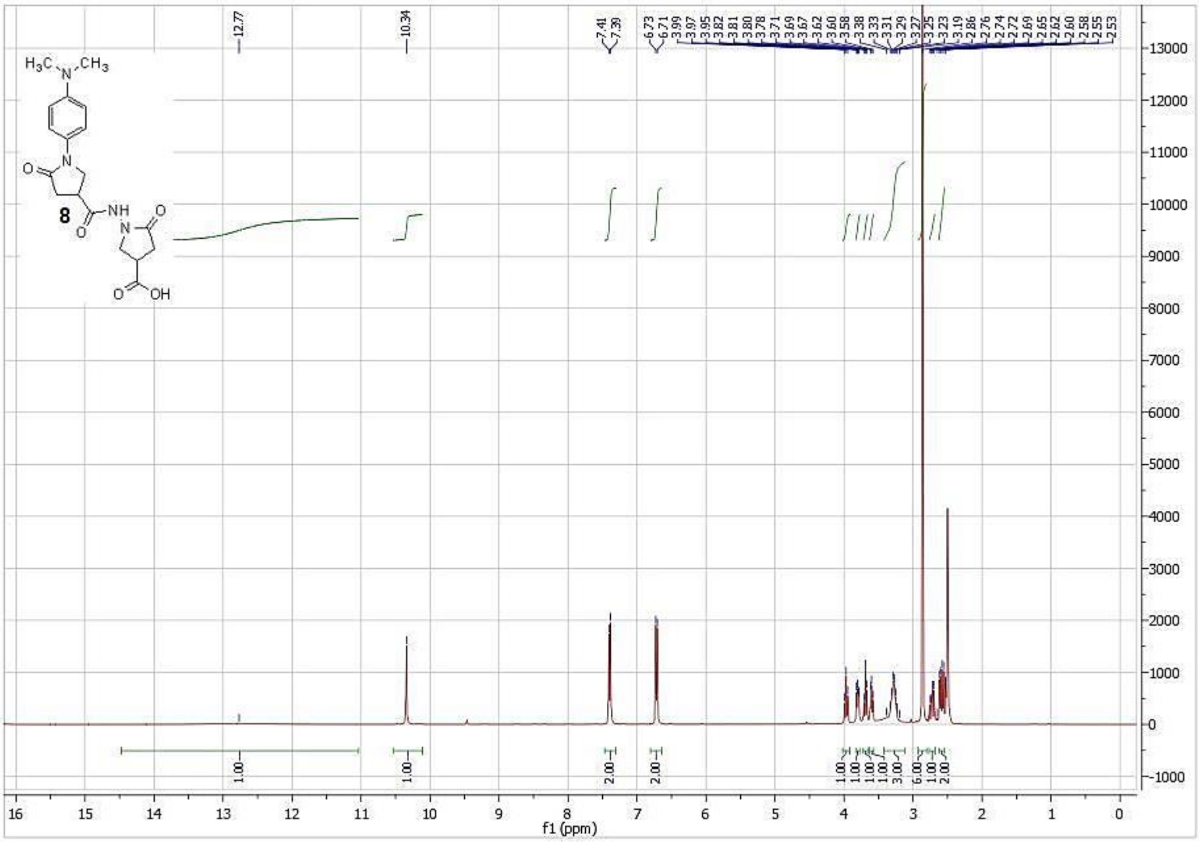

Figure S42. ^{1}H NMR spectrum of compound **8.**

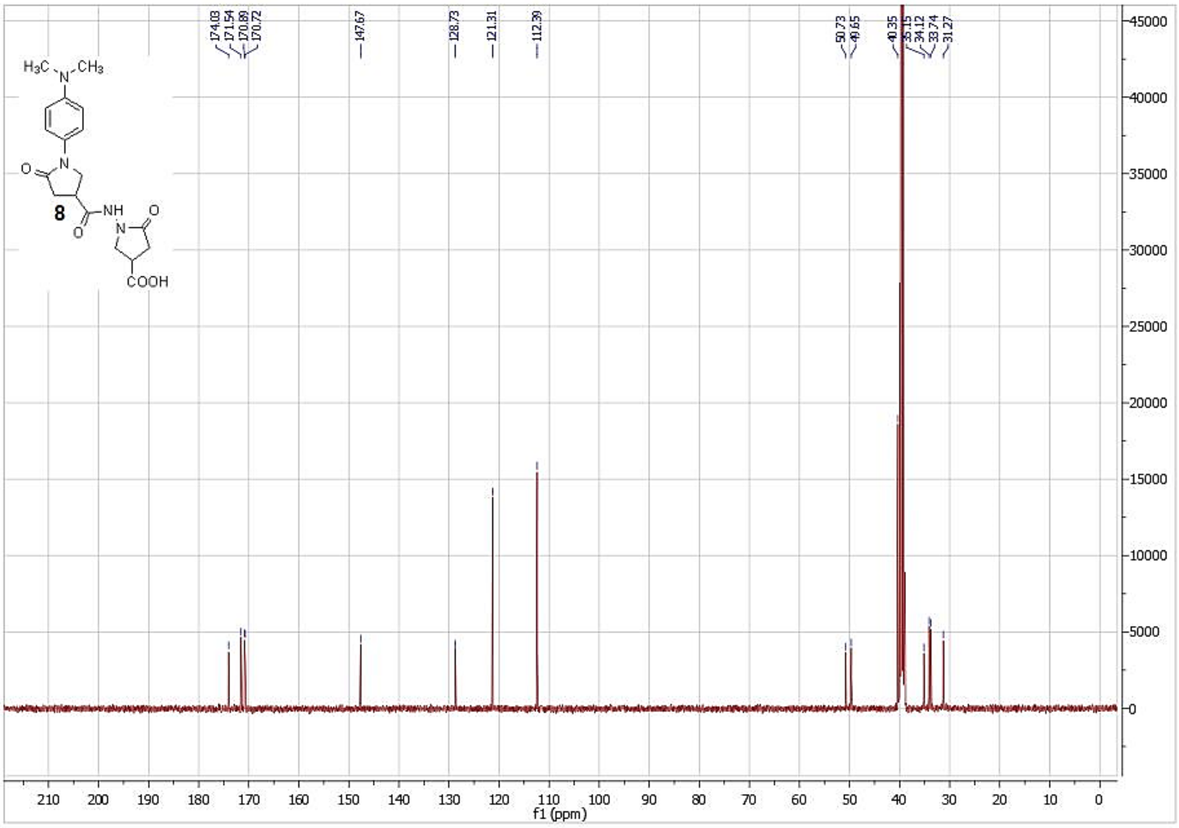

Figure S43. ^{13}C NMR spectrum of compound **8.**

1-(4-(Dimethylamino)phenyl)-5-oxo-N'-(2-oxoindolin-3-ylidene)pyrrolidine-3-carbohydrazide (9)

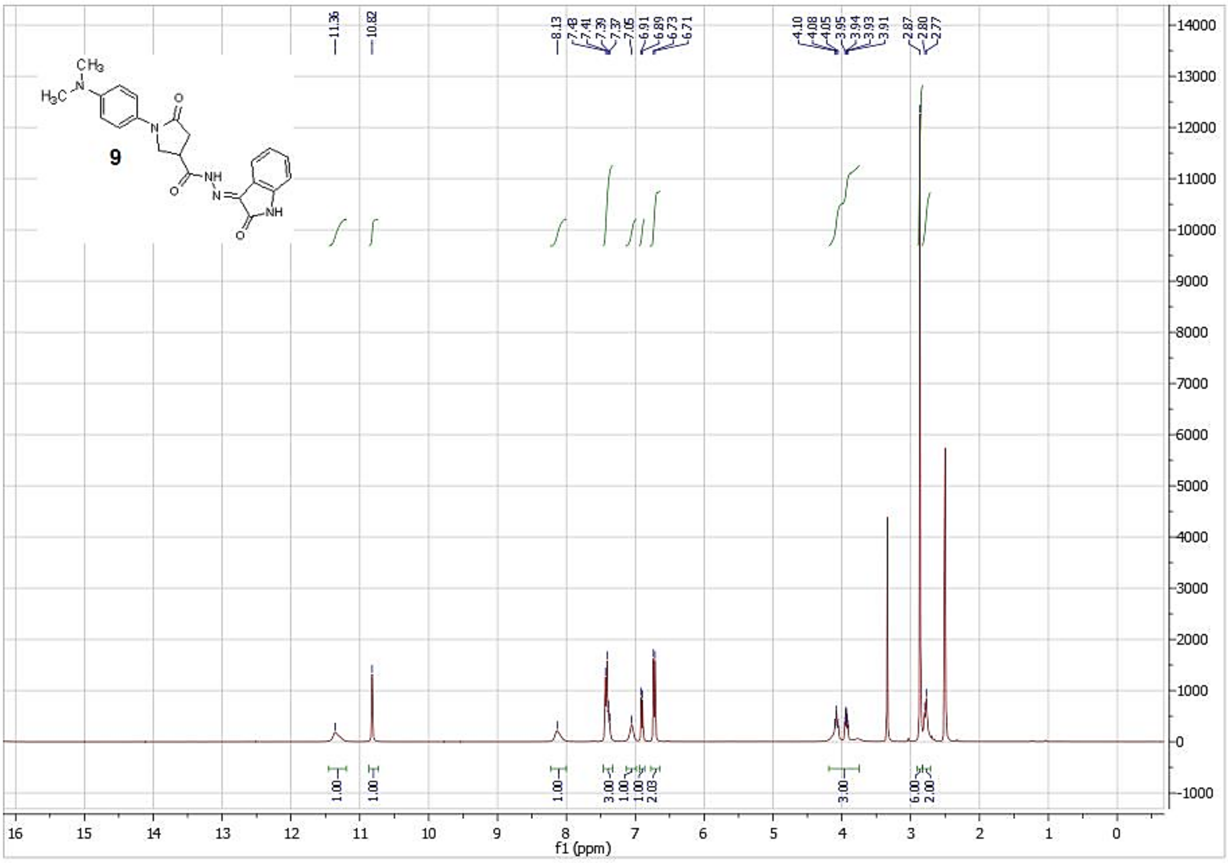

Figure S44. ^{1}H NMR spectrum of compound **9.**

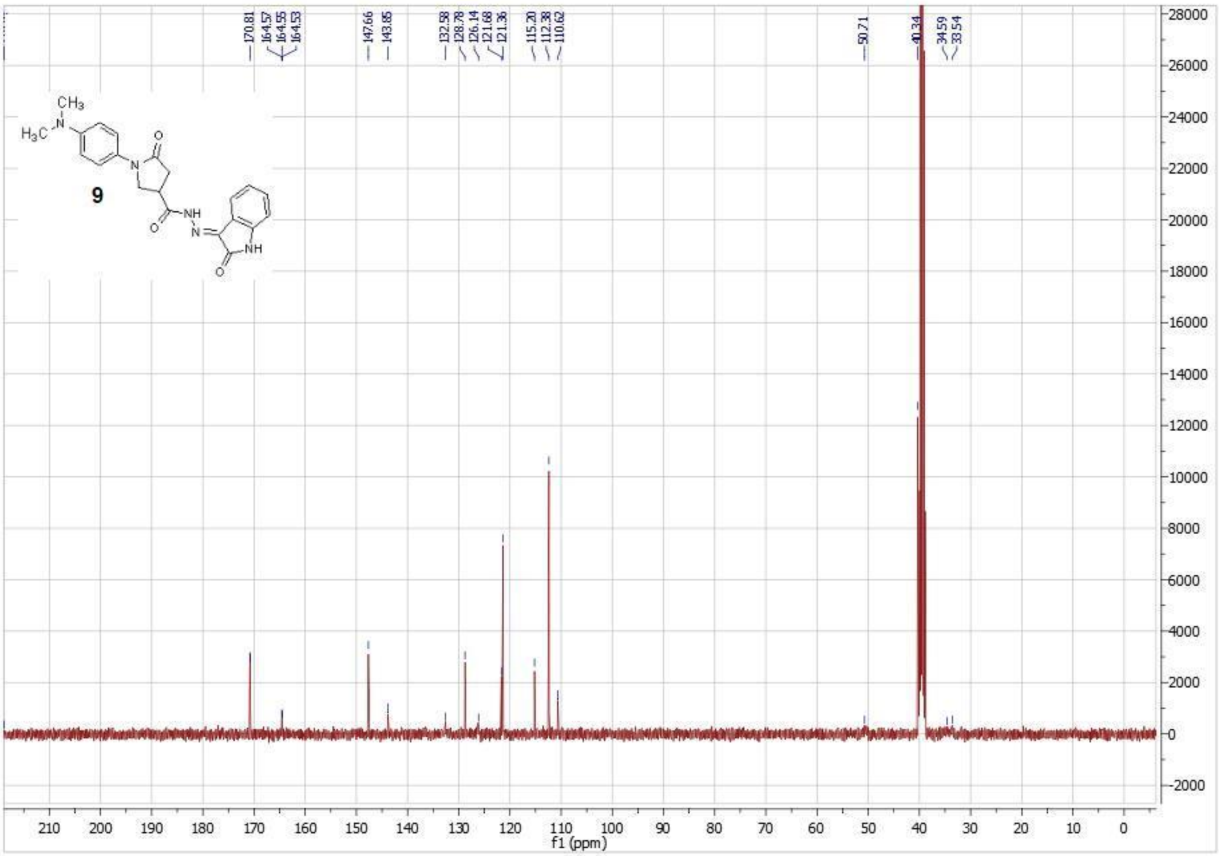

Figure S45. ^{13}C NMR spectrum of compound **9.**

1-(4-(Dimethylamino)phenyl)-4-(5-thioxo-4,5-dihydro-1,3,4-oxadiazol-2-yl)pyrrolidin-2-one (10)

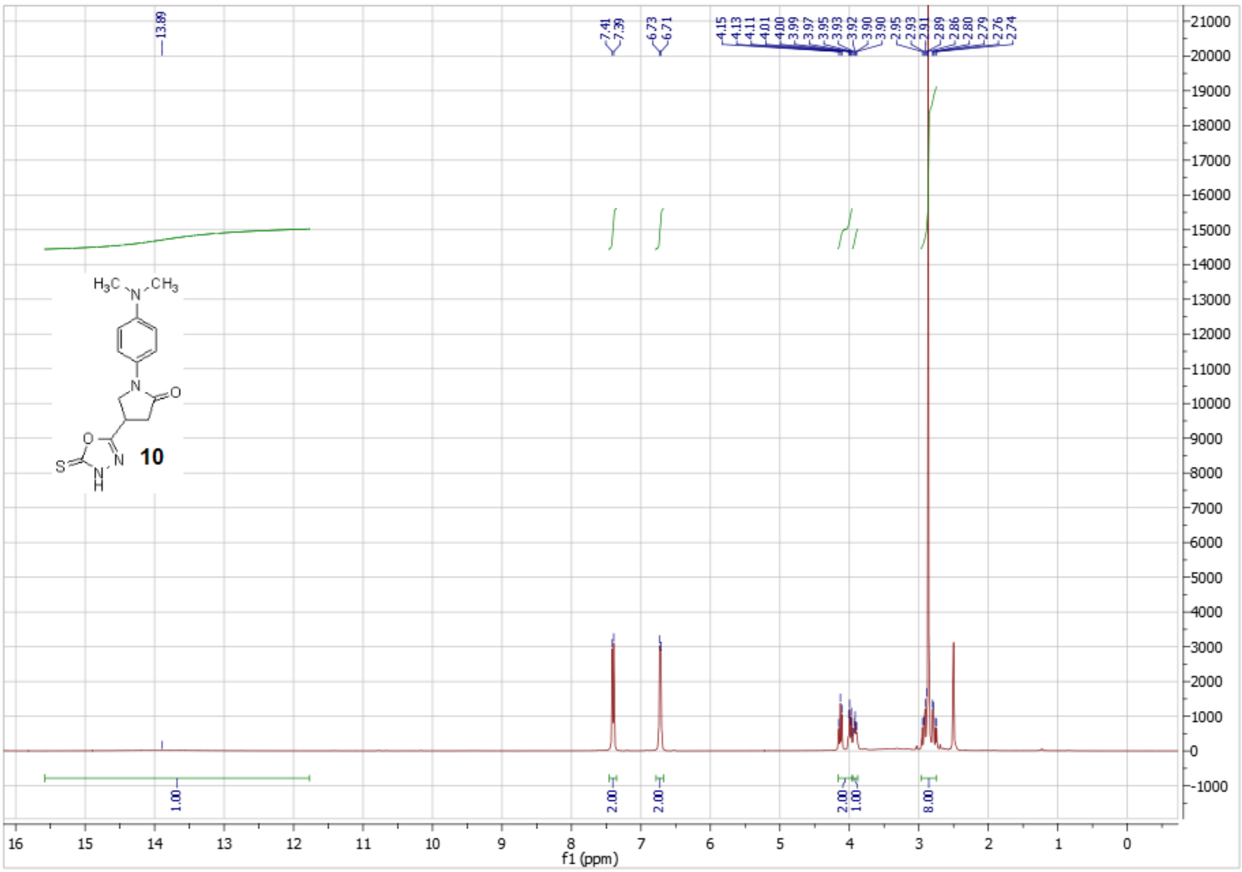

Figure S46. ^{1}H NMR spectrum of compound **10.**

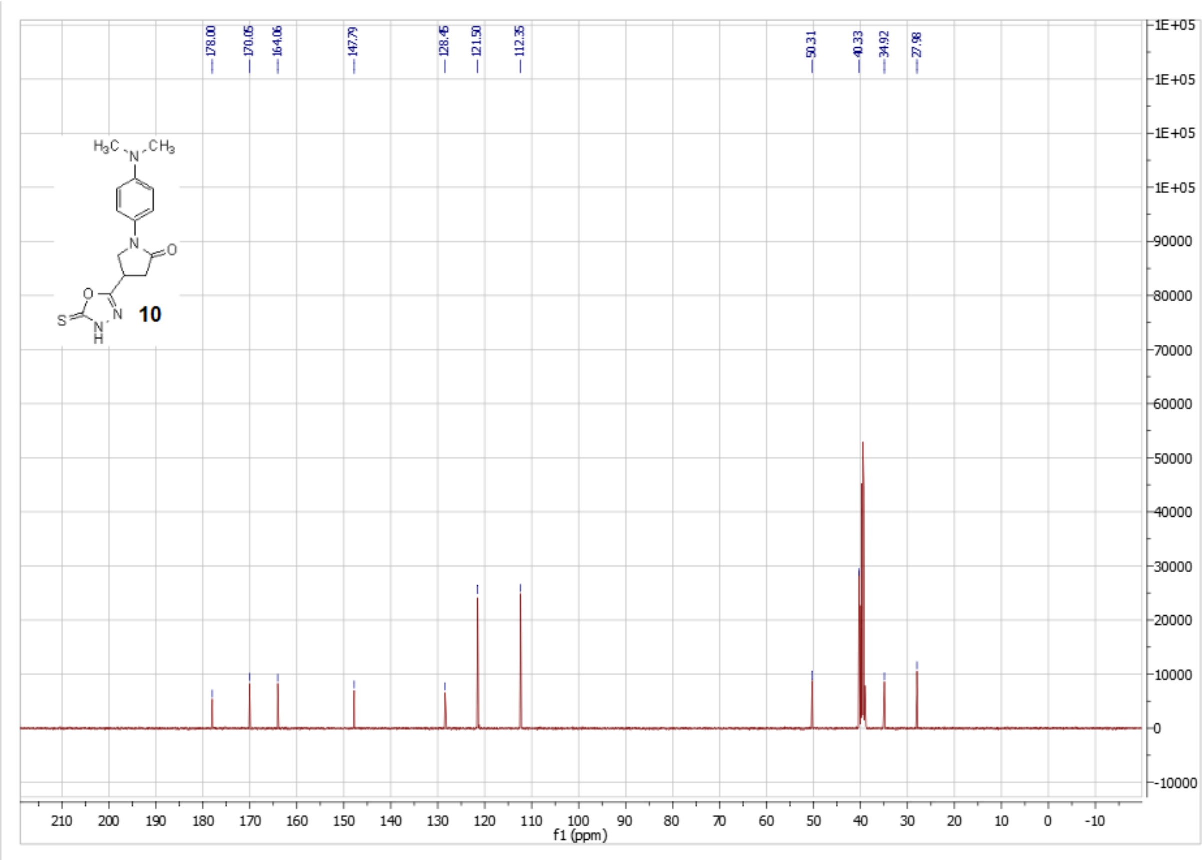

Figure S47. ^{13}C NMR spectrum of compound **10.**

1-(4-(Dimethylamino)phenyl)-4-(5,6-diphenyl-1,2,4-triazin-3-yl)pyrrolidin-2-one (11)

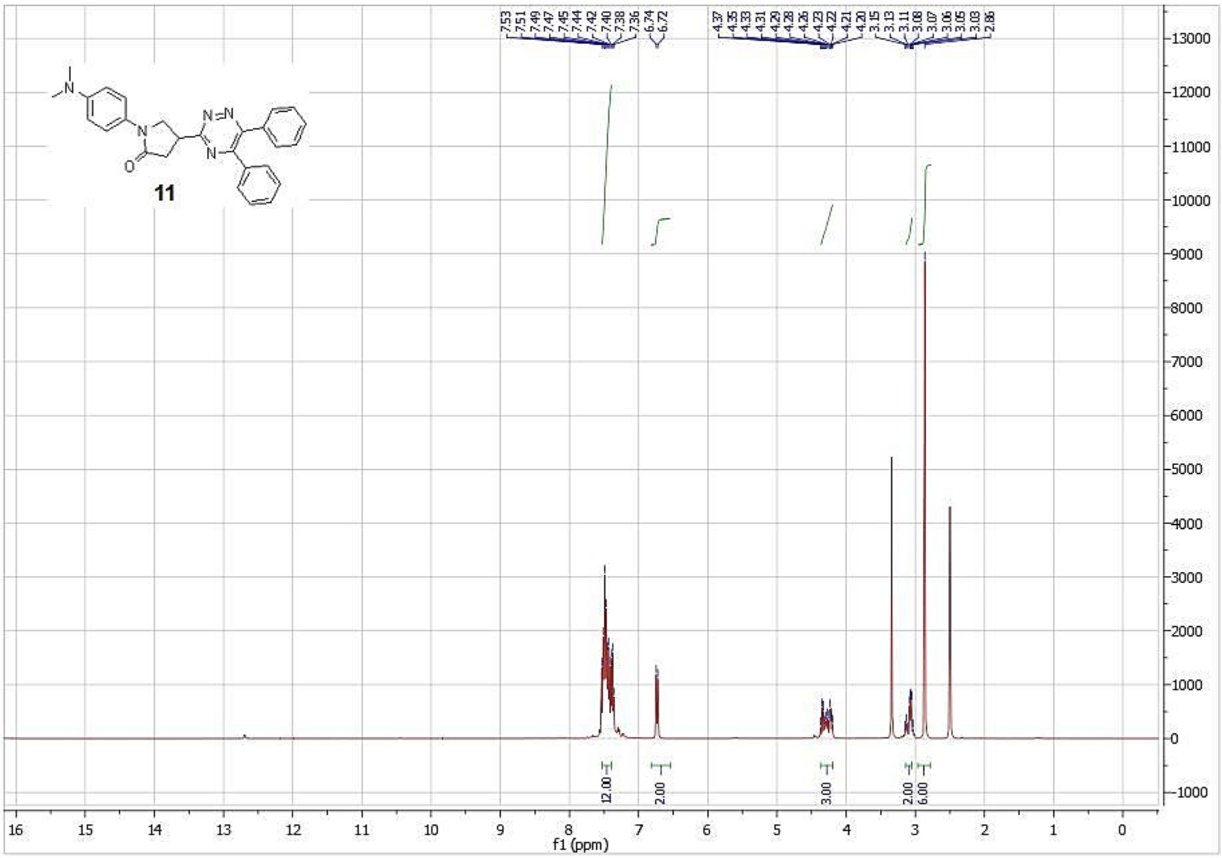

Figure S48. ^{1}H NMR spectrum of compound **11.**

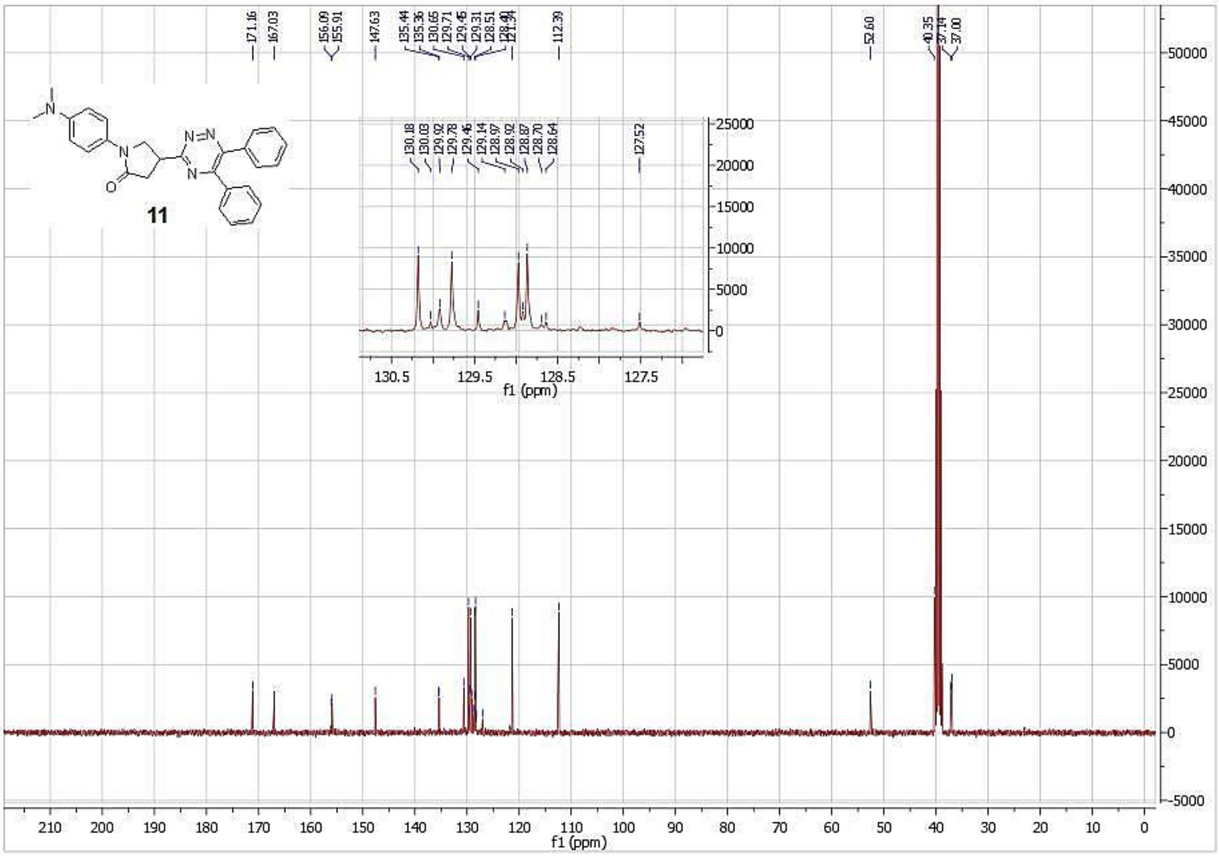

Figure S49. ^{13}C NMR spectrum of compound **11.**
